# Alternative reproductive adaptations predict asymmetric responses to climate change in lizards

**DOI:** 10.1038/s41598-019-41670-8

**Published:** 2019-03-25

**Authors:** Manuel Jara, Roberto García-Roa, Luis E. Escobar, Omar Torres-Carvajal, Daniel Pincheira-Donoso

**Affiliations:** 1 School of Life Sciences, University of Lincoln, Brayford Campus, Lincoln, LN6 7DL United Kingdom; 2 Department of Population Health and Pathobiology, College of Veterinary Medicine, North Carolina State University, Raleigh, NC USA; 3 Ethology Lab, Cavanilles Institute of Biodiversity and Evolutionary Biology, University of Valencia, Valencia, Spain; 4 Department of Fish and Wildlife Conservation, Virginia Tech, Blacksburg, Virginia USA; 5 Museo de Zoología, Escuela de Biología, Pontificia Universidad Católica del Ecuador, Avenida 12 de Octubre y Roca, Apartado 17-01-2184, Quito, Ecuador; 6 MacroBiodiversity Lab, School of Science and Technology, Department of Biosciences, Nottingham Trent University, Nottingham, NG11 8NS United Kingdom

## Abstract

Anthropogenic climate change ranks among the major global-scale threats to modern biodiversity. Extinction risks are known to increase via the interactions between rapid climatic alterations and environmentally-sensitive species traits that fail to adapt to those changes. Accumulating evidence reveals the influence of ecophysiological, ecological and phenological factors as drivers underlying demographic collapses that lead to population extinctions. However, the extent to which life-history traits influence population responses to climate change remains largely unexplored. The emerging ‘cul-de-sac hypothesis’ predicts that reptilian viviparity (‘live-bearing’ reproduction), a ‘key innovation’ facilitating historical invasions of cold climates, increases extinction risks under progressively warming climates compared to oviparous reproduction – as warming advances polewards/mountainwards, historically cold-climates shrink, leading viviparous species to face demographic collapses. We present the first large-scale test of this prediction based on multiple lizard radiations and on future projections of climate-based ecological niche models. Viviparous species were found to experience stronger elevational range shifts (and potentially increased extinctions) in coming decades, compared to oviparous lizards. Therefore, our analyses support the hypothesis’s fundamental prediction that elevational shifts are more severe in viviparous species, and highlight the role that life-history adaptations play in the responses of biodiversity to ongoing climate change.

## Introduction

The accelerated rates of climate change recorded over the last half century are known to be driving global-scale alterations and declines of biodiversity at unprecedented magnitudes^1–6^. As a result, accumulating evidence suggests that the planet is entering one of the greatest environmental crises since the origin of life^7–9^. A prevailing ‘biodiversity syndrome’ emerging from these climatic changes is the rapid alterations of the geographic ranges observed across a broad range of species^3,10–12^. Such geographic shifts are the result of species displacements tracking their rapidly moving historical niches as warming advances across space^10,13,14^. However, patterns, rates, and drivers of geographic range shifts differ across regions and lineages^10,14–16^. Climate change has consistently been observed to be spatially heterogeneous, tending to be more severe towards higher latitudes and elevations^3,10,14,15,17^. Therefore, species restricted to mountaintops and continental margins are more likely to experience progressive losses of suitable areas as warming advances, hence, forcing either rapid adaptations to the new climatic conditions, or extinctions^3,10,18,19^. The steepness of mountain slopes also increases the severity of climatic and ecological gradients in a more reduced geographic space (relative to flat regions), further aggravating the increases of extinction risks in species from high elevations^2,19–22^. In addition to pressures emerging from the environment, the magnitude of species’ responses to climate change importantly depends on their ecological tolerance as a function of intrinsic factors such as genetic variation, population size, and generation time^23–25^. In recent years, research efforts have focused on identifying mechanisms behind such spatial asymmetries in responses to contemporary climate change, leading to reinforce the focus on a range of phenological, ecological, and physiological factors as primary factors underlying extinction risks^26–28^.

Surprisingly, the influence that life-history adaptations exert on species responses to climate change has only occasionally been addressed. In fact, although parity-mode transitions (from live-bearing to egg-laying) have been suggested to play a key role in accelerating extinction risks under rapid climate warming^19,26^, only a handful of studies have investigated the ecological basis of this phenomenon^19,26,29^. Given that life-history traits underlie reproductive success (“fitness”), and that climate change operates as rapid natural selection on fitness via its impact on environmentally-sensitive traits, the conceptual disjoint between both dimensions creates a major gap in our understanding of biodiversity declines via anthropogenic effects. In fact, selection arising from climatic pressures have been shown to generate predictable patterns of evolution and distribution of reproductive traits (e.g., parity mode^19^, transient fecundity^30^), which therefore creates macroecological patterns of species interactions with the climate via life-history adaptations^19,30–33^.

An emerging idea, the ‘cul-de-sac’ hypothesis, predicts that the evolution of viviparity – a key life-history adaptation believed to have facilitated the radiation of reptiles into cold climates^19,33,34^ – is driving reptiles to extinctions due to climate change, relative to oviparous reproducers^19^. Thanks in part to evolving viviparity, reptiles have successfully proliferated across extreme high elevations and latitudes, where low temperatures create strong natural selection against multiple components of fitness via thermal demands on ecological (e.g., embryo survival and development^19,26^ and life-history (e.g., limitations on reproductive output for viviparous species) functions^26,32,35^. In contrast, these pressures are relaxed in warm climates, where oviparous reptiles dominate. In viviparous squamates, embryo retention in the maternal body acts similar to an incubator to counteract the lethal effects that low and fluctuating environmental temperatures impose on egg development^19,36–38^. However, cold-climate reptiles may be approaching an evolutionary dead-end in the face of climate change^19^. Given that most cold-climate reptiles are viviparous^30,32,39^, that the oviparity-to-viviparity transitions are largely irreversible (thus, fundamentally unidirectional)^19,32,39,40^, and that live-bearing parity (relative to oviparity) entails high fitness costs (e.g., reduced reproductive frequency, increased pregnancy-burden, prolonged basking time)^32,35,41^, the cul-de-sac hypothesis suggests that, as climate warming advances towards higher elevations and latitudes, viviparous reptiles will remain trapped in rapidly retracting cold environments^19^, while oviparous species are expected to colonize previously cold sites^19,26^. As viviparous species progressively run out of suitable environments as they approach mountaintops and continental edges, populations are expected to experience demographic collapse that will lead to extinctions^10,14,19,42^. So far, evidence consistent with this hypothesis remains limited to a few studies that have approached the question in a preliminary fashion^19,26,43^. However, a replicated, large-scale macroecological test assessing the responses of species to climate change as a function of life-history adaptations proposed by this hypothesis remains lacking.

Here, we present a comprehensive test of the cul-de-sac hypothesis, based on fine-scale distributional data spanning three prolific and evolutionarily contrasting lizard radiations that have diversified in the Andes and adjacent areas in South America. Their natural histories span all scenarios of parity mode variation: *Liolaemus*, with 270+ species is the world’s second most diverse genus of living amniotes^44^, in which viviparity has evolved in several independent phylogenetic events^19,45^; *Phymaturus*, a strictly cold-climate, viviparous genus of 60+ species^46^; finally, *Stenocercus* consists of 60+ oviparous species, despite their distributions up to above 4000 m in the Andes^47^. Using an ecological niche modeling framework and future climate scenarios (IPPC Fifth Assessment^48^) we tested whether viviparous (relative to oviparous) species (*i*) are predicted to experience greater range contractions, (*ii*) higher overall spatial displacements, and (*iii*) greater displacement towards higher elevations in relation with oviparous species.

## Results

**Figure 1:**
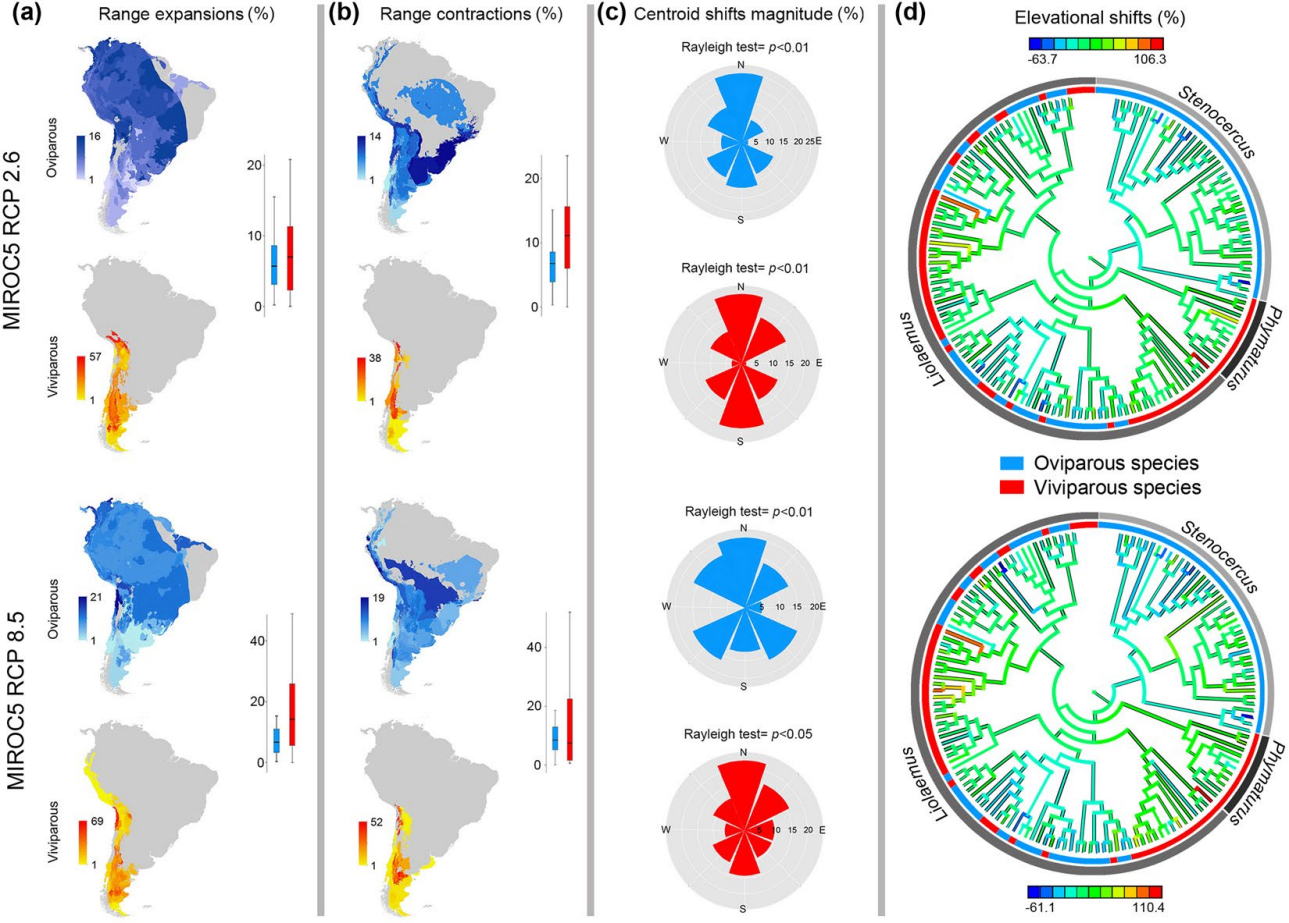
Predicted impacts of climate change on the distribution of species in study under the MIROC5 model, considering the minimum (RCP 2.6) and maximum (RCP 8.5) scenario of greenhouse gases emissions. Maps show: (**a**) expansions and (**b**) contractions of ranges, (**c**) variation in the direction of the geographic displacement and (**d**) elevational shifts represented in the phylogeny of species, where the external ring shows oviparous (blue) and viviparous (red) species. The maps were created using ArcGIS 10.4.1 (Esri, Redlands, CA).

Our ecological niche model (ENM) analyses reveal that the geographic ranges and their changes are predominantly influenced by temperature-related variables across all three lineages (in 93.7% of the species), while precipitation-related variables tend to be considerably less relevant (only dominant in 6.3% of the species; Supplementary Table S1). Also, we found that potential impacts of climate change on species ranges were not evenly distributed across space. Range expansions and contractions were more likely in certain regions (e.g., the high Andes), and absent from other regions (e.g., Patagonian steppe, Temperate broadleaf and mixed forests in Southern Chile; Moran’s spatial autocorrelation index >0.7, *P* < 0.001; Supplementary Table S8). Such range shifts patterns were observed consistently in viviparous species (Figs 1a,b and S1a,b). Although MIROC5-based models supported the hypothesis of a geographic directionality in the spatial shifts (Rayleigh’s test range, *P* = 0.05–0.01), GISS-ER-based models showed centroid shifts that were not distinguishable from uniform (Fig. 1c; Supplementary Table S6; Supplementary Fig. S1c).

### Geographic range shifts

**Figure 2:**
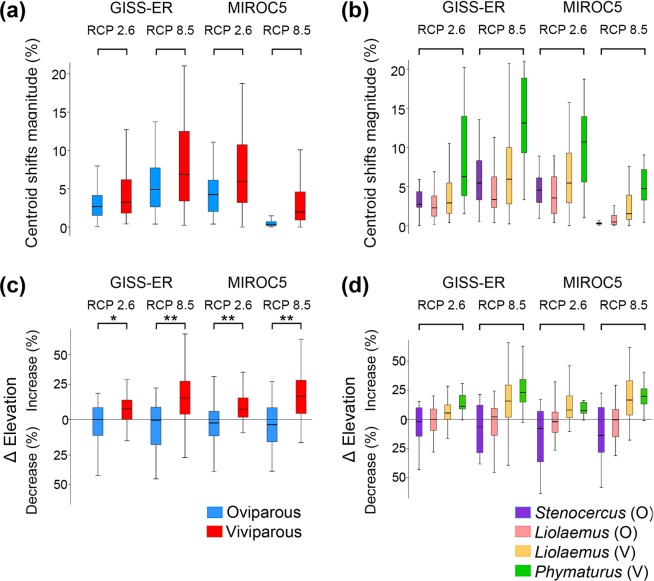
Climate change impacts on species geographic distributions. The magnitude of spatial displacements of range centroid between species with different parity mode (**a**), and the same measure among ‘parity-by-taxonomy’ groups (**b**). (**c**) Elevational shifts between species with different parity mode and (**d**) among ‘parity-by-taxonomy’ groups. Model results represent the minimum (RCP 2.6) and maximum (RCP 8.5) emission based on GISS-ER and MIROC5 climate models. Where, (**a**,**c**) show the results of the Independent-sample *t*-test, while b) and d) represent the Gabriel’s post hoc test results. For both analyses, *p < 0.05, **p < 0.01, ***p < 0.001).

Our analyses revealed that shifts in the percentage of range expansions or contractions driven by estimated magnitudes of climate change do not significantly differ between viviparous species combined (regardless of taxonomic group) and oviparous species combined (Table 1; Fig. 1a,b, Supplementary Tables S2–S5, Supplementary Fig. S1a,b). Likewise, the effects of climate change on the magnitude of spatial displacements of species’ geographic range centroids (Fig. 2a,b) and their spatial direction (Fig. 1c, Supplementary Table S6, Supplementary Fig. S1c) do not significantly differ between viviparous species combined and oviparous species combined, in concordance with observations on expansions and contractions.

**Table 1:** Pairwise comparison of the predicted climate change effects on species with different parity mode (i.e., oviparous and viviparous).

Model	Parameter	RCP 2.6		RCP 8.5	
		*t*_( *df*)_	*P*	*t*_( *df*)_	*P*
MIROC5	Range shifts (Total)	1.2 (141)	0.647	1.4 (141)	0.624
	Range contractions	15.9 (55)	0.191	2.5 (68)	0.717
	Range expansions	4.9 (85)	0.598	8.8 (72)	0.350
	Centroid shifts	1.5 (141)	0.611	0.3 (141)	0.820
	Elevational shifts	29.6 (141)	**<*0***.***01***	36.9 (141)	**<*0***.***01***
GISS-ER	Range shifts (Total)	2.6 (141)	0.497	7.1 (141)	0.252
	Range contractions	1.2 (66)	0.764	1.3 (60)	0.775
	Range expansions	2.6 (74)	0.334	16.6 (80)	0.224
	Centroid shifts	1.9 (141)	0.544	0.4 (141)	0.789
	Elevational shifts	20.1 (141)	***0***.***02***	29.9 (141)	**<*0***.***01***

Phylogenetic *t*-tests’ output showing the result for each parameter (represented by the impact of climate change on species distribution), considering two different general circulation models (GISS-ER and MIROC5) and two different scenarios of emissions (RCP 2.6 and 8.5).

In contrast, as predicted by theory, we found significant differences in the magnitude of elevational shifts between parity modes (*Liolaemus*-viviparous + *Phymaturus* versus *Liolaemus*-oviparous + *Stenocercus*; Fig. 2c,d), expressed as higher elevational shifts for viviparous species relative to oviparous species (Phylogenetic *t*-test range, *t*_141_ = 20.1–36.9, *P* = 0.02–0.004, Table 1; Fig. 2c). However, these differences in elevational shifts were not significant when compared among ‘parity-by-taxonomy’ groups (across *Stenocercus*, *Liolaemus*-oviparous, *Liolaemus*-viviparous and *Phymaturus*; Phylogenetic ANOVA range, *F*_3,139_ = 8.3–12.4, *P* = 0.27–0.41, Table 2, Fig. 2d). More specifically, under RCP 2.6 scenarios, *Liolaemus*-viviparous and *Phymaturus* lizards were found to have a projected increase in their elevational ranges of around 12% (relative to the present), and up to 21% for RCP 8.5 scenarios. In contrast, oviparous groups (*Stenocercus* and *Liolaemus*-oviparous) showed a decrease of ~2% in both RCPs scenarios (Fig. 1d; Supplementary Table S6). Pairwise comparisons showed that these differences remained consistent between *Liolaemus*-viviparous vs *Liolaemus*-oviparous species (Holm’s post-hoc test, *t* = 4.1–5.3, *P* = 0.001; Supplementary Table S7).

**Table 2:** Statistical phylogenetic comparison of the predicted impacts of climate change on the distribution of ‘parity-by-taxonomy’ groups (i.e., *Stenocercus*, *Liolaemus*-oviparous, *Liolaemus*-viviparous and *Phymaturus*).

Model	Parameter	RCP 2.6		RCP 8.5	
		*F*_( *df*)_	*P*	*F*_( *df*)_	*P*
MIROC5	Range shifts (Total)	0.9 (3,139)	0.931	0.6 (3,139)	0.949
	Range contractions	5.2 (3,65)	0.848	0.9 (3,65)	0.984
	Range expansions	1.6 (3,70)	0.961	3.1 (3,70)	0.909
	Centroid shifts	1.6 (3,139)	0.882	1.1 (3,139)	0.915
	Elevational shifts	9.8 (3,139)	0.365	12.4 (3,139)	0.271
GISS-ER	Range shifts (Total)	2.2 (3,139)	0.830	3.1 (3,139)	0.774
	Range contractions	2.7 (3,65)	0.922	1.6 (3,65)	0.948
	Range expansions	1.4 (3,70)	0.778	6.1 (3,70)	0.816
	Centroid shifts	1.2 (3,139)	0.909	0.9 (3,139)	0.936
	Elevational shifts	8.3 (3,139)	0.418	11.1 (3,139)	0.302

Phylogenetic ANOVA tests’ output showing the result for each parameter (represented by the impact of climate change on species distribution), considering two different general circulation models (GISS-ER and MIROC5) and two different scenarios of emissions (RCP 2.6 and 8.5).

### Future range overlaps between oviparous and viviparous species

Compared with the current range overlap between the geographic areas covered by oviparous and viviparous species combined regardless of taxonomy (currently 15.3%), our analyses suggest that climate change-driven range shifts are likely to cause an increase in the current levels of overlap between species with both parity modes. More specifically, range overlaps are projected to increase with the magnitude of greenhouse gas emissions (RCPs): while under the minimum climate change scenario (RCP 2.6), range overlap was observed to remain as today (~15% of overlap, relative to the modern 15.3%), under the maximum scenario (RCP 8.5) the predicted overlap is projected to increase up to 3%, leading to ~18% of overlap between both parity modes (Supplementary Table S9 and Fig. S2).

## Discussion

Our study presents the first comprehensive and broad-scale quantitative test of the ‘cul-de-sac’ hypothesis^19^, which predicts that viviparous species are more vulnerable to extinctions as climate warming progressively shrinks the cold regions they are adapted to^19^. Our findings across three highly-diverse lizard radiations support the effects that parity modes are expected to exert on the patterns of elevational shifts projected for these organisms as climate warms up (whereas, no such differences were observed for direction of shifts in range centroids). Specifically, our findings revealed that the prediction strongly holds for alterations in elevational distributions, where viviparous species combined experience greater elevational shifts relative to oviparous species (see Fig. 1d). These elevational displacements observed in viviparous species were stronger in *Phymaturus* (strictly viviparous), suggesting the potential for greater magnitudes of demographic impacts, and potentially extinction risks faced by these lizards under the ongoing climate trends^49^. Importantly, our findings also suggest that as the degrees of emissions of greenhouse gases increase, the extent of spatial overlap between oviparous and viviparous reproducers will increase, thus leading to the predicted scenarios of novel forms of competition between oviparous and viviparous species^19,26^. Collectively, therefore, our study reinforces the need to add the life-history dimension to the search for factors that trigger mechanisms underlying biodiversity alterations caused by increased anthropogenic climate change^19^. It also adds a further component to the sustained efforts to establish the combination of factors that define the ‘profile’ (i.e., shared combinations of traits) of species that have entered a current phase of extinction risk (or, on the other hand, of species which have seen their populations remain stable or even increase) as a result of environmental alterations.

At a more general level, the pathway towards species declines is a function of alterations to the ecological and demographic stability of populations, which can occur due to different mechanisms. For example, as a consequence of loss of their suitable distributional area, which can erode the genetic diversity to respond to rapidly changing selection regimes^42,50–52^. However, other species may not be experiencing shrinking of their geographic ranges, and yet, may still face demographic alterations via other mechanisms such as novel forms of competition with species in newly encountered assemblages^53^, or exposure to climatically suitable, but not necessarily structurally suitable (e.g., topographically) environments. Therefore, range shifts in diverse directions rather than towards higher latitudes (Fig. 1) are expected to impact on the ecological and demographic stability of those species by exposing them to novel environments and networks of species where selection regimes will be different. In some cases, these impacts may lead to population declines and thus, to increased risk of extinction via factors not necessarily connected directly with changing temperatures. For instance, a recent study on *Liolaemus* lizards showed that, in contrast with the traditionally established ‘cold-climate hypothesis’ that declines in environmental temperatures operate as drivers of natural selection for viviparity^32,54^, reductions in atmospheric oxygen levels play a central role as agents of selection, promoting embryo retention and evolution of viviparity^34^. Whether oxygen levels influence the magnitude and direction of alterations in range shifts of viviparous species via their interactions with thermal gradients remains an open question. However, this ‘hypoxia’ hypothesis contributes to the more general view that species’ range stability can be broken down into interactions between a range of external factors (e.g., climate, topography) and population features (e.g., abundance, morphology, life-history, physiology). Future studies could develop predictions about the effects of environmental factors on parity modes taking into account this strong role of oxygen, in combination with classic ecophysiological factors. Such predictions could expand the findings of, for example, Medina *et al*. Medina, *et al*.^29^, who observed that the preferred body temperature in the laboratory (T_*pref*_) does not differ among *Liolaemus* species with different parity models (while, however, field body temperature in the laboratory (T_*pref*_) does not differ among *Liolaemus* species with different parity models (while, however, field body temperature, T_*b*_, does).

This study adds a further conceptual and empirical layer to the urgent need to develop ‘profiles’ of species facing risks of extinction. These profiles aim to summarize our current understanding of factors that contribute to altering the historical stability of populations, and thus, to reinforce the efficiency of actions developed to mitigate ongoing declines of biodiversity. However, one of the main limitations of this study is the bias generated by not considering the extremely endemic species in our analysis, which is related to the lack of enough geographic information to predict the potential distribution of these species.

Overall, this study shows that life-history adaptations, favoured by natural selection, can turn into determinant factors pushing species to decline under contemporary climate change. Life-history traits, given their direct effects on fitness, are therefore primary candidate traits to disentangle factors that contribute to extinction, and thus must be incorporated into empirical and theoretical studies aiming to develop estimations of climate change effects on life on earth.

## Methods

### Species geographic distribution

We gathered a large-scale dataset encompassing three lizard radiations differing in their patterns of geographic distribution and in parity modes, which spans large species-samples of *Liolaemus* (40 oviparous and 52 viviparous species), *Phymaturus* (11 species, exclusively viviparous – although our dataset contained data for most species within this genus, we excluded most of them given their extremely limited geographic range sizes, which makes it inviable to perform the analyses employed to address our core predictions), and *Stenocercus* (40 species, exclusively oviparous) (Supplementary Fig. S1). The distributional and life-history data comprise 4,532 geographic occurrence records (after all duplicated points collected from different individuals at same localities were removed). This includes all known records of presence of these lizards following 20+ years of field and museum work, and museum-validated published occurrences^45,47,55–61^. All the occurrences were individually checked and confirmed by experts (i.e., Daniel Pincheira-Donoso: *Liolaemus* + *Phymaturus* and Omar Torres-Carvajal: *Stenocercus*, to assure accuracy).

### Environmental predictors

To analyse the environmental space occupied by lizard lineages, we used the bioclimatic variables characterizing climate during the 1970–2000 period, obtained from the WorldClim 2 data repository^62^ (available at: http://www.worldclim.org/version2) at a spatial resolution of 30 seconds (~1 km). To reduce collinearity between the environmental variables, we used VIF (Variance Inflation Factors) implemented in the “usdm” R-package^63^. Using this approach, we excluded all the highly correlated variables from the model (VIF greater than 10), which is associated with a signal that the model has a collinearity problem^64^. This method is based on the square of the multiple correlation coefficient (*R*^2^) resulting from regressing the predictor variable against all other predictor variables. We used the remaining uncorrelated variables to calibrate models: mean annual temperature (bio1), mean diurnal range (bio2), isothermality (bio3), temperature seasonality (bio4), max temperature of warmest month (bio5), min temperature of coldest month (bio6), temperature annual range (bio7), mean temperature of warmest quarter (bio10), mean temperature of coldest quarter (bio11), annual precipitation (bio12) precipitation of wettest month (bio13), precipitation of driest month (bio14), and precipitation seasonality (bio15), precipitation of wettest quarter (bio16), and precipitation of driest quarter (bio17).

To explore the occupation of environmental space by *Liolaemus*, *Phymaturus*, and *Stenocercus* and their reproductive modes, we performed a principal component analysis (PCA) of present-day climate conditions in South America using NicheA v3.0 software^65^ (available at: http://nichea.sourceforge.net), an open-source application that analyses ecological niches (or ‘climatic spaces’) in both environmental and geographic space (Supplementary Fig. S3a,b).

To estimate impacts of climate change on distributions of species, we employed two models of future climate conditions, the GISS-ER and the MIROC5 general circulation models for the period around 2070 (2061–2080). Each model consists of two Representative Concentration Pathways (RCPs) versions representing a stringent mitigation scenario (RCP2.6, which predicts magnitude of climatic variation for the parameters of the model) and one scenario assuming high anthropogenic greenhouse gas (GHG) emissions (RCP8.5, which predicts the highest magnitude of climatic change for the parameters of the model) as alternative scenarios of climate change^66^ (IPCC, Fifth Assessment), because it is extremely likely that human activities caused more than half of the observed increase in GMST (Global Mean Surface Temperature) from 1951 to 2010^67^. We selected these models based on their high resolution and incorporation of covariates^68,69^.

### Species distribution modeling

Based on the occurrence data (Supplementary Fig. 1), we calibrated Ecological Niche Models (ENMs) to estimate current and future potential distribution for each lizard species. To mitigate oversampling effects in our model, occurrences were re-sampled to one point per pixel with respect to the environmental grids. Coordinates were divided into two groups for calibration and other evaluation, based on four quadrants with similar numbers of points, using two off-diagonal quadrants for calibration and two for evaluation. ENMs were developed using Maxent 3.3.3k^70^, a presence-background software that estimates environmental suitability via an index of similarity that resembles a heterogeneous occurrence process or logistic regression function^70,71^. We used Maxent with clamping and extrapolation turned off (i.e., no prediction outside the range of environmental conditions in the calibration areas). To facilitate interpretations, we used the relative probability of presence as a proxy of environmental suitability^71^. Suitable area for each species was estimated as a Boolean (presence/absence) map that was thresholded based on the minimum training presence^72^. To determine the model parametrization with the best fit to the data available, we assessed six models for each species under different regularization multiplier values (i.e., 0.5, 1, 5, 10, 15, and 20)^73^. Then, we assessed AIC (Akaike Information Criterion; implemented in ENMTools^74^) values to choose the model with the best performance (i.e., descriptive-model evaluation; Supplementary Table S10). Once the models with the best fit were selected, we assessed their performance using the software PartialROC^74^, considering an α = 0.05 to determine whether models anticipate independent occurrences better than by random expectations.

To determine changes between the current and future potential distributions, we identified the expansion, contraction, and stability (‘no change’) of areas predicted climatically suitable by using the SDM toolbox in ArcGIS v.10.2^75^. Direction of the potential distributional changes was calculated based on the centroid of the species’ potential ranges. We applied Rayleigh’s Test of Uniformity^76^ to compare direction of centroid shifts vs a uniform circular distribution reflecting a null hypothesis of random centroid shifts. Rayleigh’s statistic quantifies the angular dispersion among the vectors from 0 (representing uniform dispersion) to 1 (indicating complete concentration in a single direction).

Finally, to determine the spatial relationships between oviparous and viviparous species, we quantified the range overlap among the predicted distributions of oviparous and viviparous species. These calculations were made using the predicted present-day and future SDMs under GISS-ER and the MIROC5 models and each greenhouse emission scenarios (RCP 2.6 and 8.5).

### Climate change impacts on life-histories

To determine effects of climate change on parity mode, we performed an independent-sample *t*-test using the variables: (i) range extents of current and future ENMs, (ii) magnitude and direction of distributions based on range centroids, and (iii) average elevation of current and future ranges. Analyses were corrected for phylogenetic non-independence to test whether the predicted impacts of climate change (i.e., as geographic range, centroid or elevational shifts) were statistically significant between species with different parity mode (i.e., oviparous and viviparous), as well as among ‘parity-by-taxonomy’ groups (i.e., *Stenocercus*, *Liolaemus*-oviparous, *Liolaemus*-viviparous and *Phymaturus*) using the fully-sampled phylogeny of Squamates recently published by Tonini, *et al*.^77^ which spans all 143 species in our dataset. We performed phylogenetic analyses (*t*-test and ANOVA) implemented in the R-package “phytools”^78^. Additionally, to calculate the differences between each independent pair of groups we applied ANOVA test with “holm” post-hoc pairwise method, owing to it is one of the most powerful multitests when the sample sizes of the groups are different, to perform this analysis, we used the R-package “DescTools”. These analyses were performed for the two models of climate change (GISS-ER and MIROC5) and for each scenario of greenhouse gases emissions indicated above (RCP 2.6 and 8.5). Finally, we performed a spatial autocorrelation analysis (Global Moran’s I) using the Spatial Statistics toolbox in ArcGIS to evaluate whether geographic rage shifts were clustered, dispersed, or randomly distributed.

## Supplementary information

Supplementary material
