# Supplementary material for: Alternative reproductive adaptations predict asymmetric responses to climate change in lizards
Source: Sci Rep. 2019 Mar 25;9:5093. doi: 10.1038/s41598-019-41670-8 (PMC6433898; doi:10.1038/s41598-019-41670-8)
Supplement: Supplementary file 1 — Supplementary material [file 41598_2019_41670_MOESM1_ESM.doc]

**Alternative reproductive adaptations predict asymmetric responses to climate change in lizards**

Jara, García-Roa, Escobar, Torres-Carvajal and Pincheira-Donoso

**SUPPLEMENTARY MATERIAL**

**Table S1.** **Decomposition of the explanatory strength that each environmental variable plays on the geographic ranges (and their shifts) for each species in our analyses according to Ecological Niche Models (ENM).** The explanatory strength of each variable is indicated as percentage (relative to the other variables), and values have been colour-coded to facilitate visual delivery of the results (intense blue is minimum %, light colours are for intermediate values, and intense red is maximum %). Parity modes are 1=oviparous and 2= viviparous. Environmental variables are described in the methods. The lizard genera are *Liolaemus* (L), *Phymaturus* (P) and *Stenocercus* (S).

| Species | Parity Mode | Environmental Variables | | | | | | | | | | | | | | |
| --- | --- | --- | --- | --- | --- | --- | --- | --- | --- | --- | --- | --- | --- | --- | --- | --- |
|  |  | bio1 | bio2 | bio3 | bio4 | bio5 | bio6 | bio7 | bio10 | bio11 | bio12 | bio13 | bio14 | bio15 | bio16 | bio17 |
| *L. abaucan* | 1 | 2.2 | 12.5 | 7.3 | 6.3 | 0.0 | 33.7 | 3.3 | 0.0 | 6.0 | 18.0 | 5.4 | 1.3 | 0.8 | 0.7 | 2.5 |
| *L. albiceps* | 2 | 11.0 | 16.6 | 2.8 | 22.9 | 0.0 | 14.8 | 6.9 | 0.0 | 0.0 | 0.8 | 0.0 | 7.2 | 0.0 | 0.0 | 17.0 |
| *L. alticolor* | 2 | 34.9 | 3.1 | 4.3 | 3.6 | 1.3 | 1.0 | 14.3 | 4.7 | 0.0 | 0.2 | 15.8 | 0.0 | 0.0 | 16.8 | 0.0 |
| *L. andinus* | 2 | 27.3 | 0.6 | 6.9 | 3.1 | 1.0 | 38.0 | 2.4 | 0.4 | 4.3 | 4.8 | 5.1 | 0.0 | 1.5 | 0.4 | 4.2 |
| *L. anomalus* | 1 | 4.0 | 0.4 | 44.6 | 27.7 | 0.2 | 0.0 | 8.9 | 0.1 | 0.7 | 1.0 | 3.8 | 7.2 | 1.4 | 0.0 | 0.0 |
| *L. archeforus* | 2 | 19.5 | 0.3 | 10.1 | 5.3 | 4.9 | 51.8 | 0.0 | 0.0 | 0.0 | 0.0 | 0.0 | 0.0 | 5.3 | 0.7 | 2.1 |
| *L. atacamensis* | 1 | 11.2 | 19.7 | 0.0 | 0.0 | 9.0 | 21.1 | 25.2 | 0.0 | 0.0 | 7.5 | 1.6 | 0.0 | 1.9 | 0.0 | 2.8 |
| *L. audituvelatus* | 2 | 7.6 | 11.6 | 1.7 | 0.0 | 10.4 | 19.5 | 13.6 | 0.0 | 1.3 | 1.7 | 2.1 | 0.0 | 0.4 | 30.1 | 0.0 |
| *L. austromendocinus* | 2 | 2.4 | 43.1 | 7.7 | 0.0 | 19.3 | 1.0 | 1.7 | 0.0 | 8.6 | 0.1 | 0.0 | 11.4 | 4.7 | 0.0 | 0.0 |
| *L. bellii* | 2 | 8.9 | 6.3 | 4.8 | 0.2 | 9.3 | 35.4 | 0.0 | 6.4 | 0.9 | 0.0 | 8.2 | 0.0 | 8.9 | 0.0 | 10.7 |
| *L. bibronii* | 1 | 30.1 | 7.5 | 0.7 | 35.4 | 1.4 | 1.8 | 6.9 | 0.8 | 1.4 | 1.2 | 3.4 | 0.0 | 3.5 | 0.1 | 5.8 |
| *L. bitaeniatus* | 1 | 10.2 | 0.0 | 29.8 | 0.2 | 15.0 | 38.4 | 0.0 | 0.0 | 0.0 | 0.0 | 0.0 | 0.0 | 0.0 | 6.4 | 0.0 |
| *L. boulengeri* | 1 | 0.0 | 13.8 | 14.2 | 24.5 | 39.1 | 0.0 | 0.0 | 0.0 | 0.7 | 0.3 | 1.0 | 3.3 | 0.2 | 2.4 | 0.5 |
| *L. buergeri* | 2 | 4.7 | 24.1 | 10.3 | 5.7 | 1.3 | 28.6 | 0.0 | 0.0 | 8.9 | 0.0 | 1.1 | 8.9 | 0.1 | 4.9 | 1.4 |
| *L. canqueli* | 1 | 10.3 | 12.2 | 13.7 | 3.6 | 0.2 | 29.7 | 19.9 | 3.1 | 0.0 | 6.8 | 0.2 | 0.1 | 0.0 | 0.0 | 0.2 |
| *L. ceii* | 2 | 4.6 | 8.1 | 22.7 | 10.2 | 3.3 | 16.7 | 5.3 | 4.6 | 0.4 | 10.6 | 9.1 | 2.2 | 0.0 | 0.0 | 2.2 |
| *L. chacoensis* | 1 | 10.1 | 4.3 | 10.7 | 12.1 | 1.6 | 36.4 | 1.6 | 0.0 | 0.0 | 1.4 | 0.2 | 11.7 | 7.2 | 0.9 | 1.8 |
| *L. chaltin* | 1 | 9.7 | 32.4 | 14.1 | 7.7 | 1.2 | 12.3 | 12.4 | 0.3 | 1.5 | 1.1 | 2.7 | 1.3 | 0.3 | 2.4 | 0.6 |
| *L. chiliensis* | 1 | 0.4 | 2.5 | 6.9 | 0.3 | 7.5 | 9.8 | 30.1 | 0.8 | 2.9 | 4.4 | 11.7 | 3.6 | 18.3 | 0.2 | 0.6 |
| *L. constanzae* | 1 | 10.2 | 5.2 | 14.7 | 6.2 | 0.1 | 21.3 | 5.9 | 0.0 | 0.2 | 0.0 | 10.2 | 4.3 | 0.5 | 11.9 | 9.3 |
| *L. curicensis* | 2 | 25.8 | 4.9 | 16.2 | 3.2 | 5.8 | 18.9 | 6.4 | 1.7 | 0.0 | 11.8 | 0.3 | 0.1 | 0.8 | 1.8 | 2.3 |
| *L. curis* | 2 | 11.2 | 37.2 | 0.0 | 2.5 | 0.1 | 1.4 | 6.9 | 0.0 | 0.5 | 23.5 | 1.7 | 3.7 | 4.1 | 0.0 | 7.2 |
| *L. cuyanus* | 1 | 0.1 | 10.9 | 10.2 | 29.3 | 0.0 | 16.1 | 5.4 | 0.0 | 0.0 | 10.2 | 0.0 | 16.1 | 0.3 | 0.3 | 1.1 |
| *L. cyanogaster* | 2 | 18.7 | 18.6 | 0.0 | 2.1 | 15.0 | 0.0 | 11.1 | 0.0 | 3.6 | 0.0 | 9.9 | 11.2 | 0.0 | 8.0 | 1.8 |
| *L. darwinii* | 1 | 1.3 | 26.3 | 1.8 | 2.1 | 0.6 | 13.9 | 3.4 | 7.3 | 10.4 | 8.7 | 0.0 | 5.8 | 3.6 | 5.7 | 9.1 |
| *L. elongatus* | 2 | 11.8 | 5.2 | 13.2 | 6.7 | 4.5 | 10.3 | 0.1 | 1.5 | 16.7 | 2.3 | 10.5 | 2.4 | 14.3 | 0.4 | 0.1 |
| *L. escarchadosi* | 2 | 5.4 | 10.0 | 8.9 | 18.9 | 3.2 | 6.8 | 15.6 | 1.2 | 15.7 | 0.9 | 7.3 | 0.0 | 5.1 | 1.0 | 0.0 |
| *L. fabiani* | 2 | 8.6 | 5.6 | 4.6 | 1.2 | 13.2 | 10.0 | 8.0 | 1.1 | 0.0 | 18.6 | 0.0 | 3.6 | 13.1 | 12.1 | 0.3 |
| *L. fitzgeraldi* | 2 | 25.7 | 0.0 | 28.9 | 0.0 | 4.4 | 0.2 | 3.7 | 0.0 | 0.0 | 11.5 | 7.3 | 1.3 | 6.4 | 0.0 | 10.6 |
| *L. fitzingerii* | 1 | 0.1 | 18.8 | 30.9 | 6.9 | 10.6 | 10.3 | 0.2 | 0.1 | 3.5 | 1.8 | 0.0 | 0.2 | 7.2 | 0.3 | 9.1 |
| *L. fuscus* | 1 | 9.6 | 28.9 | 15.1 | 5.2 | 1.2 | 13.7 | 2.7 | 9.9 | 0.2 | 0.5 | 3.2 | 9.5 | 0.0 | 0.1 | 0.2 |
| *L. gallardoi* | 2 | 13.1 | 9.3 | 33.2 | 0.1 | 0.2 | 30.1 | 0.3 | 0.1 | 1.8 | 0.1 | 4.5 | 1.2 | 0.0 | 4.7 | 1.3 |
| *L. goetschi* | 1 | 14.3 | 8.7 | 9.4 | 11.1 | 15.9 | 13.0 | 5.4 | 6.6 | 0.0 | 1.3 | 7.8 | 0.2 | 5.4 | 0.2 | 0.7 |
| *L. gracilis* | 1 | 21.1 | 27.6 | 1.9 | 0.8 | 4.1 | 0.3 | 1.7 | 6.8 | 0.0 | 0.7 | 7.5 | 6.2 | 19.3 | 1.8 | 0.2 |
| *L. gravenhorsti* | 2 | 2.0 | 2.1 | 18.4 | 1.4 | 47.3 | 4.6 | 0.0 | 7.9 | 0.0 | 3.6 | 11.4 | 1.2 | 0.0 | 0.0 | 0.1 |
| *L. hatcheri* | 2 | 30.5 | 5.9 | 29.8 | 0.0 | 0.0 | 0.1 | 0.0 | 0.0 | 6.4 | 16.0 | 6.2 | 0.0 | 1.1 | 3.9 | 0.1 |
| *L. irregularis* | 2 | 6.4 | 3.8 | 7.3 | 36.4 | 2.9 | 2.2 | 8.3 | 0.9 | 7.4 | 6.6 | 0.0 | 13.7 | 0.4 | 0.2 | 3.5 |
| *L. isabelae* | 2 | 6.7 | 0.0 | 1.0 | 38.3 | 0.0 | 0.5 | 24.6 | 0.0 | 0.0 | 3.5 | 1.1 | 5.2 | 3.7 | 6.3 | 9.1 |
| *L. jamesi* | 2 | 15.7 | 18.0 | 0.6 | 15.0 | 0.2 | 26.8 | 0.4 | 0.1 | 2.0 | 4.8 | 5.1 | 0.0 | 1.8 | 0.0 | 9.5 |
| *L. kingii* | 2 | 0.2 | 32.5 | 3.0 | 26.1 | 8.1 | 2.0 | 0.0 | 0.6 | 0.1 | 2.8 | 0.4 | 2.5 | 0.8 | 14.1 | 6.8 |
| *L. koslowskyi* | 1 | 20.1 | 1.1 | 4.7 | 9.5 | 2.3 | 10.2 | 30.7 | 1.0 | 0.8 | 4.8 | 0.0 | 5.9 | 7.5 | 0.6 | 0.8 |
| *L. kriegi* | 2 | 24.6 | 11.3 | 4.1 | 18.5 | 10.1 | 21.1 | 0.0 | 0.0 | 3.0 | 0.8 | 0.0 | 0.1 | 4.2 | 0.9 | 1.3 |
| *L. lavillai* | 2 | 15.6 | 9.6 | 1.1 | 6.8 | 6.4 | 20.5 | 5.6 | 6.1 | 0.4 | 1.5 | 0.1 | 0.0 | 5.6 | 5.6 | 15.1 |
| *L. lemniscatus* | 1 | 0.7 | 1.7 | 4.3 | 21.9 | 11.1 | 15.2 | 2.2 | 8.8 | 0.4 | 1.3 | 11.0 | 0.2 | 10.2 | 10.9 | 0.1 |
| *L. leopardinus* | 2 | 22.2 | 10.8 | 9.9 | 2.8 | 1.6 | 28.9 | 0.0 | 0.0 | 0.0 | 9.6 | 0.0 | 3.6 | 10.5 | 0.0 | 0.1 |
| *L. lineomaculatus* | 2 | 0.6 | 4.9 | 0.5 | 33.1 | 30.2 | 0.2 | 11.1 | 2.7 | 2.2 | 4.0 | 3.3 | 3.2 | 0.6 | 3.4 | 0.0 |
| *L. lutzae* | 1 | 16.3 | 29.8 | 6.4 | 10.2 | 9.5 | 11.7 | 4.7 | 1.5 | 2.0 | 0.3 | 0.0 | 0.1 | 0.8 | 1.7 | 5.0 |
| *L. magellanicus* | 2 | 22.1 | 6.2 | 6.9 | 19.0 | 0.1 | 7.9 | 14.4 | 0.7 | 0.0 | 0.3 | 14.3 | 0.3 | 7.4 | 0.0 | 0.4 |
| *L. manueli* | 2 | 4.3 | 9.9 | 3.8 | 13.9 | 30.2 | 3.8 | 5.7 | 3.7 | 3.1 | 2.1 | 1.7 | 1.9 | 14.5 | 0.2 | 1.2 |
| *L. melanops* | 1 | 12.8 | 18.2 | 7.2 | 2.1 | 0.7 | 30.6 | 2.3 | 0.2 | 3.6 | 1.1 | 5.0 | 11.1 | 0.7 | 3.1 | 1.3 |
| *L. monticola* | 1 | 14.9 | 20.5 | 5.8 | 8.3 | 2.3 | 19.1 | 0.1 | 0.0 | 0.0 | 0.3 | 12.0 | 16.2 | 0.0 | 0.1 | 0.4 |
| *L. multicolor* | 2 | 0.5 | 0.3 | 16.6 | 30.4 | 8.2 | 12.9 | 6.7 | 1.2 | 0.0 | 7.5 | 0.3 | 7.2 | 4.4 | 2.3 | 1.5 |
| *L. multimaculatus* | 1 | 1.5 | 45.0 | 6.1 | 1.7 | 0.3 | 28.2 | 0.5 | 0.1 | 0.0 | 1.2 | 0.0 | 0.9 | 14.4 | 0.0 | 0.1 |
| *L. nigriceps* | 2 | 0.1 | 28.3 | 9.2 | 7.5 | 37.6 | 2.8 | 2.4 | 1.6 | 0.0 | 7.4 | 0.0 | 0.9 | 0.0 | 0.4 | 1.8 |
| *L. nigromaculatus* | 1 | 11.9 | 4.4 | 2.9 | 7.8 | 0.0 | 45.9 | 0.0 | 5.4 | 0.0 | 0.6 | 0.0 | 7.9 | 0.0 | 13.1 | 0.1 |
| *L. nigroviridis* | 2 | 26.2 | 35.7 | 5.3 | 0.0 | 0.1 | 22.4 | 0.0 | 0.0 | 1.3 | 0.0 | 4.0 | 0.0 | 0.0 | 3.7 | 1.3 |
| *L. nitidus* | 1 | 0.0 | 18.3 | 29.9 | 12.4 | 8.8 | 17.3 | 0.0 | 0.0 | 0.5 | 2.9 | 7.2 | 0.6 | 0.6 | 0.4 | 1.1 |
| *L. occipitalis* | 1 | 8.5 | 44.1 | 0.4 | 3.2 | 6.7 | 12.6 | 3.3 | 5.7 | 7.1 | 4.8 | 2.1 | 0.0 | 0.0 | 0.1 | 1.4 |
| *L. olongasta* | 1 | 1.3 | 1.9 | 20.4 | 3.4 | 6.9 | 14.2 | 33.2 | 0.6 | 0.1 | 11.3 | 2.5 | 1.4 | 1.2 | 0.2 | 1.4 |
| *L. orientalis* | 2 | 10.3 | 46.4 | 0.1 | 2.1 | 0.8 | 4.1 | 11.5 | 0.0 | 0.0 | 1.7 | 0.0 | 17.6 | 0.8 | 2.7 | 1.9 |
| *L. ornatus* | 2 | 10.1 | 37.7 | 0.0 | 6.0 | 0.0 | 28.3 | 8.8 | 1.6 | 4.5 | 0.4 | 0.1 | 0.0 | 0.3 | 0.4 | 1.8 |
| *L. pagaburoi* | 2 | 1.6 | 14.3 | 20.8 | 0.0 | 0.3 | 34.6 | 1.6 | 0.8 | 0.0 | 0.1 | 0.0 | 17.0 | 0.0 | 8.8 | 0.1 |
| *L. paulinae* | 2 | 5.4 | 46.1 | 2.1 | 0.1 | 3.2 | 0.4 | 3.2 | 1.0 | 0.0 | 37.1 | 0.0 | 0.0 | 0.0 | 0.4 | 1.0 |
| *L. petrophilus* | 2 | 0.2 | 20.4 | 11.2 | 13.0 | 2.8 | 20.9 | 0.0 | 0.0 | 17.8 | 4.1 | 0.4 | 0.0 | 0.3 | 8.9 | 0.0 |
| *L. pictus* | 2 | 6.9 | 2.5 | 2.6 | 6.6 | 9.8 | 2.8 | 9.1 | 3.0 | 3.0 | 1.2 | 16.6 | 3.6 | 1.1 | 0.9 | 30.3 |
| *L. platei* | 1 | 6.1 | 36.7 | 1.6 | 0.4 | 1.0 | 9.1 | 10.0 | 0.3 | 10.1 | 0.2 | 3.0 | 18.1 | 0.1 | 0.2 | 3.1 |
| *L. pseudoanomalus* | 1 | 18.1 | 0.1 | 0.0 | 46.0 | 0.0 | 6.2 | 0.0 | 0.1 | 0.0 | 0.0 | 0.3 | 0.0 | 12.2 | 0.6 | 16.4 |
| *L. pseudolemniscatus* | 1 | 22.1 | 13.8 | 4.6 | 1.7 | 1.9 | 10.6 | 31.0 | 1.4 | 0.0 | 1.8 | 5.1 | 0.0 | 0.0 | 6.0 | 0.0 |
| *L. puna* | 2 | 20.4 | 2.5 | 0.6 | 9.6 | 20.7 | 27.0 | 0.2 | 3.7 | 0.8 | 2.8 | 0.1 | 0.0 | 3.3 | 8.3 | 0.0 |
| *L. quilmes* | 1 | 0.4 | 19.2 | 2.4 | 6.4 | 2.6 | 32.1 | 5.9 | 0.4 | 0.2 | 9.0 | 0.3 | 0.4 | 0.0 | 7.2 | 13.5 |
| *L. ramirezae* | 1 | 2.7 | 0.1 | 23.4 | 0.5 | 4.2 | 25.0 | 0.9 | 0.0 | 30.9 | 7.6 | 0.4 | 0.0 | 1.2 | 1.7 | 1.4 |
| *L. riojanus* | 1 | 2.0 | 11.3 | 2.0 | 45.7 | 0.6 | 0.9 | 1.9 | 0.0 | 0.1 | 0.1 | 1.0 | 33.5 | 0.8 | 0.1 | 0.0 |
| *L. rosenmannii* | 2 | 0.1 | 0.0 | 2.3 | 27.5 | 19.1 | 6.7 | 27.2 | 0.0 | 0.0 | 0.2 | 0.3 | 7.9 | 0.6 | 0.4 | 7.7 |
| *L. rothi* | 1 | 0.2 | 10.9 | 28.2 | 7.7 | 12.7 | 25.2 | 0.0 | 0.1 | 0.0 | 0.1 | 3.3 | 4.4 | 3.1 | 3.7 | 0.4 |
| *L. ruibali* | 2 | 0.0 | 0.0 | 28.1 | 25.5 | 0.0 | 1.3 | 0.1 | 0.0 | 30.8 | 0.0 | 0.1 | 0.2 | 13.8 | 0.0 | 0.1 |
| *L. sarmientoi* | 2 | 10.5 | 3.2 | 9.2 | 13.0 | 7.4 | 5.2 | 1.3 | 0.7 | 15.2 | 13.1 | 6.9 | 0.0 | 11.0 | 0.4 | 2.9 |
| *L. scapularis* | 1 | 1.0 | 11.6 | 21.1 | 1.7 | 0.0 | 26.9 | 9.6 | 0.1 | 0.0 | 13.1 | 2.6 | 0.0 | 0.2 | 0.3 | 11.8 |
| *L. schroederi* | 2 | 0.1 | 1.8 | 36.1 | 5.2 | 0.8 | 0.2 | 0.0 | 24.3 | 2.7 | 0.1 | 0.0 | 4.1 | 24.3 | 0.3 | 0.0 |
| *L. scolaroi* | 2 | 7.3 | 18.6 | 9.9 | 19.4 | 7.3 | 0.0 | 1.9 | 0.0 | 12.1 | 0.0 | 0.0 | 4.0 | 19.2 | 0.0 | 0.3 |
| *L. silvanae* | 2 | 12.5 | 0.4 | 0.3 | 0.1 | 0.5 | 48.1 | 0.4 | 0.3 | 1.0 | 4.8 | 6.8 | 21.8 | 1.6 | 0.8 | 0.6 |
| *L. somuncurae* | 2 | 3.7 | 2.1 | 2.6 | 0.1 | 10.6 | 14.6 | 0.2 | 0.4 | 34.6 | 0.3 | 3.3 | 3.1 | 17.8 | 0.1 | 6.5 |
| *L. stolzmannii* | 2 | 12.5 | 3.4 | 36.2 | 5.7 | 3.3 | 0.4 | 0.0 | 0.0 | 0.1 | 7.4 | 0.3 | 2.1 | 27.1 | 0.9 | 0.6 |
| *L. tari* | 2 | 37.6 | 0.3 | 14.2 | 0.6 | 10.2 | 9.1 | 1.3 | 0.4 | 0.0 | 0.2 | 1.4 | 22.8 | 1.8 | 0.0 | 0.1 |
| *L. tenuis* | 1 | 4.1 | 1.7 | 5.6 | 16.7 | 11.1 | 25.0 | 5.6 | 1.5 | 5.6 | 1.9 | 1.9 | 1.7 | 4.8 | 10.2 | 2.6 |
| *L. torresi* | 2 | 0.0 | 4.6 | 31.2 | 15.1 | 0.5 | 0.4 | 0.1 | 0.1 | 0.0 | 7.7 | 10.8 | 0.0 | 3.7 | 0.0 | 25.8 |
| *L. tristis* | 2 | 0.0 | 0.6 | 15.1 | 48.3 | 0.0 | 0.1 | 0.0 | 0.0 | 9.4 | 0.3 | 2.7 | 2.6 | 17.5 | 3.4 | 0.0 |
| *L. uspallatensis* | 1 | 0.0 | 0.0 | 31.6 | 54.1 | 0.0 | 1.3 | 0.0 | 0.0 | 4.0 | 2.8 | 0.1 | 0.0 | 6.0 | 0.0 | 0.1 |
| *L. valdesianus* | 2 | 23.8 | 3.5 | 6.6 | 3.0 | 0.0 | 0.1 | 3.6 | 0.0 | 0.3 | 34.1 | 0.0 | 4.2 | 20.8 | 0.0 | 0.0 |
| *L. wiegmannii* | 1 | 6.3 | 4.0 | 3.6 | 35.5 | 4.8 | 2.7 | 4.8 | 12.5 | 4.1 | 3.8 | 9.6 | 0.6 | 0.8 | 5.2 | 1.7 |
| *L. xanthoviridis* | 1 | 0.0 | 10.0 | 2.2 | 0.0 | 32.5 | 1.4 | 0.0 | 0.0 | 29.9 | 1.4 | 12.5 | 10.1 | 0.0 | 0.0 | 0.0 |
| *L. zapallarensis* | 1 | 0.0 | 16.8 | 0.8 | 26.7 | 0.0 | 8.0 | 6.3 | 0.0 | 0.2 | 19.7 | 0.4 | 10.5 | 0.0 | 8.4 | 2.2 |
| *L. zullyiae* | 2 | 10.1 | 18.8 | 12.7 | 0.0 | 0.0 | 0.0 | 0.3 | 2.3 | 29.6 | 0.3 | 9.8 | 0.0 | 14.2 | 0.2 | 1.7 |
| *P. adrianae* | 2 | 0.0 | 30.4 | 17.3 | 0.0 | 0.0 | 2.4 | 0.0 | 0.0 | 7.9 | 0.0 | 0.0 | 0.0 | 0.0 | 13.8 | 28.2 |
| *P. agilis* | 2 | 3.3 | 17.1 | 0.0 | 24.2 | 0.0 | 0.0 | 26.3 | 1.9 | 1.9 | 12.3 | 2.8 | 9.4 | 0.7 | 0.0 | 0.1 |
| *P. ceii* | 2 | 0.0 | 6.6 | 19.2 | 20.4 | 0.0 | 25.7 | 0.0 | 0.0 | 26.1 | 0.0 | 0.0 | 0.0 | 0.0 | 0.0 | 2.0 |
| *P. excelsus* | 2 | 0.1 | 13.7 | 0.0 | 25.3 | 0.2 | 0.1 | 0.2 | 0.0 | 17.9 | 3.6 | 2.6 | 0.6 | 15.1 | 19.9 | 0.7 |
| *P. indistinctus* | 2 | 28.3 | 0.2 | 13.0 | 4.9 | 5.9 | 1.2 | 22.0 | 11.7 | 0.0 | 2.8 | 1.2 | 0.6 | 1.8 | 2.2 | 4.2 |
| *P. patagonicus* | 2 | 9.3 | 21.1 | 3.9 | 2.6 | 7.9 | 23.9 | 0.0 | 4.3 | 0.0 | 6.3 | 0.6 | 15.1 | 0.3 | 3.6 | 1.1 |
| *P. somuncurensis* | 2 | 5.7 | 0.0 | 18.3 | 3.8 | 0.3 | 26.9 | 0.0 | 0.9 | 10.3 | 0.2 | 3.3 | 0.0 | 20.1 | 0.0 | 10.2 |
| *P. spurcus* | 2 | 0.0 | 0.0 | 8.6 | 10.1 | 0.5 | 0.3 | 0.0 | 0.0 | 23.1 | 0.0 | 0.4 | 1.1 | 20.2 | 19.4 | 16.3 |
| *P. tenebrosus* | 2 | 4.9 | 1.2 | 18.7 | 4.6 | 0.0 | 1.2 | 3.1 | 7.9 | 17.9 | 1.5 | 17.2 | 0.3 | 19.4 | 0.4 | 1.7 |
| *P. vociferator* | 2 | 6.3 | 28.6 | 19.6 | 0.9 | 0.0 | 1.0 | 0.0 | 0.0 | 12.2 | 1.1 | 0.0 | 3.6 | 2.2 | 21.3 | 3.2 |
| *P. zapalensis* | 2 | 21.8 | 0.0 | 18.5 | 0.0 | 0.0 | 15.4 | 5.6 | 0.1 | 10.3 | 2.0 | 19.1 | 0.7 | 0.0 | 6.3 | 0.2 |
| *S. aculeatus* | 1 | 0.0 | 13.8 | 38.6 | 34.2 | 0.9 | 0.0 | 0.0 | 0.0 | 0.0 | 0.0 | 0.0 | 0.0 | 9.9 | 2.6 | 0.0 |
| *S. angel* | 1 | 16.9 | 0.0 | 13.0 | 0.0 | 26.8 | 0.4 | 4.3 | 9.4 | 0.5 | 2.6 | 21.3 | 0.7 | 0.7 | 3.2 | 0.2 |
| *S. angulifer* | 1 | 0.5 | 0.1 | 39.6 | 0.1 | 1.0 | 7.7 | 0.1 | 3.6 | 1.9 | 1.0 | 0.3 | 14.2 | 24.6 | 0.3 | 5.0 |
| *S. apurimacus* | 1 | 0.0 | 37.2 | 3.3 | 29.0 | 8.8 | 10.5 | 0.0 | 0.0 | 0.6 | 0.2 | 0.1 | 4.9 | 0.6 | 0.3 | 4.5 |
| *S. azureus* | 1 | 6.8 | 4.7 | 37.2 | 10.1 | 0.0 | 0.0 | 0.5 | 0.0 | 5.3 | 0.0 | 0.0 | 32.6 | 0.0 | 0.0 | 2.8 |
| *S. boettgeri* | 1 | 0.0 | 35.3 | 27.8 | 14.1 | 0.0 | 2.1 | 0.2 | 1.7 | 0.0 | 2.6 | 0.0 | 9.8 | 0.2 | 2.7 | 3.5 |
| *S. cadlei* | 1 | 18.6 | 1.0 | 4.7 | 27.3 | 2.7 | 25.0 | 0.5 | 0.9 | 0.6 | 0.0 | 1.3 | 10.7 | 0.4 | 1.6 | 4.7 |
| *S. caducus* | 1 | 22.4 | 0.0 | 1.7 | 27.2 | 0.0 | 0.2 | 0.0 | 17.8 | 0.0 | 2.5 | 1.3 | 18.1 | 0.0 | 1.0 | 7.8 |
| *S. chota* | 1 | 0.0 | 0.0 | 0.3 | 33.9 | 0.3 | 0.2 | 0.0 | 0.1 | 3.4 | 0.0 | 21.4 | 14.3 | 0.7 | 24.4 | 1.0 |
| *S. chrysopygus* | 1 | 0.1 | 0.0 | 10.8 | 21.5 | 0.0 | 42.3 | 1.5 | 7.5 | 0.7 | 0.1 | 0.0 | 6.6 | 1.0 | 2.4 | 5.5 |
| *S. doellojuradoi* | 1 | 0.0 | 0.2 | 10.3 | 0.0 | 0.0 | 0.1 | 0.0 | 41.6 | 0.0 | 0.0 | 3.0 | 9.1 | 3.9 | 1.3 | 30.5 |
| *S. dumerilii* | 1 | 0.8 | 1.1 | 13.8 | 21.6 | 0.3 | 13.6 | 2.2 | 0.0 | 4.0 | 0.4 | 32.6 | 0.0 | 0.6 | 8.6 | 0.4 |
| *S. empetrus* | 1 | 0.0 | 24.8 | 26.7 | 2.2 | 0.0 | 0.0 | 0.0 | 18.1 | 0.6 | 0.5 | 0.6 | 4.1 | 13.0 | 0.7 | 8.7 |
| *S. erythrogaster* | 1 | 2.1 | 33.4 | 0.3 | 9.4 | 0.0 | 0.0 | 0.0 | 0.5 | 5.2 | 2.3 | 0.0 | 20.2 | 1.4 | 19.2 | 6.0 |
| *S. festae* | 1 | 43.2 | 9.3 | 0.0 | 4.1 | 1.6 | 0.0 | 1.2 | 6.8 | 0.0 | 21.1 | 1.2 | 6.5 | 0.5 | 1.5 | 3.0 |
| *S. fimbriatus* | 1 | 0.0 | 18.9 | 0.2 | 21.5 | 1.9 | 0.0 | 12.2 | 0.0 | 1.4 | 6.4 | 6.8 | 22.1 | 0.0 | 3.0 | 5.6 |
| *S. guentheri* | 1 | 43.1 | 15.3 | 0.8 | 0.9 | 0.1 | 0.6 | 0.0 | 0.1 | 1.1 | 1.7 | 24.3 | 0.0 | 0.2 | 0.9 | 10.9 |
| *S. huancabambae* | 1 | 2.3 | 0.0 | 19.9 | 4.2 | 1.3 | 1.2 | 0.6 | 0.9 | 8.8 | 13.8 | 23.8 | 0.0 | 5.8 | 17.4 | 0.0 |
| *S. humeralis* | 1 | 4.3 | 5.1 | 0.4 | 38.4 | 0.1 | 1.2 | 8.1 | 10.7 | 0.1 | 7.9 | 0.0 | 0.0 | 3.3 | 20.3 | 0.1 |
| *S. imitator* | 1 | 0.0 | 14.3 | 42.9 | 0.0 | 0.0 | 2.7 | 0.0 | 0.0 | 0.0 | 2.7 | 0.9 | 33.3 | 0.0 | 3.2 | 0.0 |
| *S. iridescens* | 1 | 0.0 | 7.6 | 0.7 | 3.9 | 10.3 | 0.8 | 39.3 | 0.2 | 0.7 | 0.8 | 0.0 | 2.3 | 18.4 | 1.0 | 14.0 |
| *S. latebrosus* | 1 | 0.2 | 17.2 | 30.4 | 0.0 | 0.7 | 15.1 | 0.5 | 0.2 | 0.5 | 0.0 | 0.0 | 27.9 | 5.7 | 1.2 | 0.4 |
| *S. limitaris* | 1 | 0.0 | 0.0 | 39.4 | 0.8 | 0.0 | 0.1 | 0.0 | 0.0 | 1.4 | 0.1 | 0.0 | 34.2 | 18.4 | 3.4 | 2.2 |
| *S. marmoratus* | 1 | 0.0 | 33.4 | 0.0 | 10.3 | 0.0 | 0.0 | 0.0 | 0.0 | 1.4 | 0.0 | 0.0 | 0.0 | 23.0 | 1.8 | 30.1 |
| *S. melanopygus* | 1 | 0.3 | 24.9 | 27.6 | 5.6 | 0.3 | 25.7 | 0.0 | 0.5 | 0.6 | 0.0 | 0.0 | 0.4 | 5.9 | 1.2 | 7.0 |
| *S. modestus* | 1 | 0.0 | 0.0 | 32.9 | 36.1 | 1.3 | 0.0 | 19.7 | 0.0 | 3.7 | 6.2 | 0.0 | 0.0 | 0.0 | 0.0 | 0.1 |
| *S. ochoai* | 1 | 0.0 | 40.6 | 0.1 | 23.8 | 0.0 | 0.3 | 0.0 | 5.5 | 0.1 | 0.0 | 0.0 | 7.0 | 4.9 | 0.0 | 17.7 |
| *S. ornatissimus* | 1 | 0.8 | 2.7 | 9.6 | 0.2 | 16.2 | 0.4 | 13.3 | 9.5 | 0.4 | 1.2 | 0.1 | 4.2 | 15.9 | 0.0 | 25.5 |
| *S. ornatus* | 1 | 0.0 | 0.1 | 0.4 | 35.6 | 0.2 | 28.4 | 1.1 | 0.0 | 0.4 | 28.1 | 0.7 | 2.2 | 0.1 | 0.0 | 2.7 |
| *S. pectinatus* | 1 | 0.0 | 0.0 | 49.7 | 27.5 | 0.0 | 10.4 | 0.2 | 0.0 | 11.9 | 0.0 | 0.0 | 0.1 | 0.0 | 0.1 | 0.1 |
| *S. percultus* | 1 | 0.1 | 1.5 | 48.7 | 0.0 | 0.0 | 0.5 | 0.1 | 0.0 | 0.5 | 19.1 | 4.1 | 22.3 | 0.2 | 2.9 | 0.0 |
| *S. prionotus* | 1 | 1.6 | 23.0 | 19.8 | 20.5 | 7.8 | 0.0 | 0.1 | 0.2 | 0.1 | 0.0 | 1.8 | 5.3 | 10.5 | 1.8 | 7.5 |
| *S. puyango* | 1 | 5.1 | 0.7 | 12.5 | 0.0 | 0.4 | 5.9 | 0.0 | 1.4 | 2.9 | 0.0 | 0.0 | 42.4 | 18.6 | 0.0 | 10.1 |
| *S. rhodomelas* | 1 | 0.4 | 1.0 | 30.1 | 1.6 | 0.0 | 5.5 | 2.3 | 0.8 | 3.0 | 4.4 | 16.7 | 23.4 | 0.6 | 8.5 | 1.7 |
| *S. roseiventris* | 1 | 8.5 | 2.1 | 8.1 | 5.8 | 0.9 | 5.0 | 1.0 | 0.6 | 6.7 | 2.0 | 2.7 | 7.3 | 15.0 | 29.6 | 4.7 |
| *S. sinesaccus* | 1 | 0.1 | 0.0 | 0.0 | 15.4 | 8.1 | 0.0 | 0.0 | 0.1 | 0.0 | 4.4 | 0.0 | 39.4 | 2.9 | 13.4 | 16.2 |
| *S. torquatus* | 1 | 0.0 | 34.7 | 29.1 | 3.8 | 0.0 | 0.7 | 1.7 | 0.0 | 17.7 | 0.0 | 0.0 | 0.0 | 0.2 | 2.8 | 9.3 |
| *S. trachycephalus* | 1 | 29.3 | 0.0 | 4.9 | 1.8 | 0.7 | 29.0 | 0.0 | 0.0 | 2.8 | 0.0 | 0.3 | 0.3 | 8.4 | 17.9 | 4.6 |
| *S. variabilis* | 1 | 0.9 | 4.2 | 0.2 | 29.9 | 0.0 | 0.5 | 0.3 | 11.5 | 0.2 | 2.6 | 14.9 | 5.5 | 7.1 | 0.9 | 21.3 |
| *S. varius* | 1 | 0.0 | 0.0 | 3.7 | 0.3 | 0.9 | 0.1 | 0.0 | 17.5 | 11.5 | 7.9 | 2.3 | 5.0 | 8.9 | 4.2 | 37.7 |

**Table S2. Distributional changes between current and future SDMs using the GISS-ER model and considering the minimum scenario of greenhouse gases emissions (RCP 2.6). Reproductive mode (R), where (1=oviparous and 2=viviparous), Expansion areas (E), Areas with no changes (N) and contraction areas (C), Future geographic range (F) and Total range shifts (T).**

| **Species** | **R** | **Range (km^2^)** | **E (km^2^)** | **E %** | **N (km^2^)** | **N %** | **C (km^2^)** | **C %** | **F (km^2^)** | **ΔD (km^2^)** | **ΔT %** |
| --- | --- | --- | --- | --- | --- | --- | --- | --- | --- | --- | --- |
| *L. abaucan* | 1 | 90938.8 | 1307.3 | 1.4 | 78860.3 | 86.6 | 12182.9 | 13.4 | 80167.7 | -10875.6 | -11.9 |
| *L. albiceps* | 2 | 78919.6 | 10910.4 | 13.8 | 69411.9 | 88.0 | 9507.7 | 12.0 | 80322.2 | 1402.6 | 1.8 |
| *L. alticolor* | 2 | 208267.7 | 41069.9 | 19.7 | 203325.4 | 97.6 | 4942.4 | 2.4 | 244395.3 | 36127.5 | 17.3 |
| *L. andinus* | 2 | 205979.7 | 29293.7 | 14.2 | 192060.2 | 93.2 | 13919.5 | 6.8 | 221353.9 | 15374.1 | 7.5 |
| *L. anomalus* | 1 | 196109.6 | 17195.7 | 8.8 | 194201.1 | 99.0 | 1908.5 | 1.0 | 211396.9 | 15287.3 | 7.8 |
| *L. archeforus* | 2 | 17391.1 | 1903.5 | 10.9 | 16103.9 | 92.6 | 1287.2 | 7.4 | 18007.3 | 616.2 | 3.5 |
| *L. atacamensis* | 1 | 96535.8 | 4935.9 | 5.1 | 86408.1 | 89.4 | 10227.7 | 10.6 | 91343.9 | -5291.9 | -5.5 |
| *L. audituvelatus* | 2 | 101796.3 | 19240.0 | 18.9 | 94928.7 | 93.3 | 6867.5 | 6.7 | 114168.7 | 12372.5 | 12.2 |
| *L. austromendocinus* | 2 | 289262.7 | 27934.3 | 9.7 | 254574.9 | 88.0 | 34687.7 | 12.0 | 282509.2 | -6753.5 | -2.3 |
| *L. bellii* | 2 | 4047.9 | 0.0 | 0.0 | 3782.4 | 93.4 | 265.5 | 6.6 | 3782.4 | -265.5 | -6.6 |
| *L. bibronii* | 1 | 899817.2 | 68200.4 | 7.6 | 870133.8 | 96.7 | 29683.4 | 3.3 | 938334.2 | 38517.0 | 4.3 |
| *L. bitaeniatus* | 1 | 372936.1 | 30695.1 | 8.2 | 293760.4 | 78.8 | 79175.7 | 21.2 | 324455.5 | -48480.6 | -13.0 |
| *L. boulengeri* | 1 | 695035.0 | 14865.0 | 2.1 | 661628.3 | 95.2 | 33406.7 | 4.8 | 676493.3 | -18541.7 | -2.7 |
| *L. buergeri* | 2 | 107760.8 | 7834.1 | 7.3 | 91977.5 | 85.4 | 15783.3 | 14.6 | 99811.6 | -7949.2 | -7.4 |
| *L. canqueli* | 1 | 35544.7 | 7470.5 | 21.0 | 30456.4 | 85.7 | 5088.3 | 14.3 | 37926.9 | 2382.2 | 6.7 |
| *L. ceii* | 2 | 234795.8 | 76755.0 | 32.7 | 198729.9 | 84.6 | 36065.8 | 15.4 | 275485.0 | 40689.2 | 17.3 |
| *L. chacoensis* | 1 | 989974.1 | 34564.3 | 3.5 | 959459.0 | 96.9 | 30515.1 | 3.1 | 994023.3 | 4049.2 | 0.4 |
| *L. chaltin* | 1 | 50253.4 | 7330.4 | 14.6 | 49602.6 | 98.7 | 650.8 | 1.3 | 56933.0 | 6679.6 | 13.3 |
| *L. chiliensis* | 1 | 193882.0 | 2210.8 | 1.1 | 187132.5 | 96.5 | 6749.4 | 3.5 | 189343.3 | -4538.6 | -2.3 |
| *L. constanzae* | 1 | 42970.9 | 6625.1 | 15.4 | 39722.3 | 92.4 | 3248.6 | 7.6 | 46347.4 | 3376.5 | 7.9 |
| *L. curicensis* | 2 | 9188.0 | 797.0 | 8.7 | 8708.8 | 94.8 | 479.2 | 5.2 | 9505.8 | 317.8 | 3.5 |
| *L. curis* | 2 | 315.9 | 38.8 | 12.3 | 290.0 | 91.8 | 25.9 | 8.2 | 328.8 | 12.9 | 4.1 |
| *L. cuyanus* | 1 | 192038.8 | 9938.7 | 5.2 | 181427.5 | 94.5 | 10611.3 | 5.5 | 191366.1 | -672.7 | -0.4 |
| *L. cyanogaster* | 2 | 181410.0 | 4424.8 | 2.4 | 171024.0 | 94.3 | 10386.0 | 5.7 | 175448.7 | -5961.2 | -3.3 |
| *L. darwinii* | 1 | 1071745.3 | 67873.5 | 6.3 | 997980.6 | 93.1 | 73764.7 | 6.9 | 1065854.1 | -5891.2 | -0.5 |
| *L. elongatus* | 2 | 237344.2 | 20504.0 | 8.6 | 217831.2 | 91.8 | 19513.0 | 8.2 | 238335.2 | 991.0 | 0.4 |
| *L. escarchadosi* | 2 | 154111.2 | 7159.0 | 4.6 | 141019.5 | 91.5 | 13091.7 | 8.5 | 148178.6 | -5932.6 | -3.8 |
| *L. fabiani* | 2 | 43863.8 | 5884.6 | 13.4 | 43255.4 | 98.6 | 608.4 | 1.4 | 49140.0 | 5276.2 | 12.0 |
| *L. fitzgeraldi* | 2 | 26571.9 | 5107.5 | 19.2 | 23763.3 | 89.4 | 2808.5 | 10.6 | 28870.8 | 2298.9 | 8.7 |
| *L. fitzingerii* | 1 | 313633.5 | 9915.9 | 3.2 | 294213.1 | 93.8 | 19420.4 | 6.2 | 304129.1 | -9504.5 | -3.0 |
| *L. fuscus* | 1 | 21660.1 | 1878.8 | 8.7 | 19997.6 | 92.3 | 1662.5 | 7.7 | 21876.4 | 216.3 | 1.0 |
| *L. gallardoi* | 2 | 105484.2 | 22475.6 | 21.3 | 97692.7 | 92.6 | 7791.5 | 7.4 | 120168.3 | 14684.1 | 13.9 |
| *L. goetschi* | 1 | 286784.8 | 31936.0 | 11.1 | 256881.7 | 89.6 | 29903.1 | 10.4 | 288817.7 | 2032.9 | 0.7 |
| *L. gracilis* | 1 | 747145.0 | 27258.5 | 3.6 | 674555.4 | 90.3 | 72589.6 | 9.7 | 701814.0 | -45331.0 | -6.1 |
| *L. gravenhorsti* | 2 | 5030.3 | 464.1 | 9.2 | 4502.3 | 89.5 | 528.0 | 10.5 | 4966.4 | -64.0 | -1.3 |
| *L. hatcheri* | 2 | 53393.0 | 3986.2 | 7.5 | 44278.7 | 82.9 | 9114.3 | 17.1 | 48264.8 | -5128.2 | -9.6 |
| *L. irregularis* | 2 | 157989.8 | 9564.7 | 6.1 | 140131.0 | 88.7 | 17858.8 | 11.3 | 149695.7 | -8294.1 | -5.2 |
| *L. isabelae* | 2 | 15416.4 | 1486.8 | 9.6 | 13632.6 | 88.4 | 1783.8 | 11.6 | 15119.4 | -297.0 | -1.9 |
| *L. jamesi* | 2 | 52157.7 | 3494.3 | 6.7 | 48726.4 | 93.4 | 3431.3 | 6.6 | 52220.7 | 63.0 | 0.1 |
| *L. kingii* | 2 | 256542.5 | 33935.8 | 13.2 | 243115.3 | 94.8 | 13427.2 | 5.2 | 277051.1 | 20508.6 | 8.0 |
| *L. koslowskyi* | 1 | 224339.8 | 14599.0 | 6.5 | 195778.8 | 87.3 | 28561.0 | 12.7 | 210377.8 | -13962.0 | -6.2 |
| *L. kriegi* | 2 | 240999.3 | 13869.9 | 5.8 | 217897.9 | 90.4 | 23101.4 | 9.6 | 231767.8 | -9231.5 | -3.8 |
| *L. lavillai* | 2 | 83802.1 | 25785.9 | 30.8 | 71409.5 | 85.2 | 12392.6 | 14.8 | 97195.3 | 13393.3 | 16.0 |
| *L. lemniscatus* | 1 | 120069.2 | 6525.6 | 5.4 | 118000.1 | 98.3 | 2069.1 | 1.7 | 124525.8 | 4456.5 | 3.7 |
| *L. leopardinus* | 2 | 1802.8 | 49.0 | 2.7 | 1565.9 | 86.9 | 236.9 | 13.1 | 1615.0 | -187.9 | -10.4 |
| *L. lineomaculatus* | 2 | 542059.2 | 15863.9 | 2.9 | 535950.6 | 98.9 | 6108.7 | 1.1 | 551814.4 | 9755.2 | 1.8 |
| *L. lutzae* | 1 | 22466.6 | 1559.2 | 6.9 | 19098.5 | 85.0 | 3368.2 | 15.0 | 20657.6 | -1809.0 | -8.1 |
| *L. magellanicus* | 2 | 168930.4 | 2124.3 | 1.3 | 167261.8 | 99.0 | 1668.6 | 1.0 | 169386.0 | 455.7 | 0.3 |
| *L. manueli* | 2 | 19445.0 | 4154.0 | 21.4 | 15260.3 | 78.5 | 4184.7 | 21.5 | 19414.3 | -30.7 | -0.2 |
| *L. melanops* | 1 | 144530.5 | 21188.1 | 14.7 | 132705.9 | 91.8 | 11824.6 | 8.2 | 153894.0 | 9363.5 | 6.5 |
| *L. monticola* | 1 | 27566.9 | 1781.9 | 6.5 | 25273.6 | 91.7 | 2293.3 | 8.3 | 27055.5 | -511.3 | -1.9 |
| *L. multicolor* | 2 | 137461.2 | 18534.2 | 13.5 | 131351.0 | 95.6 | 6110.2 | 4.4 | 149885.2 | 12424.0 | 9.0 |
| *L. multimaculatus* | 1 | 103333.1 | 11449.0 | 11.1 | 92951.3 | 90.0 | 10381.8 | 10.0 | 104400.3 | 1067.2 | 1.0 |
| *L. nigriceps* | 2 | 12261.7 | 853.4 | 7.0 | 9751.9 | 79.5 | 2509.8 | 20.5 | 10605.3 | -1656.4 | -13.5 |
| *L. nigromaculatus* | 1 | 24503.1 | 2712.0 | 11.1 | 23467.3 | 95.8 | 1035.8 | 4.2 | 26179.3 | 1676.2 | 6.8 |
| *L. nigroviridis* | 2 | 7880.1 | 467.9 | 5.9 | 7703.4 | 97.8 | 176.7 | 2.2 | 8171.3 | 291.2 | 3.7 |
| *L. nitidus* | 1 | 114813.6 | 8706.3 | 7.6 | 108771.5 | 94.7 | 6042.1 | 5.3 | 117477.8 | 2664.3 | 2.3 |
| *L. occipitalis* | 1 | 76655.6 | 3367.1 | 4.4 | 72611.2 | 94.7 | 4044.3 | 5.3 | 75978.3 | -677.3 | -0.9 |
| *L. olongasta* | 1 | 106413.4 | 12716.8 | 12.0 | 102326.1 | 96.2 | 4087.3 | 3.8 | 115042.8 | 8629.4 | 8.1 |
| *L. orientalis* | 2 | 103472.1 | 16962.4 | 16.4 | 98341.6 | 95.0 | 5130.5 | 5.0 | 115303.9 | 11831.8 | 11.4 |
| *L. ornatus* | 2 | 254989.9 | 20016.2 | 7.8 | 253666.8 | 99.5 | 1323.1 | 0.5 | 273683.0 | 18693.1 | 7.3 |
| *L. pagaburoi* | 2 | 82030.4 | 20468.6 | 25.0 | 78311.9 | 95.5 | 3718.5 | 4.5 | 98780.5 | 16750.1 | 20.4 |
| *L. paulinae* | 2 | 883.6 | 63.7 | 7.2 | 848.2 | 96.0 | 35.4 | 4.0 | 911.9 | 28.3 | 3.2 |
| *L. petrophilus* | 2 | 366785.6 | 37526.9 | 10.2 | 320046.6 | 87.3 | 46739.0 | 12.7 | 357573.5 | -9212.1 | -2.5 |
| *L. pictus* | 2 | 238990.5 | 1731.0 | 0.7 | 219428.3 | 91.8 | 19562.2 | 8.2 | 221159.3 | -17831.2 | -7.5 |
| *L. platei* | 1 | 282900.0 | 38069.6 | 13.5 | 257683.3 | 91.1 | 25216.7 | 8.9 | 295752.9 | 12852.9 | 4.5 |
| *L. pseudoanomalus* | 1 | 184196.2 | 5077.3 | 2.8 | 161416.1 | 87.6 | 22780.1 | 12.4 | 166493.4 | -17702.8 | -9.6 |
| *L. pseudolemniscatus* | 1 | 43478.8 | 2830.5 | 6.5 | 40823.9 | 93.9 | 2654.9 | 6.1 | 43654.4 | 175.7 | 0.4 |
| *L. puna* | 2 | 243110.3 | 9195.5 | 3.8 | 207202.5 | 85.2 | 35907.8 | 14.8 | 216398.0 | -26712.3 | -11.0 |
| *L. quilmes* | 1 | 43780.8 | 3739.0 | 8.5 | 41204.9 | 94.1 | 2575.9 | 5.9 | 44943.9 | 1163.0 | 2.7 |
| *L. ramirezae* | 1 | 373872.6 | 17013.0 | 4.6 | 329280.1 | 88.1 | 44592.5 | 11.9 | 346293.0 | -27579.5 | -7.4 |
| *L. riojanus* | 1 | 120514.1 | 7830.8 | 6.5 | 110477.8 | 91.7 | 10036.3 | 8.3 | 118308.6 | -2205.5 | -1.8 |
| *L. rosenmannii* | 2 | 36956.0 | 6976.5 | 18.9 | 30557.2 | 82.7 | 6398.8 | 17.3 | 37533.8 | 577.8 | 1.6 |
| *L. rothi* | 1 | 266230.7 | 7644.0 | 2.9 | 232638.6 | 87.4 | 33592.1 | 12.6 | 240282.5 | -25948.2 | -9.7 |
| *L. ruibali* | 2 | 50178.9 | 7873.3 | 15.7 | 45938.7 | 91.5 | 4240.2 | 8.5 | 53812.0 | 3633.1 | 7.2 |
| *L. sarmientoi* | 2 | 177723.2 | 17248.1 | 9.7 | 171067.4 | 96.3 | 6655.8 | 3.7 | 188315.6 | 10592.3 | 6.0 |
| *L. scapularis* | 1 | 99423.3 | 4855.1 | 4.9 | 89882.0 | 90.4 | 9541.4 | 9.6 | 94737.1 | -4686.2 | -4.7 |
| *L. schroederi* | 2 | 269970.7 | 21266.1 | 7.9 | 256879.7 | 95.2 | 13090.9 | 4.8 | 278145.8 | 8175.2 | 3.0 |
| *L. scolaroi* | 2 | 12587.4 | 6485.2 | 51.5 | 11222.0 | 89.2 | 1365.4 | 10.8 | 17707.1 | 5119.7 | 40.7 |
| *L. silvanae* | 2 | 3462.8 | 121.5 | 3.5 | 3157.7 | 91.2 | 305.1 | 8.8 | 3279.2 | -183.6 | -5.3 |
| *L. somuncurae* | 2 | 25980.0 | 784.6 | 3.0 | 23526.3 | 90.6 | 2453.7 | 9.4 | 24311.0 | -1669.0 | -6.4 |
| *L. stolzmannii* | 2 | 21613.8 | 0.0 | 0.0 | 21200.7 | 98.1 | 413.0 | 1.9 | 21200.7 | -413.0 | -1.9 |
| *L. tari* | 2 | 53014.0 | 9200.2 | 17.4 | 47919.0 | 90.4 | 5095.1 | 9.6 | 57119.1 | 4105.1 | 7.7 |
| *L. tenuis* | 1 | 200967.7 | 1833.7 | 0.9 | 196751.2 | 97.9 | 4216.5 | 2.1 | 198584.9 | -2382.8 | -1.2 |
| *L. torresi* | 2 | 89738.9 | 6156.5 | 6.9 | 88723.5 | 98.9 | 1015.3 | 1.1 | 94880.0 | 5141.1 | 5.7 |
| *L. tristis* | 2 | 32362.3 | 1950.1 | 6.0 | 27443.6 | 84.8 | 4918.7 | 15.2 | 29393.7 | -2968.6 | -9.2 |
| *L. uspallatensis* | 1 | 21614.3 | 1825.8 | 8.4 | 21572.1 | 99.8 | 42.2 | 0.2 | 23397.9 | 1783.6 | 8.3 |
| *L. valdesianus* | 2 | 2201.2 | 234.2 | 10.6 | 2038.5 | 92.6 | 162.6 | 7.4 | 2272.7 | 71.6 | 3.3 |
| *L. wiegmannii* | 1 | 1437268.9 | 133186.5 | 9.3 | 1325302.5 | 92.2 | 111966.5 | 7.8 | 1458489.0 | 21220.1 | 1.5 |
| *L. xanthoviridis* | 1 | 56992.6 | 4970.0 | 8.7 | 46776.3 | 82.1 | 10216.3 | 17.9 | 51746.3 | -5246.3 | -9.2 |
| *L. zapallarensis* | 1 | 57175.1 | 7144.9 | 12.5 | 56853.2 | 99.4 | 321.9 | 0.6 | 63998.1 | 6823.0 | 11.9 |
| *L. zullyiae* | 2 | 6898.7 | 2470.4 | 35.8 | 6378.3 | 92.5 | 520.4 | 7.5 | 8848.7 | 1950.0 | 28.3 |
| *P. adrianae* | 2 | 36224.6 | 4994.1 | 13.8 | 32062.2 | 88.5 | 4162.4 | 11.5 | 37056.3 | 831.6 | 2.3 |
| *P. agilis* | 2 | 14139.9 | 6515.6 | 46.1 | 10189.1 | 72.1 | 3950.8 | 27.9 | 16704.7 | 2564.8 | 18.1 |
| *P. ceii* | 2 | 69306.8 | 6068.3 | 8.8 | 64178.0 | 92.6 | 5128.8 | 7.4 | 70246.3 | 939.5 | 1.4 |
| *P. excelsus* | 2 | 13996.4 | 1134.2 | 8.1 | 11061.7 | 79.0 | 2934.7 | 21.0 | 12195.8 | -1800.6 | -12.9 |
| *P. indistinctus* | 2 | 36252.0 | 5188.5 | 14.3 | 27354.9 | 75.5 | 8897.1 | 24.5 | 32543.4 | -3708.5 | -10.2 |
| *P. patagonicus* | 2 | 82070.1 | 8969.1 | 10.9 | 57471.2 | 70.0 | 24598.8 | 30.0 | 66440.3 | -15629.7 | -19.0 |
| *P. somuncurensis* | 2 | 32973.1 | 2973.1 | 9.0 | 25478.1 | 77.3 | 7495.0 | 22.7 | 28451.2 | -4521.9 | -13.7 |
| *P. spurcus* | 2 | 31644.2 | 2877.0 | 9.1 | 30042.7 | 94.9 | 1601.6 | 5.1 | 32919.7 | 1275.5 | 4.0 |
| *P. tenebrosus* | 2 | 20439.2 | 970.3 | 4.7 | 18836.4 | 92.2 | 1602.8 | 7.8 | 19806.7 | -632.5 | -3.1 |
| *P. vociferator* | 2 | 62428.5 | 2076.7 | 3.3 | 58870.7 | 94.3 | 3557.8 | 5.7 | 60947.4 | -1481.1 | -2.4 |
| *P. zapalensis* | 2 | 50382.5 | 8545.2 | 17.0 | 46501.2 | 92.3 | 3881.3 | 7.7 | 55046.4 | 4663.9 | 9.3 |
| *S. aculeatus* | 1 | 135137.3 | 26707.3 | 19.8 | 98022.5 | 72.5 | 37114.8 | 27.5 | 124729.8 | -10407.4 | -7.7 |
| *S. angel* | 1 | 24984.5 | 2923.6 | 11.7 | 22492.1 | 90.0 | 2492.1 | 10.0 | 25415.8 | 431.5 | 1.7 |
| *S. angulifer* | 1 | 96413.9 | 8904.8 | 9.2 | 77740.2 | 80.6 | 18673.7 | 19.4 | 86645.0 | -9768.9 | -10.1 |
| *S. apurimacus* | 1 | 203933.0 | 7249.4 | 3.6 | 179881.6 | 88.2 | 24051.4 | 11.8 | 187131.0 | -16802.0 | -8.2 |
| *S. azureus* | 1 | 1598474.4 | 32204.1 | 2.0 | 1429654.1 | 89.4 | 168820.3 | 10.6 | 1461858.2 | -136616.3 | -8.5 |
| *S. boettgeri* | 1 | 490376.8 | 134163.7 | 27.4 | 347580.7 | 70.9 | 142796.1 | 29.1 | 481744.4 | -8632.4 | -1.8 |
| *S. cadlei* | 1 | 86726.7 | 9875.6 | 11.4 | 74548.7 | 86.0 | 12177.9 | 14.0 | 84424.3 | -2302.3 | -2.7 |
| *S. caducus* | 1 | 2926552.5 | 255329.8 | 8.7 | 2627290.0 | 89.8 | 299262.4 | 10.2 | 2882619.8 | -43932.6 | -1.5 |
| *S. chota* | 1 | 18412.1 | 497.2 | 2.7 | 16295.9 | 88.5 | 2116.2 | 11.5 | 16793.1 | -1619.0 | -8.8 |
| *S. chrysopygus* | 1 | 57412.4 | 9935.9 | 17.3 | 54497.0 | 94.9 | 2915.4 | 5.1 | 64432.9 | 7020.5 | 12.2 |
| *S. doellojuradoi* | 1 | 430655.1 | 36685.1 | 8.5 | 396843.5 | 92.1 | 33811.6 | 7.9 | 433528.6 | 2873.5 | 0.7 |
| *S. dumerilii* | 1 | 459200.6 | 159239.7 | 34.7 | 279514.1 | 60.9 | 179686.5 | 39.1 | 438753.8 | -20446.8 | -4.5 |
| *S. empetrus* | 1 | 62092.2 | 10205.4 | 16.4 | 47022.4 | 75.7 | 15069.9 | 24.3 | 57227.8 | -4864.5 | -7.8 |
| *S. erythrogaster* | 1 | 309644.3 | 39541.7 | 12.8 | 285945.8 | 92.3 | 23698.5 | 7.7 | 325487.6 | 15843.3 | 5.1 |
| *S. festae* | 1 | 106181.0 | 15673.8 | 14.8 | 99478.3 | 93.7 | 6702.7 | 6.3 | 115152.1 | 8971.1 | 8.4 |
| *S. fimbriatus* | 1 | 4324355.6 | 540545.0 | 12.5 | 3835858.4 | 88.7 | 488497.2 | 11.3 | 4376403.3 | 52047.8 | 1.2 |
| *S. guentheri* | 1 | 32453.0 | 5740.1 | 17.7 | 29192.8 | 90.0 | 3260.2 | 10.0 | 34932.9 | 2479.9 | 7.6 |
| *S. huancabambae* | 1 | 53239.7 | 12676.2 | 23.8 | 46629.1 | 87.6 | 6610.6 | 12.4 | 59305.3 | 6065.6 | 11.4 |
| *S. humeralis* | 1 | 70709.2 | 7414.2 | 10.5 | 69451.9 | 98.2 | 1257.3 | 1.8 | 76866.1 | 6156.9 | 8.7 |
| *S. imitator* | 1 | 37303.0 | 4061.2 | 10.9 | 29179.1 | 78.2 | 8123.9 | 21.8 | 33240.3 | -4062.7 | -10.9 |
| *S. iridescens* | 1 | 215831.3 | 9293.7 | 4.3 | 199223.9 | 92.3 | 16607.4 | 7.7 | 208517.6 | -7313.7 | -3.4 |
| *S. latebrosus* | 1 | 200361.3 | 24294.6 | 12.1 | 164065.7 | 81.9 | 36295.5 | 18.1 | 188360.3 | -12000.9 | -6.0 |
| *S. limitaris* | 1 | 120280.1 | 15879.4 | 13.2 | 118556.5 | 98.6 | 1723.6 | 1.4 | 134435.9 | 14155.8 | 11.8 |
| *S. marmoratus* | 1 | 1372098.2 | 41214.4 | 3.0 | 1236573.5 | 90.1 | 135524.7 | 9.9 | 1277787.9 | -94310.3 | -6.9 |
| *S. melanopygus* | 1 | 51707.8 | 3562.4 | 6.9 | 43868.6 | 84.8 | 7839.3 | 15.2 | 47431.0 | -4276.8 | -8.3 |
| *S. modestus* | 1 | 177363.2 | 1747.5 | 1.0 | 177334.8 | 100.0 | 28.4 | 0.0 | 179082.3 | 1719.1 | 1.0 |
| *S. ochoai* | 1 | 576841.2 | 14008.8 | 2.4 | 550495.6 | 95.4 | 26345.6 | 4.6 | 564504.4 | -12336.7 | -2.1 |
| *S. ornatissimus* | 1 | 342026.6 | 41975.3 | 12.3 | 326594.0 | 95.5 | 15432.6 | 4.5 | 368569.3 | 26542.7 | 7.8 |
| *S. ornatus* | 1 | 190656.9 | 12916.2 | 6.8 | 187572.4 | 98.4 | 3084.5 | 1.6 | 200488.6 | 9831.6 | 5.2 |
| *S. pectinatus* | 1 | 1434841.3 | 26505.4 | 1.8 | 1330463.7 | 92.7 | 104377.6 | 7.3 | 1356969.2 | -77872.2 | -5.4 |
| *S. percultus* | 1 | 492878.3 | 6541.0 | 1.3 | 476104.8 | 96.6 | 16773.6 | 3.4 | 482645.8 | -10232.5 | -2.1 |
| *S. prionotus* | 1 | 5660188.9 | 1146709.2 | 20.3 | 5303909.6 | 93.7 | 356279.3 | 6.3 | 6450618.8 | 790429.9 | 14.0 |
| *S. puyango* | 1 | 119294.6 | 7572.3 | 6.3 | 112350.5 | 94.2 | 6944.1 | 5.8 | 119922.8 | 628.2 | 0.5 |
| *S. rhodomelas* | 1 | 29200.4 | 7353.0 | 25.2 | 26844.4 | 91.9 | 2356.1 | 8.1 | 34197.3 | 4996.9 | 17.1 |
| *S. roseiventris* | 1 | 9545380.6 | 1009959.8 | 10.6 | 8153175.3 | 85.4 | 1392205.3 | 14.6 | 9163135.1 | -382245.5 | -4.0 |
| *S. sinesaccus* | 1 | 3911739.1 | 436832.1 | 11.2 | 3908320.3 | 99.9 | 3418.7 | 0.1 | 4345152.4 | 433413.4 | 11.1 |
| *S. torquatus* | 1 | 225261.1 | 3069.7 | 1.4 | 205718.4 | 91.3 | 19542.7 | 8.7 | 208788.0 | -16473.1 | -7.3 |
| *S. trachycephalus* | 1 | 165230.9 | 12423.9 | 7.5 | 146383.0 | 88.6 | 18847.9 | 11.4 | 158807.0 | -6424.0 | -3.9 |
| *S. variabilis* | 1 | 173830.2 | 23571.7 | 13.6 | 165619.6 | 95.3 | 8210.6 | 4.7 | 189191.3 | 15361.1 | 8.8 |
| *S. varius* | 1 | 72428.8 | 5762.1 | 8.0 | 60451.1 | 83.5 | 11977.8 | 16.5 | 66213.2 | -6215.7 | -8.6 |

**Table S3. Distributional changes between current and future SDMs using the GISS-ER model and considering the maximum scenario of greenhouse gases emissions (RCP 8.5). Reproductive mode (R), where (1=oviparous and 2=viviparous), Expansion areas (E), Areas with no changes (N) and contraction areas (C), Future geographic range (F) and Total range shifts (T).**

| **Species** | **R** | **Range (km^2^)** | **E (km^2^)** | **E %** | **N (km^2^)** | **N %** | **C (km^2^)** | **C %** | **F (km^2^)** | **ΔD (km^2^)** | **ΔT %** |
| --- | --- | --- | --- | --- | --- | --- | --- | --- | --- | --- | --- |
| *L. abaucan* | 1 | 90938.8 | 1446.7 | 1.6 | 74560.6 | 81.9 | 16378.2 | 18.0 | 76007.3 | -14931.5 | -16.4 |
| *L. albiceps* | 2 | 78919.6 | 24883.2 | 31.5 | 63210.8 | 80.1 | 15708.8 | 19.9 | 88094.0 | 9174.4 | 11.6 |
| *L. alticolor* | 2 | 208267.7 | 17330.2 | 8.3 | 182390.6 | 87.6 | 25877.1 | 12.4 | 199720.8 | -8546.9 | -4.1 |
| *L. andinus* | 2 | 205979.7 | 40722.2 | 19.8 | 172260.3 | 83.6 | 33719.4 | 16.4 | 212982.5 | 7002.7 | 3.4 |
| *L. anomalus* | 1 | 196109.6 | 20680.9 | 10.5 | 186066.1 | 94.9 | 10043.5 | 5.1 | 206747.0 | 10637.4 | 5.4 |
| *L. archeforus* | 2 | 17391.1 | 3837.4 | 22.1 | 16554.4 | 95.2 | 836.7 | 4.8 | 20391.8 | 3000.8 | 17.3 |
| *L. atacamensis* | 1 | 96535.8 | 5777.5 | 6.0 | 80196.0 | 83.0 | 16339.8 | 16.9 | 85973.5 | -10562.3 | -10.9 |
| *L. audituvelatus* | 2 | 101796.3 | 23319.7 | 22.9 | 92573.3 | 90.9 | 9223.0 | 9.1 | 115893.0 | 14096.7 | 13.8 |
| *L. austromendocinus* | 2 | 289262.7 | 44676.0 | 15.4 | 241502.5 | 83.5 | 47760.2 | 16.5 | 286178.6 | -3084.1 | -1.1 |
| *L. bellii* | 2 | 4047.9 | 188.5 | 4.7 | 2870.8 | 70.9 | 1177.1 | 29.1 | 3059.3 | -988.6 | -24.4 |
| *L. bibronii* | 1 | 899817.2 | 142695.7 | 15.9 | 855048.4 | 95.0 | 44768.9 | 5.0 | 997744.0 | 97926.8 | 10.9 |
| *L. bitaeniatus* | 1 | 372936.1 | 57918.5 | 15.5 | 251921.4 | 67.6 | 121014.7 | 32.4 | 309839.9 | -63096.2 | -16.9 |
| *L. boulengeri* | 1 | 695035.0 | 52496.7 | 7.6 | 661044.5 | 95.1 | 33990.6 | 4.9 | 713541.1 | 18506.1 | 2.7 |
| *L. buergeri* | 2 | 107760.8 | 20755.0 | 19.3 | 99931.6 | 92.7 | 7829.2 | 7.3 | 120686.6 | 12925.8 | 12.0 |
| *L. canqueli* | 1 | 35544.7 | 8078.3 | 22.7 | 30951.9 | 87.1 | 4592.8 | 12.9 | 39030.2 | 3485.5 | 9.8 |
| *L. ceii* | 2 | 234795.8 | 141562.3 | 60.3 | 194407.5 | 82.8 | 40388.2 | 17.2 | 335969.8 | 101174.1 | 43.1 |
| *L. chacoensis* | 1 | 989974.1 | 25383.9 | 2.6 | 927632.1 | 93.7 | 62341.9 | 6.3 | 953016.0 | -36958.1 | -3.7 |
| *L. chaltin* | 1 | 50253.4 | 8696.7 | 17.3 | 49455.2 | 98.4 | 798.2 | 1.6 | 58151.9 | 7898.5 | 15.7 |
| *L. chiliensis* | 1 | 193882.0 | 8184.2 | 4.2 | 186342.3 | 96.1 | 7539.7 | 3.9 | 194526.5 | 644.5 | 0.3 |
| *L. constanzae* | 1 | 42970.9 | 7236.3 | 16.8 | 40816.8 | 95.0 | 2154.1 | 5.0 | 48053.0 | 5082.1 | 11.8 |
| *L. curicensis* | 2 | 9188.0 | 2800.1 | 30.5 | 8680.4 | 94.5 | 507.7 | 5.5 | 11480.4 | 2292.4 | 24.9 |
| *L. curis* | 2 | 315.9 | 201.2 | 63.7 | 274.7 | 87.0 | 41.2 | 13.0 | 475.8 | 160.0 | 50.6 |
| *L. cuyanus* | 1 | 192038.8 | 26573.2 | 13.8 | 173618.8 | 90.4 | 18420.0 | 9.6 | 200191.9 | 8153.2 | 4.2 |
| *L. cyanogaster* | 2 | 181410.0 | 7528.5 | 4.2 | 171745.0 | 94.7 | 9664.9 | 5.3 | 179273.6 | -2136.4 | -1.2 |
| *L. darwinii* | 1 | 1071745.3 | 111469.7 | 10.4 | 974396.6 | 90.9 | 97348.6 | 9.1 | 1085866.4 | 14121.1 | 1.3 |
| *L. elongatus* | 2 | 237344.2 | 59824.0 | 25.2 | 184025.6 | 77.5 | 53318.7 | 22.5 | 243849.6 | 6505.4 | 2.7 |
| *L. escarchadosi* | 2 | 154111.2 | 12834.5 | 8.3 | 130306.7 | 84.6 | 23804.5 | 15.4 | 143141.2 | -10970.0 | -7.1 |
| *L. fabiani* | 2 | 43863.8 | 6830.8 | 15.6 | 39938.9 | 91.1 | 3924.9 | 8.9 | 46769.7 | 2905.9 | 6.6 |
| *L. fitzgeraldi* | 2 | 26571.9 | 6986.1 | 26.3 | 24280.6 | 91.4 | 2291.3 | 8.6 | 31266.7 | 4694.8 | 17.7 |
| *L. fitzingerii* | 1 | 313633.5 | 31234.3 | 10.0 | 305616.9 | 97.4 | 8016.6 | 2.6 | 336851.2 | 23217.7 | 7.4 |
| *L. fuscus* | 1 | 21660.1 | 2117.4 | 9.8 | 18064.2 | 83.4 | 3595.9 | 16.6 | 20181.6 | -1478.5 | -6.8 |
| *L. gallardoi* | 2 | 105484.2 | 30055.0 | 28.5 | 87276.5 | 82.7 | 18207.7 | 17.3 | 117331.5 | 11847.2 | 11.2 |
| *L. goetschi* | 1 | 286784.8 | 30044.9 | 10.5 | 240735.7 | 83.9 | 46049.1 | 16.1 | 270780.6 | -16004.2 | -5.6 |
| *L. gracilis* | 1 | 747145.0 | 64191.4 | 8.6 | 636412.3 | 85.2 | 110732.8 | 14.8 | 700603.7 | -46541.3 | -6.2 |
| *L. gravenhorsti* | 2 | 5030.3 | 726.5 | 14.4 | 4529.3 | 90.0 | 501.0 | 10.0 | 5255.8 | 225.5 | 4.5 |
| *L. hatcheri* | 2 | 53393.0 | 12797.1 | 24.0 | 37677.5 | 70.6 | 15715.5 | 29.4 | 50474.7 | -2918.3 | -5.5 |
| *L. irregularis* | 2 | 157989.8 | 9627.1 | 6.1 | 124684.1 | 78.9 | 33305.8 | 21.1 | 134311.2 | -23678.6 | -15.0 |
| *L. isabelae* | 2 | 15416.4 | 3396.7 | 22.0 | 13324.4 | 86.4 | 2092.1 | 13.6 | 16721.1 | 1304.6 | 8.5 |
| *L. jamesi* | 2 | 52157.7 | 5995.5 | 11.5 | 44898.7 | 86.1 | 7259.1 | 13.9 | 50894.2 | -1263.6 | -2.4 |
| *L. kingii* | 2 | 256542.5 | 32555.2 | 12.7 | 237001.1 | 92.4 | 19541.3 | 7.6 | 269556.4 | 13013.9 | 5.1 |
| *L. koslowskyi* | 1 | 224339.8 | 27465.9 | 12.2 | 170433.1 | 76.0 | 53906.7 | 24.0 | 197899.0 | -26440.8 | -11.8 |
| *L. kriegi* | 2 | 240999.3 | 544.5 | 0.2 | 105384.0 | 43.7 | 135615.3 | 56.3 | 105928.5 | -135070.8 | -56.0 |
| *L. lavillai* | 2 | 83802.1 | 68350.1 | 81.6 | 54765.7 | 65.4 | 29036.4 | 34.6 | 123115.8 | 39313.7 | 46.9 |
| *L. lemniscatus* | 1 | 120069.2 | 6443.1 | 5.4 | 110504.8 | 92.0 | 9564.4 | 8.0 | 116947.9 | -3121.3 | -2.6 |
| *L. leopardinus* | 2 | 1802.8 | 125.8 | 7.0 | 1307.0 | 72.5 | 495.8 | 27.5 | 1432.8 | -370.0 | -20.5 |
| *L. lineomaculatus* | 2 | 542059.2 | 32385.2 | 6.0 | 523012.4 | 96.5 | 19046.9 | 3.5 | 555397.5 | 13338.3 | 2.5 |
| *L. lutzae* | 1 | 22466.6 | 1007.3 | 4.5 | 17380.5 | 77.4 | 5086.1 | 22.6 | 18387.8 | -4078.8 | -18.2 |
| *L. magellanicus* | 2 | 168930.4 | 2001.3 | 1.2 | 163210.8 | 96.6 | 5719.6 | 3.4 | 165212.1 | -3718.3 | -2.2 |
| *L. manueli* | 2 | 19445.0 | 4146.6 | 21.3 | 9860.2 | 50.7 | 9584.8 | 49.3 | 14006.8 | -5438.2 | -28.0 |
| *L. melanops* | 1 | 144530.5 | 39822.5 | 27.6 | 119164.8 | 82.4 | 25365.7 | 17.6 | 158987.3 | 14456.8 | 10.0 |
| *L. monticola* | 1 | 27566.9 | 3745.0 | 13.6 | 24584.8 | 89.2 | 2982.1 | 10.8 | 28329.8 | 762.9 | 2.8 |
| *L. multicolor* | 2 | 137461.2 | 20050.1 | 14.6 | 128096.3 | 93.2 | 9364.9 | 6.8 | 148146.4 | 10685.2 | 7.8 |
| *L. multimaculatus* | 1 | 103333.1 | 15405.4 | 14.9 | 98327.4 | 95.2 | 5005.7 | 4.8 | 113732.8 | 10399.7 | 10.1 |
| *L. nigriceps* | 2 | 12261.7 | 789.9 | 6.4 | 9905.9 | 80.8 | 2355.8 | 19.2 | 10695.8 | -1565.9 | -12.8 |
| *L. nigromaculatus* | 1 | 24503.1 | 1807.7 | 7.4 | 22465.8 | 91.7 | 2037.3 | 8.3 | 24273.5 | -229.6 | -0.9 |
| *L. nigroviridis* | 2 | 7880.1 | 557.9 | 7.1 | 7173.3 | 91.0 | 706.8 | 9.0 | 7731.2 | -148.9 | -1.9 |
| *L. nitidus* | 1 | 114813.6 | 9208.7 | 8.0 | 109990.2 | 95.8 | 4823.4 | 4.2 | 119198.9 | 4385.3 | 3.8 |
| *L. occipitalis* | 1 | 76655.6 | 6002.4 | 7.8 | 67841.9 | 88.5 | 8813.7 | 11.5 | 73844.2 | -2811.3 | -3.7 |
| *L. olongasta* | 1 | 106413.4 | 17680.0 | 16.6 | 102943.1 | 96.7 | 3470.2 | 3.3 | 120623.2 | 14209.8 | 13.4 |
| *L. orientalis* | 2 | 103472.1 | 20677.0 | 20.0 | 98165.7 | 94.9 | 5306.4 | 5.1 | 118842.7 | 15370.6 | 14.9 |
| *L. ornatus* | 2 | 254989.9 | 23527.2 | 9.2 | 251590.6 | 98.7 | 3399.3 | 1.3 | 275117.8 | 20127.9 | 7.9 |
| *L. pagaburoi* | 2 | 82030.4 | 37026.1 | 45.1 | 73510.1 | 89.6 | 8520.4 | 10.4 | 110536.2 | 28505.7 | 34.8 |
| *L. paulinae* | 2 | 883.6 | 107.0 | 12.1 | 806.7 | 91.3 | 77.0 | 8.7 | 913.7 | 30.1 | 3.4 |
| *L. petrophilus* | 2 | 366785.6 | 29077.3 | 7.9 | 304474.6 | 83.0 | 62311.0 | 17.0 | 333551.9 | -33233.7 | -9.1 |
| *L. pictus* | 2 | 238990.5 | 17510.9 | 7.3 | 227609.8 | 95.2 | 11380.6 | 4.8 | 245120.8 | 6130.3 | 2.6 |
| *L. platei* | 1 | 282900.0 | 52180.9 | 18.4 | 270080.1 | 95.5 | 12819.9 | 4.5 | 322261.0 | 39360.9 | 13.9 |
| *L. pseudoanomalus* | 1 | 184196.2 | 10482.3 | 5.7 | 159734.4 | 86.7 | 24461.8 | 13.3 | 170216.7 | -13979.5 | -7.6 |
| *L. pseudolemniscatus* | 1 | 43478.8 | 7054.9 | 16.2 | 43010.0 | 98.9 | 468.7 | 1.1 | 50064.9 | 6586.2 | 15.1 |
| *L. puna* | 2 | 243110.3 | 14584.5 | 6.0 | 173339.4 | 71.3 | 69770.9 | 28.7 | 187923.9 | -55186.4 | -22.7 |
| *L. quilmes* | 1 | 43780.8 | 8383.8 | 19.1 | 39264.8 | 89.7 | 4516.0 | 10.3 | 47648.6 | 3867.7 | 8.8 |
| *L. ramirezae* | 1 | 373872.6 | 17190.5 | 4.6 | 288043.9 | 77.0 | 85828.7 | 23.0 | 305234.3 | -68638.2 | -18.4 |
| *L. riojanus* | 1 | 120514.1 | 22581.7 | 18.7 | 114564.1 | 95.1 | 5950.0 | 4.9 | 137145.8 | 16631.7 | 13.8 |
| *L. rosenmannii* | 2 | 36956.0 | 5510.9 | 14.9 | 28425.8 | 76.9 | 8530.2 | 23.1 | 33936.7 | -3019.3 | -8.2 |
| *L. rothi* | 1 | 266230.7 | 20600.4 | 7.7 | 219161.2 | 82.3 | 47069.5 | 17.7 | 239761.6 | -26469.1 | -9.9 |
| *L. ruibali* | 2 | 50178.9 | 28712.9 | 57.2 | 43676.5 | 87.0 | 6502.4 | 13.0 | 72389.4 | 22210.5 | 44.3 |
| *L. sarmientoi* | 2 | 177723.2 | 11589.1 | 6.5 | 177380.7 | 99.8 | 342.5 | 0.2 | 188969.8 | 11246.6 | 6.3 |
| *L. scapularis* | 1 | 99423.3 | 24916.7 | 25.1 | 88801.7 | 89.3 | 10621.6 | 10.7 | 113718.4 | 14295.0 | 14.4 |
| *L. schroederi* | 2 | 269970.7 | 63423.1 | 23.5 | 243635.9 | 90.2 | 26334.8 | 9.8 | 307059.0 | 37088.4 | 13.7 |
| *L. scolaroi* | 2 | 12587.4 | 9091.5 | 72.2 | 11210.5 | 89.1 | 1376.9 | 10.9 | 20302.1 | 7714.6 | 61.3 |
| *L. silvanae* | 2 | 3462.8 | 220.5 | 6.4 | 3163.2 | 91.3 | 299.7 | 8.7 | 3383.7 | -79.2 | -2.3 |
| *L. somuncurae* | 2 | 25980.0 | 17315.5 | 66.6 | 24209.8 | 93.2 | 1770.2 | 6.8 | 41525.2 | 15545.2 | 59.8 |
| *L. stolzmannii* | 2 | 21613.8 | 0.0 | 0.0 | 21593.1 | 99.9 | 20.7 | 0.1 | 21593.1 | -20.7 | -0.1 |
| *L. tari* | 2 | 53014.0 | 7606.2 | 14.3 | 45555.1 | 85.9 | 7458.9 | 14.1 | 53161.3 | 147.2 | 0.3 |
| *L. tenuis* | 1 | 200967.7 | 5556.4 | 2.8 | 198967.3 | 99.0 | 2000.4 | 1.0 | 204523.7 | 3556.0 | 1.8 |
| *L. torresi* | 2 | 89738.9 | 9283.0 | 10.3 | 88154.7 | 98.2 | 1584.1 | 1.8 | 97437.8 | 7698.9 | 8.6 |
| *L. tristis* | 2 | 32362.3 | 1366.9 | 4.2 | 26356.2 | 81.4 | 6006.1 | 18.6 | 27723.2 | -4639.1 | -14.3 |
| *L. uspallatensis* | 1 | 21614.3 | 3229.1 | 14.9 | 21429.0 | 99.1 | 185.3 | 0.9 | 24658.1 | 3043.8 | 14.1 |
| *L. valdesianus* | 2 | 2201.2 | 221.2 | 10.0 | 1974.3 | 89.7 | 226.9 | 10.3 | 2195.5 | -5.7 | -0.3 |
| *L. wiegmannii* | 1 | 1437268.9 | 150819.1 | 10.5 | 1247006.6 | 86.8 | 190262.3 | 13.2 | 1397825.8 | -39443.2 | -2.7 |
| *L. xanthoviridis* | 1 | 56992.6 | 13121.2 | 23.0 | 33053.2 | 58.0 | 23939.5 | 42.0 | 46174.4 | -10818.3 | -19.0 |
| *L. zapallarensis* | 1 | 57175.1 | 7955.2 | 13.9 | 56759.9 | 99.3 | 415.2 | 0.7 | 64715.2 | 7540.1 | 13.2 |
| *L. zullyiae* | 2 | 6898.7 | 3064.1 | 44.4 | 6258.3 | 90.7 | 640.4 | 9.3 | 9322.4 | 2423.7 | 35.1 |
| *P. adrianae* | 2 | 36224.6 | 11590.0 | 32.0 | 27958.6 | 77.2 | 8266.1 | 22.8 | 39548.6 | 3324.0 | 9.2 |
| *P. agilis* | 2 | 14139.9 | 12147.0 | 85.9 | 11521.1 | 81.5 | 2618.8 | 18.5 | 23668.2 | 9528.2 | 67.4 |
| *P. ceii* | 2 | 69306.8 | 1600.0 | 2.3 | 59167.9 | 85.4 | 10139.0 | 14.6 | 60767.9 | -8539.0 | -12.3 |
| *P. excelsus* | 2 | 13996.4 | 3660.8 | 26.2 | 12365.0 | 88.3 | 1631.4 | 11.7 | 16025.8 | 2029.4 | 14.5 |
| *P. indistinctus* | 2 | 36252.0 | 10734.0 | 29.6 | 32697.5 | 90.2 | 3554.5 | 9.8 | 43431.5 | 7179.5 | 19.8 |
| *P. patagonicus* | 2 | 82070.1 | 18608.9 | 22.7 | 61174.7 | 74.5 | 20895.3 | 25.5 | 79783.6 | -2286.4 | -2.8 |
| *P. somuncurensis* | 2 | 32973.1 | 11288.3 | 34.2 | 26769.0 | 81.2 | 6204.1 | 18.8 | 38057.3 | 5084.2 | 15.4 |
| *P. spurcus* | 2 | 31644.2 | 5357.3 | 16.9 | 26154.7 | 82.7 | 5489.5 | 17.3 | 31512.0 | -132.3 | -0.4 |
| *P. tenebrosus* | 2 | 20439.2 | 2925.1 | 14.3 | 17159.7 | 84.0 | 3279.5 | 16.0 | 20084.8 | -354.4 | -1.7 |
| *P. vociferator* | 2 | 62428.5 | 6964.3 | 11.2 | 57037.7 | 91.4 | 5390.7 | 8.6 | 64002.0 | 1573.6 | 2.5 |
| *P. zapalensis* | 2 | 50382.5 | 24836.4 | 49.3 | 49779.6 | 98.8 | 602.9 | 1.2 | 74616.0 | 24233.5 | 48.1 |
| *S. aculeatus* | 1 | 135137.3 | 26565.3 | 19.7 | 107382.2 | 79.5 | 27755.1 | 20.5 | 133947.5 | -1189.7 | -0.9 |
| *S. angel* | 1 | 24984.5 | 5127.4 | 20.5 | 17421.1 | 69.7 | 7563.4 | 30.3 | 22548.4 | -2436.0 | -9.8 |
| *S. angulifer* | 1 | 96413.9 | 20607.5 | 21.4 | 81924.1 | 85.0 | 14489.8 | 15.0 | 102531.6 | 6117.7 | 6.3 |
| *S. apurimacus* | 1 | 203933.0 | 3810.8 | 1.9 | 193469.6 | 94.9 | 10463.4 | 5.1 | 197280.4 | -6652.6 | -3.3 |
| *S. azureus* | 1 | 1598474.4 | 40495.8 | 2.5 | 1487880.0 | 93.1 | 110594.4 | 6.9 | 1528375.8 | -70098.6 | -4.4 |
| *S. boettgeri* | 1 | 490376.8 | 345726.4 | 70.5 | 184701.5 | 37.7 | 305675.3 | 62.3 | 530428.0 | 40051.2 | 8.2 |
| *S. cadlei* | 1 | 86726.7 | 16092.0 | 18.6 | 74418.8 | 85.8 | 12307.8 | 14.2 | 90510.8 | 3784.2 | 4.4 |
| *S. caducus* | 1 | 2926552.5 | 247648.8 | 8.5 | 2587274.8 | 88.4 | 339277.6 | 11.6 | 2834923.7 | -91628.8 | -3.1 |
| *S. chota* | 1 | 18412.1 | 532.8 | 2.9 | 16026.5 | 87.0 | 2385.6 | 13.0 | 16559.3 | -1852.8 | -10.1 |
| *S. chrysopygus* | 1 | 57412.4 | 3625.8 | 6.3 | 46040.8 | 80.2 | 11371.7 | 19.8 | 49666.6 | -7745.9 | -13.5 |
| *S. doellojuradoi* | 1 | 430655.1 | 39903.6 | 9.3 | 380881.2 | 88.4 | 49773.9 | 11.6 | 420784.8 | -9870.3 | -2.3 |
| *S. dumerilii* | 1 | 459200.6 | 129763.9 | 28.3 | 347037.9 | 75.6 | 112162.8 | 24.4 | 476801.7 | 17601.1 | 3.8 |
| *S. empetrus* | 1 | 62092.2 | 13142.7 | 21.2 | 47596.4 | 76.7 | 14495.8 | 23.3 | 60739.2 | -1353.1 | -2.2 |
| *S. erythrogaster* | 1 | 309644.3 | 70226.9 | 22.7 | 281303.5 | 90.8 | 28340.8 | 9.2 | 351530.4 | 41886.1 | 13.5 |
| *S. festae* | 1 | 106181.0 | 21041.3 | 19.8 | 85029.1 | 80.1 | 21151.9 | 19.9 | 106070.4 | -110.7 | -0.1 |
| *S. fimbriatus* | 1 | 4324355.6 | 486091.2 | 11.2 | 3225173.9 | 74.6 | 1099181.7 | 25.4 | 3711265.0 | -613090.5 | -14.2 |
| *S. guentheri* | 1 | 32453.0 | 4578.6 | 14.1 | 28379.2 | 87.4 | 4073.8 | 12.6 | 32957.8 | 504.8 | 1.6 |
| *S. huancabambae* | 1 | 53239.7 | 19100.7 | 35.9 | 40055.1 | 75.2 | 13184.7 | 24.8 | 59155.8 | 5916.1 | 11.1 |
| *S. humeralis* | 1 | 70709.2 | 11634.9 | 16.5 | 64899.9 | 91.8 | 5809.3 | 8.2 | 76534.8 | 5825.6 | 8.2 |
| *S. imitator* | 1 | 37303.0 | 5256.8 | 14.1 | 29971.1 | 80.3 | 7331.9 | 19.7 | 35227.9 | -2075.0 | -5.6 |
| *S. iridescens* | 1 | 215831.3 | 13389.1 | 6.2 | 178708.6 | 82.8 | 37122.8 | 17.2 | 192097.7 | -23733.6 | -11.0 |
| *S. latebrosus* | 1 | 200361.3 | 52383.3 | 26.1 | 159097.8 | 79.4 | 41263.4 | 20.6 | 211481.1 | 11119.8 | 5.5 |
| *S. limitaris* | 1 | 120280.1 | 5927.0 | 4.9 | 118911.4 | 98.9 | 1368.7 | 1.1 | 124838.4 | 4558.3 | 3.8 |
| *S. marmoratus* | 1 | 1372098.2 | 43248.5 | 3.2 | 1228540.4 | 89.5 | 143557.8 | 10.5 | 1271788.9 | -100309.3 | -7.3 |
| *S. melanopygus* | 1 | 51707.8 | 9391.1 | 18.2 | 36316.6 | 70.2 | 15391.2 | 29.8 | 45707.7 | -6000.1 | -11.6 |
| *S. modestus* | 1 | 177363.2 | 2044.0 | 1.2 | 176784.5 | 99.7 | 578.8 | 0.3 | 178828.5 | 1465.2 | 0.8 |
| *S. ochoai* | 1 | 576841.2 | 18748.2 | 3.3 | 527689.7 | 91.5 | 49151.5 | 8.5 | 546437.9 | -30403.3 | -5.3 |
| *S. ornatissimus* | 1 | 342026.6 | 73078.0 | 21.4 | 286698.6 | 83.8 | 55328.0 | 16.2 | 359776.6 | 17750.0 | 5.2 |
| *S. ornatus* | 1 | 190656.9 | 27890.8 | 14.6 | 173285.5 | 90.9 | 17371.4 | 9.1 | 201176.2 | 10519.3 | 5.5 |
| *S. pectinatus* | 1 | 1434841.3 | 64569.0 | 4.5 | 1306365.8 | 91.0 | 128475.5 | 9.0 | 1370934.8 | -63906.5 | -4.5 |
| *S. percultus* | 1 | 492878.3 | 30347.4 | 6.2 | 482842.7 | 98.0 | 10035.6 | 2.0 | 513190.1 | 20311.8 | 4.1 |
| *S. prionotus* | 1 | 5660188.9 | 1251229.1 | 22.1 | 4719817.7 | 83.4 | 940371.2 | 16.6 | 5971046.8 | 310857.9 | 5.5 |
| *S. puyango* | 1 | 119294.6 | 12205.4 | 10.2 | 109064.6 | 91.4 | 10230.0 | 8.6 | 121270.1 | 1975.4 | 1.7 |
| *S. rhodomelas* | 1 | 29200.4 | 6650.0 | 22.8 | 26252.5 | 89.9 | 2948.0 | 10.1 | 32902.5 | 3702.0 | 12.7 |
| *S. roseiventris* | 1 | 9545380.6 | 1906608.4 | 20.0 | 8401161.2 | 88.0 | 1144219.4 | 12.0 | 10307769.6 | 762389.0 | 8.0 |
| *S. sinesaccus* | 1 | 3911739.1 | 425509.6 | 10.9 | 3873318.2 | 99.0 | 38420.9 | 1.0 | 4298827.7 | 387088.6 | 9.9 |
| *S. torquatus* | 1 | 225261.1 | 10760.6 | 4.8 | 195561.0 | 86.8 | 29700.1 | 13.2 | 206321.6 | -18939.5 | -8.4 |
| *S. trachycephalus* | 1 | 165230.9 | 16603.6 | 10.0 | 146402.9 | 88.6 | 18828.0 | 11.4 | 163006.5 | -2224.4 | -1.3 |
| *S. variabilis* | 1 | 173830.2 | 25122.6 | 14.5 | 160618.1 | 92.4 | 13212.1 | 7.6 | 185740.8 | 11910.6 | 6.9 |
| *S. varius* | 1 | 72428.8 | 20586.0 | 28.4 | 51384.8 | 70.9 | 21044.0 | 29.1 | 71970.8 | -458.0 | -0.6 |

**Table S4. Distributional changes between current and future SDMs using the MIROC5 model and considering the minimum scenario of greenhouse gases emissions (RCP 2.6). Reproductive mode (R), where (1=oviparous and 2=viviparous), Expansion areas (E), Areas with no changes (N) and contraction areas (C), Future geographic range (F) and Total range shifts (T).**

| **Species** | **R** | **Range (km^2^)** | **E (km^2^)** | **E %** | **N (km^2^)** | **N %** | **C (km^2^)** | **C %** | **F (km^2^)** | **ΔD (km^2^)** | **ΔT %** |
| --- | --- | --- | --- | --- | --- | --- | --- | --- | --- | --- | --- |
| *L. abaucan* | 1 | 90938.8 | 1740.8 | 1.9 | 75411.1 | 82.8 | 15536.2 | 17.1 | 77152.0 | -13795.4 | -15.2 |
| *L. albiceps* | 2 | 78919.6 | 16387.8 | 20.8 | 59180.5 | 75.0 | 19739.1 | 25.0 | 75568.4 | -3351.2 | -4.2 |
| *L. alticolor* | 2 | 208267.7 | 49236.8 | 23.6 | 194657.1 | 93.5 | 13610.6 | 6.5 | 243893.9 | 35626.2 | 17.1 |
| *L. andinus* | 2 | 205979.7 | 35308.7 | 17.1 | 191821.0 | 93.1 | 14158.7 | 6.9 | 227129.7 | 21150.0 | 10.3 |
| *L. anomalus* | 1 | 196109.6 | 25853.9 | 13.2 | 182475.8 | 93.0 | 13633.9 | 7.0 | 208329.6 | 12220.0 | 6.2 |
| *L. archeforus* | 2 | 17391.1 | 2016.4 | 11.6 | 16285.5 | 93.6 | 1105.6 | 6.4 | 18301.9 | 910.8 | 5.2 |
| *L. atacamensis* | 1 | 96535.8 | 19685.0 | 20.4 | 91962.8 | 95.2 | 4673.0 | 4.8 | 111647.9 | 15012.1 | 15.5 |
| *L. audituvelatus* | 2 | 101796.3 | 10729.7 | 10.5 | 88925.1 | 87.4 | 12871.1 | 12.6 | 99654.8 | -2141.5 | -2.1 |
| *L. austromendocinus* | 2 | 289262.7 | 27444.4 | 9.5 | 285179.6 | 98.6 | 4083.0 | 1.4 | 312624.1 | 23361.4 | 8.1 |
| *L. bellii* | 2 | 4047.9 | 324.5 | 8.0 | 3816.0 | 94.3 | 231.9 | 5.7 | 4140.5 | 92.6 | 2.3 |
| *L. bibronii* | 1 | 899817.2 | 58073.0 | 6.5 | 878639.9 | 97.6 | 21177.3 | 2.4 | 936712.9 | 36895.7 | 4.1 |
| *L. bitaeniatus* | 1 | 372936.1 | 50186.7 | 13.5 | 338101.6 | 90.7 | 34834.6 | 9.3 | 388288.3 | 15352.1 | 4.1 |
| *L. boulengeri* | 1 | 695035.0 | 33665.2 | 4.8 | 678740.1 | 97.7 | 16294.9 | 2.3 | 712405.4 | 17370.4 | 2.5 |
| *L. buergeri* | 2 | 107760.8 | 11344.0 | 10.5 | 105935.0 | 98.3 | 1825.8 | 1.7 | 117279.0 | 9518.1 | 8.8 |
| *L. canqueli* | 1 | 35544.7 | 6838.8 | 19.2 | 31607.6 | 88.9 | 3937.1 | 11.1 | 38446.4 | 2901.7 | 8.2 |
| *L. ceii* | 2 | 234795.8 | 79431.0 | 33.8 | 227336.9 | 96.8 | 7458.8 | 3.2 | 306767.9 | 71972.1 | 30.7 |
| *L. chacoensis* | 1 | 989974.1 | 7964.7 | 0.8 | 865960.4 | 87.5 | 124013.7 | 12.5 | 873925.1 | -116049.0 | -11.7 |
| *L. chaltin* | 1 | 50253.4 | 7178.2 | 14.3 | 50187.1 | 99.9 | 66.4 | 0.1 | 57365.2 | 7111.8 | 14.2 |
| *L. chiliensis* | 1 | 193882.0 | 4017.1 | 2.1 | 189060.2 | 97.5 | 4821.7 | 2.5 | 193077.4 | -804.6 | -0.4 |
| *L. constanzae* | 1 | 42970.9 | 7474.6 | 17.4 | 37392.0 | 87.0 | 5578.9 | 13.0 | 44866.6 | 1895.7 | 4.4 |
| *L. curicensis* | 2 | 9188.0 | 49.3 | 0.5 | 7691.8 | 83.7 | 1496.2 | 16.3 | 7741.2 | -1446.9 | -15.7 |
| *L. curis* | 2 | 315.9 | 130.9 | 41.4 | 303.8 | 96.2 | 12.1 | 3.8 | 434.6 | 118.8 | 37.6 |
| *L. cuyanus* | 1 | 192038.8 | 18802.9 | 9.8 | 182797.6 | 95.2 | 9241.2 | 4.8 | 201600.5 | 9561.7 | 5.0 |
| *L. cyanogaster* | 2 | 181410.0 | 26331.6 | 14.5 | 169096.5 | 93.2 | 12313.4 | 6.8 | 195428.1 | 14018.1 | 7.7 |
| *L. darwinii* | 1 | 1071745.3 | 61538.3 | 5.7 | 1004049.5 | 93.7 | 67695.8 | 6.3 | 1065587.8 | -6157.4 | -0.6 |
| *L. elongatus* | 2 | 237344.2 | 20095.7 | 8.5 | 219740.6 | 92.6 | 17603.6 | 7.4 | 239836.3 | 2492.1 | 1.0 |
| *L. escarchadosi* | 2 | 154111.2 | 16975.3 | 11.0 | 139385.5 | 90.4 | 14725.7 | 9.6 | 156360.9 | 2249.7 | 1.5 |
| *L. fabiani* | 2 | 43863.8 | 4009.8 | 9.1 | 37693.7 | 85.9 | 6170.1 | 14.1 | 41703.5 | -2160.3 | -4.9 |
| *L. fitzgeraldi* | 2 | 26571.9 | 5694.5 | 21.4 | 23455.3 | 88.3 | 3116.5 | 11.7 | 29149.9 | 2578.0 | 9.7 |
| *L. fitzingerii* | 1 | 313633.5 | 13677.7 | 4.4 | 293077.5 | 93.4 | 20556.0 | 6.6 | 306755.2 | -6878.3 | -2.2 |
| *L. fuscus* | 1 | 21660.1 | 2507.8 | 11.6 | 19680.7 | 90.9 | 1979.4 | 9.1 | 22188.5 | 528.4 | 2.4 |
| *L. gallardoi* | 2 | 105484.2 | 19155.6 | 18.2 | 93543.8 | 88.7 | 11940.4 | 11.3 | 112699.4 | 7215.2 | 6.8 |
| *L. goetschi* | 1 | 286784.8 | 2778.8 | 1.0 | 249498.9 | 87.0 | 37286.0 | 13.0 | 252277.6 | -34507.2 | -12.0 |
| *L. gracilis* | 1 | 747145.0 | 26045.3 | 3.5 | 680216.4 | 91.0 | 66928.7 | 9.0 | 706261.6 | -40883.4 | -5.5 |
| *L. gravenhorsti* | 2 | 5030.3 | 565.8 | 11.2 | 4773.7 | 94.9 | 256.6 | 5.1 | 5339.4 | 309.1 | 6.1 |
| *L. hatcheri* | 2 | 53393.0 | 4562.6 | 8.5 | 40699.9 | 76.2 | 12687.1 | 23.8 | 45262.5 | -8124.5 | -15.2 |
| *L. irregularis* | 2 | 157989.8 | 16888.0 | 10.7 | 144294.6 | 91.3 | 13695.3 | 8.7 | 161182.6 | 3192.7 | 2.0 |
| *L. isabelae* | 2 | 15416.4 | 80.3 | 0.5 | 12668.2 | 82.2 | 2748.3 | 17.8 | 12748.5 | -2668.0 | -17.3 |
| *L. jamesi* | 2 | 52157.7 | 553.0 | 1.1 | 39314.5 | 75.4 | 12843.3 | 24.6 | 39867.5 | -12290.2 | -23.6 |
| *L. kingii* | 2 | 256542.5 | 24919.8 | 9.7 | 237540.6 | 92.6 | 19001.9 | 7.4 | 262460.4 | 5917.9 | 2.3 |
| *L. koslowskyi* | 1 | 224339.8 | 16382.1 | 7.3 | 194834.5 | 86.8 | 29505.3 | 13.2 | 211216.6 | -13123.2 | -5.8 |
| *L. kriegi* | 2 | 240999.3 | 0.0 | 0.0 | 148904.1 | 61.8 | 92095.2 | 38.2 | 148904.1 | -92095.2 | -38.2 |
| *L. lavillai* | 2 | 83802.1 | 21458.5 | 25.6 | 69048.3 | 82.4 | 14753.8 | 17.6 | 90506.8 | 6704.7 | 8.0 |
| *L. lemniscatus* | 1 | 120069.2 | 5227.6 | 4.4 | 115390.4 | 96.1 | 4678.8 | 3.9 | 120618.0 | 548.8 | 0.5 |
| *L. leopardinus* | 2 | 1802.8 | 66.2 | 3.7 | 1475.3 | 81.8 | 327.6 | 18.2 | 1541.4 | -261.4 | -14.5 |
| *L. lineomaculatus* | 2 | 542059.2 | 13038.9 | 2.4 | 528342.2 | 97.5 | 13717.0 | 2.5 | 541381.1 | -678.1 | -0.1 |
| *L. lutzae* | 1 | 22466.6 | 2403.3 | 10.7 | 20098.8 | 89.5 | 2367.8 | 10.5 | 22502.1 | 35.5 | 0.2 |
| *L. magellanicus* | 2 | 168930.4 | 2124.3 | 1.3 | 167261.8 | 99.0 | 1668.6 | 1.0 | 169386.0 | 455.7 | 0.3 |
| *L. manueli* | 2 | 19445.0 | 1113.0 | 5.7 | 18767.6 | 96.5 | 677.4 | 3.5 | 19880.5 | 435.5 | 2.2 |
| *L. melanops* | 1 | 144530.5 | 22757.6 | 15.7 | 136293.3 | 94.3 | 8237.2 | 5.7 | 159050.9 | 14520.4 | 10.0 |
| *L. monticola* | 1 | 27566.9 | 2003.5 | 7.3 | 27527.5 | 99.9 | 39.3 | 0.1 | 29531.0 | 1964.2 | 7.1 |
| *L. multicolor* | 2 | 137461.2 | 23191.7 | 16.9 | 121815.0 | 88.6 | 15646.2 | 11.4 | 145006.7 | 7545.4 | 5.5 |
| *L. multimaculatus* | 1 | 103333.1 | 5603.4 | 5.4 | 97166.8 | 94.0 | 6166.4 | 6.0 | 102770.2 | -563.0 | -0.5 |
| *L. nigriceps* | 2 | 12261.7 | 844.7 | 6.9 | 9078.6 | 74.0 | 3183.1 | 26.0 | 9923.3 | -2338.4 | -19.1 |
| *L. nigromaculatus* | 1 | 24503.1 | 756.7 | 3.1 | 21875.1 | 89.3 | 2628.0 | 10.7 | 22631.7 | -1871.4 | -7.6 |
| *L. nigroviridis* | 2 | 7880.1 | 450.7 | 5.7 | 7504.6 | 95.2 | 375.5 | 4.8 | 7955.3 | 75.3 | 1.0 |
| *L. nitidus* | 1 | 114813.6 | 7451.8 | 6.5 | 112233.8 | 97.8 | 2579.8 | 2.2 | 119685.6 | 4872.1 | 4.2 |
| *L. occipitalis* | 1 | 76655.6 | 10474.2 | 13.7 | 73841.7 | 96.3 | 2813.9 | 3.7 | 84315.9 | 7660.4 | 10.0 |
| *L. olongasta* | 1 | 106413.4 | 2784.1 | 2.6 | 96759.5 | 90.9 | 9653.9 | 9.1 | 99543.7 | -6869.7 | -6.5 |
| *L. orientalis* | 2 | 103472.1 | 16596.6 | 16.0 | 98557.5 | 95.3 | 4914.6 | 4.7 | 115154.2 | 11682.1 | 11.3 |
| *L. ornatus* | 2 | 254989.9 | 12388.5 | 4.9 | 249008.7 | 97.7 | 5981.2 | 2.3 | 261397.2 | 6407.3 | 2.5 |
| *L. pagaburoi* | 2 | 82030.4 | 17209.5 | 21.0 | 72191.6 | 88.0 | 9838.8 | 12.0 | 89401.1 | 7370.6 | 9.0 |
| *L. paulinae* | 2 | 883.6 | 176.9 | 20.0 | 851.8 | 96.4 | 31.8 | 3.6 | 1028.7 | 145.1 | 16.4 |
| *L. petrophilus* | 2 | 366785.6 | 72829.5 | 19.9 | 320979.0 | 87.5 | 45806.6 | 12.5 | 393808.5 | 27022.9 | 7.4 |
| *L. pictus* | 2 | 238990.5 | 11308.8 | 4.7 | 234910.6 | 98.3 | 4079.9 | 1.7 | 246219.4 | 7228.9 | 3.0 |
| *L. platei* | 1 | 282900.0 | 10965.3 | 3.9 | 246894.1 | 87.3 | 36005.9 | 12.7 | 257859.5 | -25040.5 | -8.9 |
| *L. pseudoanomalus* | 1 | 184196.2 | 16761.2 | 9.1 | 178626.1 | 97.0 | 5570.1 | 3.0 | 195387.3 | 11191.1 | 6.1 |
| *L. pseudolemniscatus* | 1 | 43478.8 | 5549.4 | 12.8 | 41048.2 | 94.4 | 2430.5 | 5.6 | 46597.6 | 3118.9 | 7.2 |
| *L. puna* | 2 | 243110.3 | 25068.9 | 10.3 | 220153.3 | 90.6 | 22957.0 | 9.4 | 245222.2 | 2111.9 | 0.9 |
| *L. quilmes* | 1 | 43780.8 | 2587.9 | 5.9 | 38788.9 | 88.6 | 4992.0 | 11.4 | 41376.8 | -2404.1 | -5.5 |
| *L. ramirezae* | 1 | 373872.6 | 38978.3 | 10.4 | 324665.8 | 86.8 | 49206.8 | 13.2 | 363644.1 | -10228.5 | -2.7 |
| *L. riojanus* | 1 | 120514.1 | 17690.5 | 14.7 | 118468.9 | 98.3 | 2045.2 | 1.7 | 136159.4 | 15645.3 | 13.0 |
| *L. rosenmannii* | 2 | 36956.0 | 7168.5 | 19.4 | 31412.5 | 85.0 | 5543.5 | 15.0 | 38581.0 | 1625.0 | 4.4 |
| *L. rothi* | 1 | 266230.7 | 7505.1 | 2.8 | 234057.7 | 87.9 | 32173.0 | 12.1 | 241562.8 | -24667.9 | -9.3 |
| *L. ruibali* | 2 | 50178.9 | 3788.8 | 7.6 | 43961.7 | 87.6 | 6217.3 | 12.4 | 47750.5 | -2428.5 | -4.8 |
| *L. sarmientoi* | 2 | 177723.2 | 21008.5 | 11.8 | 169591.4 | 95.4 | 8131.8 | 4.6 | 190599.9 | 12876.6 | 7.2 |
| *L. scapularis* | 1 | 99423.3 | 7306.9 | 7.3 | 83814.6 | 84.3 | 15608.7 | 15.7 | 91121.5 | -8301.8 | -8.3 |
| *L. schroederi* | 2 | 269970.7 | 10418.7 | 3.9 | 232776.3 | 86.2 | 37194.3 | 13.8 | 243195.0 | -26775.6 | -9.9 |
| *L. scolaroi* | 2 | 12587.4 | 7878.5 | 62.6 | 11927.6 | 94.8 | 659.8 | 5.2 | 19806.1 | 7218.7 | 57.3 |
| *L. silvanae* | 2 | 3462.8 | 230.0 | 6.6 | 3231.4 | 93.3 | 231.4 | 6.7 | 3461.5 | -1.4 | 0.0 |
| *L. somuncurae* | 2 | 25980.0 | 4427.0 | 17.0 | 25243.1 | 97.2 | 736.9 | 2.8 | 29670.2 | 3690.2 | 14.2 |
| *L. stolzmannii* | 2 | 21613.8 | 19.7 | 0.1 | 21613.8 | 100.0 | 0.0 | 0.0 | 21633.5 | 19.7 | 0.1 |
| *L. tari* | 2 | 53014.0 | 8111.8 | 15.3 | 46507.3 | 87.7 | 6506.8 | 12.3 | 54619.1 | 1605.0 | 3.0 |
| *L. tenuis* | 1 | 200967.7 | 3643.3 | 1.8 | 198675.8 | 98.9 | 2291.9 | 1.1 | 202319.2 | 1351.4 | 0.7 |
| *L. torresi* | 2 | 89738.9 | 1628.7 | 1.8 | 78070.6 | 87.0 | 11668.3 | 13.0 | 79699.3 | -10039.6 | -11.2 |
| *L. tristis* | 2 | 32362.3 | 10583.7 | 32.7 | 31714.3 | 98.0 | 648.0 | 2.0 | 42298.0 | 9935.7 | 30.7 |
| *L. uspallatensis* | 1 | 21614.3 | 2234.5 | 10.3 | 19611.8 | 90.7 | 2002.5 | 9.3 | 21846.4 | 232.1 | 1.1 |
| *L. valdesianus* | 2 | 2201.2 | 87.8 | 4.0 | 1767.8 | 80.3 | 433.4 | 19.7 | 1855.6 | -345.6 | -15.7 |
| *L. wiegmannii* | 1 | 1437268.9 | 152552.6 | 10.6 | 1308692.9 | 91.1 | 128576.0 | 8.9 | 1461245.5 | 23976.5 | 1.7 |
| *L. xanthoviridis* | 1 | 56992.6 | 6084.0 | 10.7 | 51709.5 | 90.7 | 5283.1 | 9.3 | 57793.5 | 800.9 | 1.4 |
| *L. zapallarensis* | 1 | 57175.1 | 6249.0 | 10.9 | 55015.4 | 96.2 | 2159.7 | 3.8 | 61264.4 | 4089.3 | 7.2 |
| *L. zullyiae* | 2 | 6898.7 | 2435.2 | 35.3 | 6410.7 | 92.9 | 488.0 | 7.1 | 8845.9 | 1947.1 | 28.2 |
| *P. adrianae* | 2 | 36224.6 | 2625.3 | 7.2 | 31392.5 | 86.7 | 4832.2 | 13.3 | 34017.7 | -2206.9 | -6.1 |
| *P. agilis* | 2 | 14139.9 | 2405.0 | 17.0 | 8784.9 | 62.1 | 5355.0 | 37.9 | 11190.0 | -2949.9 | -20.9 |
| *P. ceii* | 2 | 69306.8 | 3338.4 | 4.8 | 60389.3 | 87.1 | 8917.5 | 12.9 | 63727.7 | -5579.1 | -8.0 |
| *P. excelsus* | 2 | 13996.4 | 2472.4 | 17.7 | 12978.6 | 92.7 | 1017.8 | 7.3 | 15451.0 | 1454.6 | 10.4 |
| *P. indistinctus* | 2 | 36252.0 | 6011.7 | 16.6 | 30862.5 | 85.1 | 5389.4 | 14.9 | 36874.2 | 622.3 | 1.7 |
| *P. patagonicus* | 2 | 82070.1 | 19663.1 | 24.0 | 65540.7 | 79.9 | 16529.3 | 20.1 | 85203.8 | 3133.8 | 3.8 |
| *P. somuncurensis* | 2 | 32973.1 | 2863.1 | 8.7 | 26106.5 | 79.2 | 6866.6 | 20.8 | 28969.6 | -4003.5 | -12.1 |
| *P. spurcus* | 2 | 31644.2 | 4343.3 | 13.7 | 30976.8 | 97.9 | 667.4 | 2.1 | 35320.1 | 3675.9 | 11.6 |
| *P. tenebrosus* | 2 | 20439.2 | 1400.2 | 6.9 | 17334.5 | 84.8 | 3104.7 | 15.2 | 18734.7 | -1704.5 | -8.3 |
| *P. vociferator* | 2 | 62428.5 | 3517.0 | 5.6 | 53850.7 | 86.3 | 8577.8 | 13.7 | 57367.7 | -5060.8 | -8.1 |
| *P. zapalensis* | 2 | 50382.5 | 12711.4 | 25.2 | 48151.7 | 95.6 | 2230.8 | 4.4 | 60863.1 | 10480.6 | 20.8 |
| *S. aculeatus* | 1 | 135137.3 | 29771.7 | 22.0 | 97533.8 | 72.2 | 37603.5 | 27.8 | 127305.5 | -7831.7 | -5.8 |
| *S. angel* | 1 | 24984.5 | 2876.5 | 11.5 | 20127.2 | 80.6 | 4857.3 | 19.4 | 23003.7 | -1980.8 | -7.9 |
| *S. angulifer* | 1 | 96413.9 | 19459.8 | 20.2 | 85788.3 | 89.0 | 10625.6 | 11.0 | 105248.1 | 8834.2 | 9.2 |
| *S. apurimacus* | 1 | 203933.0 | 18082.5 | 8.9 | 168122.7 | 82.4 | 35810.3 | 17.6 | 186205.2 | -17727.8 | -8.7 |
| *S. azureus* | 1 | 1598474.4 | 54102.1 | 3.4 | 1315251.6 | 82.3 | 283222.8 | 17.7 | 1369353.7 | -229120.7 | -14.3 |
| *S. boettgeri* | 1 | 490376.8 | 321230.5 | 65.5 | 186627.2 | 38.1 | 303749.6 | 61.9 | 507857.7 | 17480.9 | 3.6 |
| *S. cadlei* | 1 | 86726.7 | 16133.6 | 18.6 | 67905.4 | 78.3 | 18821.2 | 21.7 | 84039.0 | -2687.6 | -3.1 |
| *S. caducus* | 1 | 2926552.5 | 537296.7 | 18.4 | 2479476.2 | 84.7 | 447076.2 | 15.3 | 3016772.9 | 90220.4 | 3.1 |
| *S. chota* | 1 | 18412.1 | 1299.4 | 7.1 | 15714.6 | 85.3 | 2697.5 | 14.7 | 17014.0 | -1398.1 | -7.6 |
| *S. chrysopygus* | 1 | 57412.4 | 3333.7 | 5.8 | 49867.8 | 86.9 | 7544.6 | 13.1 | 53201.5 | -4210.9 | -7.3 |
| *S. doellojuradoi* | 1 | 430655.1 | 35248.8 | 8.2 | 390840.0 | 90.8 | 39815.0 | 9.2 | 426088.8 | -4566.3 | -1.1 |
| *S. dumerilii* | 1 | 459200.6 | 75333.3 | 16.4 | 403838.5 | 87.9 | 55362.1 | 12.1 | 479171.9 | 19971.2 | 4.3 |
| *S. empetrus* | 1 | 62092.2 | 15292.2 | 24.6 | 45267.9 | 72.9 | 16824.3 | 27.1 | 60560.1 | -1532.2 | -2.5 |
| *S. erythrogaster* | 1 | 309644.3 | 64356.6 | 20.8 | 285194.3 | 92.1 | 24450.0 | 7.9 | 349550.9 | 39906.6 | 12.9 |
| *S. festae* | 1 | 106181.0 | 20160.6 | 19.0 | 92112.1 | 86.8 | 14068.9 | 13.2 | 112272.7 | 6091.7 | 5.7 |
| *S. fimbriatus* | 1 | 4324355.6 | 828480.3 | 19.2 | 3740437.2 | 86.5 | 583918.3 | 13.5 | 4568917.5 | 244562.0 | 5.7 |
| *S. guentheri* | 1 | 32453.0 | 8271.7 | 25.5 | 27073.4 | 83.4 | 5379.6 | 16.6 | 35345.1 | 2892.1 | 8.9 |
| *S. huancabambae* | 1 | 53239.7 | 19775.6 | 37.1 | 26691.9 | 50.1 | 26547.8 | 49.9 | 46467.4 | -6772.3 | -12.7 |
| *S. humeralis* | 1 | 70709.2 | 15660.7 | 22.1 | 56320.5 | 79.7 | 14388.7 | 20.3 | 71981.2 | 1272.0 | 1.8 |
| *S. imitator* | 1 | 37303.0 | 7360.7 | 19.7 | 26860.6 | 72.0 | 10442.4 | 28.0 | 34221.3 | -3081.7 | -8.3 |
| *S. iridescens* | 1 | 215831.3 | 15556.6 | 7.2 | 202380.8 | 93.8 | 13450.5 | 6.2 | 217937.5 | 2106.2 | 1.0 |
| *S. latebrosus* | 1 | 200361.3 | 25794.3 | 12.9 | 188097.7 | 93.9 | 12263.5 | 6.1 | 213892.1 | 13530.8 | 6.8 |
| *S. limitaris* | 1 | 120280.1 | 15763.8 | 13.1 | 118889.6 | 98.8 | 1390.5 | 1.2 | 134653.3 | 14373.3 | 11.9 |
| *S. marmoratus* | 1 | 1372098.2 | 48087.3 | 3.5 | 1186068.3 | 86.4 | 186029.9 | 13.6 | 1234155.6 | -137942.6 | -10.1 |
| *S. melanopygus* | 1 | 51707.8 | 6517.7 | 12.6 | 42680.2 | 82.5 | 9027.6 | 17.5 | 49197.9 | -2509.9 | -4.9 |
| *S. modestus* | 1 | 177363.2 | 2800.5 | 1.6 | 177359.2 | 100.0 | 4.1 | 0.0 | 180159.6 | 2796.4 | 1.6 |
| *S. ochoai* | 1 | 576841.2 | 59940.2 | 10.4 | 572666.2 | 99.3 | 4175.0 | 0.7 | 632606.4 | 55765.2 | 9.7 |
| *S. ornatissimus* | 1 | 342026.6 | 77366.3 | 22.6 | 243290.9 | 71.1 | 98735.7 | 28.9 | 320657.2 | -21369.4 | -6.2 |
| *S. ornatus* | 1 | 190656.9 | 24388.2 | 12.8 | 175548.7 | 92.1 | 15108.2 | 7.9 | 199936.8 | 9279.9 | 4.9 |
| *S. pectinatus* | 1 | 1434841.3 | 34947.2 | 2.4 | 1278026.3 | 89.1 | 156815.0 | 10.9 | 1312973.5 | -121867.8 | -8.5 |
| *S. percultus* | 1 | 492878.3 | 50628.3 | 10.3 | 484561.8 | 98.3 | 8316.5 | 1.7 | 535190.1 | 42311.8 | 8.6 |
| *S. prionotus* | 1 | 5660188.9 | 1331648.0 | 23.5 | 4786567.3 | 84.6 | 873621.6 | 15.4 | 6118215.4 | 458026.4 | 8.1 |
| *S. puyango* | 1 | 119294.6 | 9773.5 | 8.2 | 114992.9 | 96.4 | 4301.7 | 3.6 | 124766.5 | 5471.9 | 4.6 |
| *S. rhodomelas* | 1 | 29200.4 | 3281.8 | 11.2 | 27973.0 | 95.8 | 1227.5 | 4.2 | 31254.8 | 2054.4 | 7.0 |
| *S. roseiventris* | 1 | 9545380.6 | 1250472.7 | 13.1 | 9330040.8 | 97.7 | 215339.8 | 2.3 | 10580513.5 | 1035132.9 | 10.8 |
| *S. sinesaccus* | 1 | 3911739.1 | 506705.6 | 13.0 | 3153991.0 | 80.6 | 757748.1 | 19.4 | 3660696.6 | -251042.5 | -6.4 |
| *S. torquatus* | 1 | 225261.1 | 4208.3 | 1.9 | 201689.9 | 89.5 | 23571.2 | 10.5 | 205898.3 | -19362.8 | -8.6 |
| *S. trachycephalus* | 1 | 165230.9 | 2489.6 | 1.5 | 150899.4 | 91.3 | 14331.6 | 8.7 | 153388.9 | -11842.0 | -7.2 |
| *S. variabilis* | 1 | 173830.2 | 26315.4 | 15.1 | 158790.3 | 91.3 | 15039.9 | 8.7 | 185105.7 | 11275.5 | 6.5 |
| *S. varius* | 1 | 72428.8 | 8690.1 | 12.0 | 59466.7 | 82.1 | 12962.2 | 17.9 | 68156.7 | -4272.1 | -5.9 |

**Table S5. Distributional changes between current and future SDMs using the MIROC5 model and considering the maximum scenario of greenhouse gases emissions (RCP 8.5). Reproductive mode (R), where (1=Oviparous and 2=Viviparous), Expansion areas (E), Areas with no changes (N) and contraction areas (C), Future geographic range (F) and Total range shifts (T).**

| **Species** | **R** | **Range (km^2^)** | **E (km^2^)** | **E %** | **N (km^2^)** | **N %** | **C (km^2^)** | **C %** | **F (km^2^)** | **ΔD (km^2^)** | **ΔT %** |
| --- | --- | --- | --- | --- | --- | --- | --- | --- | --- | --- | --- |
| *L. abaucan* | 1 | 90938.8 | 1180.7 | 1.3 | 72881.8 | 80.1 | 18067.6 | 19.8 | 74062.4 | -16887.0 | -18.6 |
| *L. albiceps* | 2 | 78919.6 | 18579.7 | 23.5 | 61144.0 | 77.5 | 17775.6 | 22.5 | 79723.7 | 804.1 | 1.0 |
| *L. alticolor* | 2 | 208267.7 | 35085.3 | 16.8 | 187197.3 | 89.9 | 21070.4 | 10.1 | 222282.6 | 14014.9 | 6.7 |
| *L. andinus* | 2 | 205979.7 | 28337.8 | 13.8 | 187298.7 | 90.9 | 18681.1 | 9.1 | 215636.5 | 9656.8 | 4.7 |
| *L. anomalus* | 1 | 196109.6 | 15974.2 | 8.1 | 183570.1 | 93.6 | 12539.5 | 6.4 | 199544.4 | 3434.7 | 1.8 |
| *L. archeforus* | 2 | 17391.1 | 3046.5 | 17.5 | 16815.1 | 96.7 | 576.0 | 3.3 | 19861.6 | 2470.5 | 14.2 |
| *L. atacamensis* | 1 | 96535.8 | 23736.7 | 24.6 | 84934.7 | 87.9 | 11701.1 | 12.1 | 108671.4 | 12035.6 | 12.5 |
| *L. audituvelatus* | 2 | 101796.3 | 19450.4 | 19.1 | 84376.3 | 82.9 | 17419.9 | 17.1 | 103826.7 | 2030.4 | 2.0 |
| *L. austromendocinus* | 2 | 289262.7 | 15442.2 | 5.3 | 251801.9 | 87.0 | 37460.8 | 13.0 | 267244.1 | -22018.5 | -7.6 |
| *L. bellii* | 2 | 4047.9 | 397.4 | 9.8 | 3606.2 | 89.1 | 441.7 | 10.9 | 4003.6 | -44.2 | -1.1 |
| *L. bibronii* | 1 | 899817.2 | 76683.5 | 8.5 | 847150.7 | 94.1 | 52666.6 | 5.9 | 923834.1 | 24016.9 | 2.7 |
| *L. bitaeniatus* | 1 | 372936.1 | 24313.2 | 6.5 | 288573.3 | 77.4 | 84362.8 | 22.6 | 312886.5 | -60049.6 | -16.1 |
| *L. boulengeri* | 1 | 695035.0 | 17599.1 | 2.5 | 641890.6 | 92.4 | 53144.4 | 7.6 | 659489.7 | -35545.3 | -5.1 |
| *L. buergeri* | 2 | 107760.8 | 21040.1 | 19.5 | 105495.3 | 97.9 | 2265.6 | 2.1 | 126535.4 | 18774.5 | 17.4 |
| *L. canqueli* | 1 | 35544.7 | 10066.5 | 28.3 | 29094.2 | 81.9 | 6450.6 | 18.1 | 39160.7 | 3616.0 | 10.2 |
| *L. ceii* | 2 | 234795.8 | 117328.7 | 50.0 | 198327.5 | 84.5 | 36468.2 | 15.5 | 315656.3 | 80860.5 | 34.4 |
| *L. chacoensis* | 1 | 989974.1 | 17833.4 | 1.8 | 907399.2 | 91.7 | 82574.9 | 8.3 | 925232.6 | -64741.5 | -6.5 |
| *L. chaltin* | 1 | 50253.4 | 9028.5 | 18.0 | 47974.3 | 95.5 | 2279.1 | 4.5 | 57002.8 | 6749.4 | 13.4 |
| *L. chiliensis* | 1 | 193882.0 | 6409.9 | 3.3 | 189945.7 | 98.0 | 3936.2 | 2.0 | 196355.6 | 2473.6 | 1.3 |
| *L. constanzae* | 1 | 42970.9 | 11577.9 | 26.9 | 37299.9 | 86.8 | 5671.0 | 13.2 | 48877.8 | 5906.9 | 13.7 |
| *L. curicensis* | 2 | 9188.0 | 177.3 | 1.9 | 5734.0 | 62.4 | 3454.1 | 37.6 | 5911.3 | -3276.8 | -35.7 |
| *L. curis* | 2 | 315.9 | 144.1 | 45.6 | 303.8 | 96.2 | 12.1 | 3.8 | 447.9 | 132.0 | 41.8 |
| *L. cuyanus* | 1 | 192038.8 | 16051.6 | 8.4 | 177155.6 | 92.2 | 14883.2 | 7.8 | 193207.2 | 1168.4 | 0.6 |
| *L. cyanogaster* | 2 | 181410.0 | 7929.3 | 4.4 | 169094.1 | 93.2 | 12315.9 | 6.8 | 177023.4 | -4386.6 | -2.4 |
| *L. darwinii* | 1 | 1071745.3 | 54541.2 | 5.1 | 972324.9 | 90.7 | 99420.3 | 9.3 | 1026866.2 | -44879.1 | -4.2 |
| *L. elongatus* | 2 | 237344.2 | 47389.8 | 20.0 | 203135.7 | 85.6 | 34208.5 | 14.4 | 250525.5 | 13181.3 | 5.6 |
| *L. escarchadosi* | 2 | 154111.2 | 18134.6 | 11.8 | 130085.6 | 84.4 | 24025.6 | 15.6 | 148220.2 | -5891.0 | -3.8 |
| *L. fabiani* | 2 | 43863.8 | 2930.2 | 6.7 | 33922.7 | 77.3 | 9941.1 | 22.7 | 36852.9 | -7010.9 | -16.0 |
| *L. fitzgeraldi* | 2 | 26571.9 | 9479.8 | 35.7 | 24499.3 | 92.2 | 2072.6 | 7.8 | 33979.1 | 7407.2 | 27.9 |
| *L. fitzingerii* | 1 | 313633.5 | 19143.9 | 6.1 | 277940.9 | 88.6 | 35692.6 | 11.4 | 297084.8 | -16548.7 | -5.3 |
| *L. fuscus* | 1 | 21660.1 | 3536.9 | 16.3 | 18890.4 | 87.2 | 2769.7 | 12.8 | 22427.3 | 767.2 | 3.5 |
| *L. gallardoi* | 2 | 105484.2 | 34309.6 | 32.5 | 89650.0 | 85.0 | 15834.2 | 15.0 | 123959.7 | 18475.4 | 17.5 |
| *L. goetschi* | 1 | 286784.8 | 13273.9 | 4.6 | 241386.8 | 84.2 | 45398.0 | 15.8 | 254660.7 | -32124.1 | -11.2 |
| *L. gracilis* | 1 | 747145.0 | 51174.7 | 6.8 | 629981.2 | 84.3 | 117163.8 | 15.7 | 681155.9 | -65989.1 | -8.8 |
| *L. gravenhorsti* | 2 | 5030.3 | 372.3 | 7.4 | 4165.3 | 82.8 | 865.0 | 17.2 | 4537.5 | -492.8 | -9.8 |
| *L. hatcheri* | 2 | 53393.0 | 8008.1 | 15.0 | 40471.1 | 75.8 | 12921.9 | 24.2 | 48479.2 | -4913.8 | -9.2 |
| *L. irregularis* | 2 | 157989.8 | 18057.2 | 11.4 | 138126.1 | 87.4 | 19863.7 | 12.6 | 156183.3 | -1806.6 | -1.1 |
| *L. isabelae* | 2 | 15416.4 | 278.0 | 1.8 | 11786.6 | 76.5 | 3629.8 | 23.5 | 12064.6 | -3351.8 | -21.7 |
| *L. jamesi* | 2 | 52157.7 | 1721.2 | 3.3 | 38704.9 | 74.2 | 13452.8 | 25.8 | 40426.1 | -11731.6 | -22.5 |
| *L. kingii* | 2 | 256542.5 | 54379.2 | 21.2 | 242614.7 | 94.6 | 13927.8 | 5.4 | 296993.8 | 40451.4 | 15.8 |
| *L. koslowskyi* | 1 | 224339.8 | 22212.8 | 9.9 | 172920.0 | 77.1 | 51419.8 | 22.9 | 195132.8 | -29207.0 | -13.0 |
| *L. kriegi* | 2 | 240999.3 | 1777.0 | 0.7 | 153812.4 | 63.8 | 87186.9 | 36.2 | 155589.4 | -85409.9 | -35.4 |
| *L. lavillai* | 2 | 83802.1 | 32435.8 | 38.7 | 59309.4 | 70.8 | 24492.7 | 29.2 | 91745.2 | 7943.2 | 9.5 |
| *L. lemniscatus* | 1 | 120069.2 | 8166.6 | 6.8 | 111796.5 | 93.1 | 8272.7 | 6.9 | 119963.1 | -106.1 | -0.1 |
| *L. leopardinus* | 2 | 1802.8 | 10.6 | 0.6 | 1200.0 | 66.6 | 602.9 | 33.4 | 1210.6 | -592.2 | -32.9 |
| *L. lineomaculatus* | 2 | 542059.2 | 23796.5 | 4.4 | 509637.5 | 94.0 | 32421.7 | 6.0 | 533434.0 | -8625.2 | -1.6 |
| *L. lutzae* | 1 | 22466.6 | 1417.3 | 6.3 | 16877.9 | 75.1 | 5588.8 | 24.9 | 18295.2 | -4171.4 | -18.6 |
| *L. magellanicus* | 2 | 168930.4 | 2004.4 | 1.2 | 165485.5 | 98.0 | 3444.8 | 2.0 | 167489.9 | -1440.5 | -0.9 |
| *L. manueli* | 2 | 19445.0 | 1571.7 | 8.1 | 7815.8 | 40.2 | 11629.2 | 59.8 | 9387.5 | -10057.5 | -51.7 |
| *L. melanops* | 1 | 144530.5 | 41375.0 | 28.6 | 115159.8 | 79.7 | 29370.7 | 20.3 | 156534.8 | 12004.3 | 8.3 |
| *L. monticola* | 1 | 27566.9 | 4543.8 | 16.5 | 27153.4 | 98.5 | 413.5 | 1.5 | 31697.2 | 4130.3 | 15.0 |
| *L. multicolor* | 2 | 137461.2 | 27708.8 | 20.2 | 112167.6 | 81.6 | 25293.6 | 18.4 | 139876.4 | 2415.1 | 1.8 |
| *L. multimaculatus* | 1 | 103333.1 | 3515.1 | 3.4 | 97700.7 | 94.5 | 5632.4 | 5.5 | 101215.9 | -2117.3 | -2.0 |
| *L. nigriceps* | 2 | 12261.7 | 1112.6 | 9.1 | 9845.0 | 80.3 | 2416.7 | 19.7 | 10957.7 | -1304.0 | -10.6 |
| *L. nigromaculatus* | 1 | 24503.1 | 1700.4 | 6.9 | 22063.2 | 90.0 | 2439.9 | 10.0 | 23763.7 | -739.5 | -3.0 |
| *L. nigroviridis* | 2 | 7880.1 | 421.3 | 5.3 | 7405.6 | 94.0 | 474.5 | 6.0 | 7826.9 | -53.2 | -0.7 |
| *L. nitidus* | 1 | 114813.6 | 13561.9 | 11.8 | 107823.7 | 93.9 | 6989.9 | 6.1 | 121385.6 | 6572.0 | 5.7 |
| *L. occipitalis* | 1 | 76655.6 | 10533.7 | 13.7 | 74572.6 | 97.3 | 2082.9 | 2.7 | 85106.4 | 8450.8 | 11.0 |
| *L. olongasta* | 1 | 106413.4 | 3365.0 | 3.2 | 94198.0 | 88.5 | 12215.3 | 11.5 | 97563.0 | -8850.4 | -8.3 |
| *L. orientalis* | 2 | 103472.1 | 31186.2 | 30.1 | 99034.7 | 95.7 | 4437.4 | 4.3 | 130220.9 | 26748.8 | 25.9 |
| *L. ornatus* | 2 | 254989.9 | 18199.1 | 7.1 | 236727.4 | 92.8 | 18262.5 | 7.2 | 254926.4 | -63.4 | 0.0 |
| *L. pagaburoi* | 2 | 82030.4 | 14207.7 | 17.3 | 66469.3 | 81.0 | 15561.1 | 19.0 | 80677.0 | -1353.4 | -1.6 |
| *L. paulinae* | 2 | 883.6 | 131.8 | 14.9 | 803.1 | 90.9 | 80.5 | 9.1 | 934.9 | 51.3 | 5.8 |
| *L. petrophilus* | 2 | 366785.6 | 92935.5 | 25.3 | 315473.4 | 86.0 | 51312.2 | 14.0 | 408409.0 | 41623.4 | 11.3 |
| *L. pictus* | 2 | 238990.5 | 16427.6 | 6.9 | 235989.8 | 98.7 | 3000.6 | 1.3 | 252417.5 | 13427.0 | 5.6 |
| *L. platei* | 1 | 282900.0 | 22228.3 | 7.9 | 229356.4 | 81.1 | 53543.6 | 18.9 | 251584.7 | -31315.3 | -11.1 |
| *L. pseudoanomalus* | 1 | 184196.2 | 29707.6 | 16.1 | 182522.1 | 99.1 | 1674.1 | 0.9 | 212229.8 | 28033.6 | 15.2 |
| *L. pseudolemniscatus* | 1 | 43478.8 | 7081.6 | 16.3 | 38982.3 | 89.7 | 4496.4 | 10.3 | 46064.0 | 2585.2 | 5.9 |
| *L. puna* | 2 | 243110.3 | 46149.1 | 19.0 | 194175.6 | 79.9 | 48934.6 | 20.1 | 240324.7 | -2785.5 | -1.1 |
| *L. quilmes* | 1 | 43780.8 | 5497.2 | 12.6 | 40011.8 | 91.4 | 3769.1 | 8.6 | 45509.0 | 1728.1 | 3.9 |
| *L. ramirezae* | 1 | 373872.6 | 54729.1 | 14.6 | 325743.3 | 87.1 | 48129.3 | 12.9 | 380472.4 | 6599.9 | 1.8 |
| *L. riojanus* | 1 | 120514.1 | 19852.3 | 16.5 | 115839.0 | 96.1 | 4675.1 | 3.9 | 135691.3 | 15177.2 | 12.6 |
| *L. rosenmannii* | 2 | 36956.0 | 6833.8 | 18.5 | 32295.9 | 87.4 | 4660.1 | 12.6 | 39129.7 | 2173.7 | 5.9 |
| *L. rothi* | 1 | 266230.7 | 10089.2 | 3.8 | 215043.5 | 80.8 | 51187.2 | 19.2 | 225132.7 | -41098.0 | -15.4 |
| *L. ruibali* | 2 | 50178.9 | 11341.9 | 22.6 | 46280.8 | 92.2 | 3898.1 | 7.8 | 57622.7 | 7443.7 | 14.8 |
| *L. sarmientoi* | 2 | 177723.2 | 5104.0 | 2.9 | 174652.2 | 98.3 | 3071.0 | 1.7 | 179756.2 | 2033.0 | 1.1 |
| *L. scapularis* | 1 | 99423.3 | 21597.5 | 21.7 | 91871.9 | 92.4 | 7551.5 | 7.6 | 113469.4 | 14046.1 | 14.1 |
| *L. schroederi* | 2 | 269970.7 | 22103.7 | 8.2 | 246167.4 | 91.2 | 23803.3 | 8.8 | 268271.1 | -1699.6 | -0.6 |
| *L. scolaroi* | 2 | 12587.4 | 9679.8 | 76.9 | 11540.4 | 91.7 | 1047.0 | 8.3 | 21220.2 | 8632.8 | 68.6 |
| *L. silvanae* | 2 | 3462.8 | 365.9 | 10.6 | 3035.5 | 87.7 | 427.3 | 12.3 | 3401.4 | -61.4 | -1.8 |
| *L. somuncurae* | 2 | 25980.0 | 11681.9 | 45.0 | 23234.1 | 89.4 | 2745.9 | 10.6 | 34916.0 | 8936.0 | 34.4 |
| *L. stolzmannii* | 2 | 21613.8 | 2158.6 | 10.0 | 8213.6 | 38.0 | 13400.2 | 62.0 | 10372.2 | -11241.6 | -52.0 |
| *L. tari* | 2 | 53014.0 | 12538.9 | 23.7 | 43511.8 | 82.1 | 9502.2 | 17.9 | 56050.7 | 3036.7 | 5.7 |
| *L. tenuis* | 1 | 200967.7 | 4567.8 | 2.3 | 198934.4 | 99.0 | 2033.4 | 1.0 | 203502.1 | 2534.4 | 1.3 |
| *L. torresi* | 2 | 89738.9 | 29834.3 | 33.2 | 89255.4 | 99.5 | 483.5 | 0.5 | 119089.7 | 29350.8 | 32.7 |
| *L. tristis* | 2 | 32362.3 | 17187.6 | 53.1 | 31002.1 | 95.8 | 1360.2 | 4.2 | 48189.7 | 15827.4 | 48.9 |
| *L. uspallatensis* | 1 | 21614.3 | 3845.0 | 17.8 | 19987.9 | 92.5 | 1626.4 | 7.5 | 23832.9 | 2218.7 | 10.3 |
| *L. valdesianus* | 2 | 2201.2 | 122.8 | 5.6 | 1801.1 | 81.8 | 400.1 | 18.2 | 1923.9 | -277.3 | -12.6 |
| *L. wiegmannii* | 1 | 1437268.9 | 128506.2 | 8.9 | 1313354.3 | 91.4 | 123914.7 | 8.6 | 1441860.4 | 4591.5 | 0.3 |
| *L. xanthoviridis* | 1 | 56992.6 | 1733.0 | 3.0 | 49008.3 | 86.0 | 7984.3 | 14.0 | 50741.3 | -6251.3 | -11.0 |
| *L. zapallarensis* | 1 | 57175.1 | 9722.7 | 17.0 | 51110.1 | 89.4 | 6065.0 | 10.6 | 60832.8 | 3657.7 | 6.4 |
| *L. zullyiae* | 2 | 6898.7 | 2709.0 | 39.3 | 5987.3 | 86.8 | 911.4 | 13.2 | 8696.4 | 1797.6 | 26.1 |
| *P. adrianae* | 2 | 36224.6 | 6851.6 | 18.9 | 26723.9 | 73.8 | 9500.7 | 26.2 | 33575.5 | -2649.1 | -7.3 |
| *P. agilis* | 2 | 14139.9 | 6788.4 | 48.0 | 10993.0 | 77.7 | 3146.9 | 22.3 | 17781.4 | 3641.5 | 25.8 |
| *P. ceii* | 2 | 69306.8 | 1310.8 | 1.9 | 48962.3 | 70.6 | 20344.6 | 29.4 | 50273.0 | -19033.8 | -27.5 |
| *P. excelsus* | 2 | 13996.4 | 2833.9 | 20.2 | 10537.3 | 75.3 | 3459.1 | 24.7 | 13371.1 | -625.3 | -4.5 |
| *P. indistinctus* | 2 | 36252.0 | 12113.6 | 33.4 | 29966.8 | 82.7 | 6285.2 | 17.3 | 42080.4 | 5828.5 | 16.1 |
| *P. patagonicus* | 2 | 82070.1 | 15294.4 | 18.6 | 63952.5 | 77.9 | 18117.5 | 22.1 | 79246.9 | -2823.2 | -3.4 |
| *P. somuncurensis* | 2 | 32973.1 | 2461.9 | 7.5 | 22784.0 | 69.1 | 10189.1 | 30.9 | 25245.9 | -7727.2 | -23.4 |
| *P. spurcus* | 2 | 31644.2 | 5063.9 | 16.0 | 30427.3 | 96.2 | 1216.9 | 3.8 | 35491.2 | 3846.9 | 12.2 |
| *P. tenebrosus* | 2 | 20439.2 | 1693.4 | 8.3 | 19070.9 | 93.3 | 1368.4 | 6.7 | 20764.3 | 325.0 | 1.6 |
| *P. vociferator* | 2 | 62428.5 | 16485.6 | 26.4 | 61600.9 | 98.7 | 827.5 | 1.3 | 78086.5 | 15658.1 | 25.1 |
| *P. zapalensis* | 2 | 50382.5 | 5586.5 | 11.1 | 41729.6 | 82.8 | 8652.9 | 17.2 | 47316.0 | -3066.4 | -6.1 |
| *S. aculeatus* | 1 | 135137.3 | 35578.7 | 26.3 | 98991.7 | 73.3 | 36145.6 | 26.7 | 134570.4 | -566.9 | -0.4 |
| *S. angel* | 1 | 24984.5 | 6624.8 | 26.5 | 17437.2 | 69.8 | 7547.2 | 30.2 | 24062.1 | -922.4 | -3.7 |
| *S. angulifer* | 1 | 96413.9 | 45195.5 | 46.9 | 42376.1 | 44.0 | 54037.8 | 56.0 | 87571.5 | -8842.4 | -9.2 |
| *S. apurimacus* | 1 | 203933.0 | 20115.8 | 9.9 | 170815.0 | 83.8 | 33118.0 | 16.2 | 190930.8 | -13002.2 | -6.4 |
| *S. azureus* | 1 | 1598474.4 | 69528.6 | 4.3 | 1436440.1 | 89.9 | 162034.4 | 10.1 | 1505968.7 | -92505.7 | -5.8 |
| *S. boettgeri* | 1 | 490376.8 | 105130.1 | 21.4 | 430855.4 | 87.9 | 59521.4 | 12.1 | 535985.5 | 45608.7 | 9.3 |
| *S. cadlei* | 1 | 86726.7 | 20194.5 | 23.3 | 58953.9 | 68.0 | 27772.7 | 32.0 | 79148.5 | -7578.2 | -8.7 |
| *S. caducus* | 1 | 2926552.5 | 543733.8 | 18.6 | 2004748.9 | 68.5 | 921803.5 | 31.5 | 2548482.8 | -378069.7 | -12.9 |
| *S. chota* | 1 | 18412.1 | 1698.9 | 9.2 | 15263.8 | 82.9 | 3148.3 | 17.1 | 16962.8 | -1449.4 | -7.9 |
| *S. chrysopygus* | 1 | 57412.4 | 2953.9 | 5.1 | 44792.3 | 78.0 | 12620.2 | 22.0 | 47746.2 | -9666.3 | -16.8 |
| *S. doellojuradoi* | 1 | 430655.1 | 42237.3 | 9.8 | 332737.0 | 77.3 | 97918.1 | 22.7 | 374974.3 | -55680.8 | -12.9 |
| *S. dumerilii* | 1 | 459200.6 | 234960.7 | 51.2 | 264577.0 | 57.6 | 194623.6 | 42.4 | 499537.7 | 40337.1 | 8.8 |
| *S. empetrus* | 1 | 62092.2 | 21527.3 | 34.7 | 46351.6 | 74.6 | 15740.6 | 25.4 | 67878.9 | 5786.7 | 9.3 |
| *S. erythrogaster* | 1 | 309644.3 | 56921.9 | 18.4 | 299973.1 | 96.9 | 9671.2 | 3.1 | 356894.9 | 47250.6 | 15.3 |
| *S. festae* | 1 | 106181.0 | 9999.4 | 9.4 | 78254.9 | 73.7 | 27926.1 | 26.3 | 88254.3 | -17926.7 | -16.9 |
| *S. fimbriatus* | 1 | 4324355.6 | 1476210.2 | 34.1 | 3084268.6 | 71.3 | 1240087.0 | 28.7 | 4560478.8 | 236123.2 | 5.5 |
| *S. guentheri* | 1 | 32453.0 | 10093.2 | 31.1 | 20954.7 | 64.6 | 11498.3 | 35.4 | 31047.9 | -1405.2 | -4.3 |
| *S. huancabambae* | 1 | 53239.7 | 20281.8 | 38.1 | 24300.5 | 45.6 | 28939.3 | 54.4 | 44582.2 | -8657.5 | -16.3 |
| *S. humeralis* | 1 | 70709.2 | 26392.0 | 37.3 | 44019.7 | 62.3 | 26689.5 | 37.7 | 70411.7 | -297.5 | -0.4 |
| *S. imitator* | 1 | 37303.0 | 9870.5 | 26.5 | 24477.2 | 65.6 | 12825.8 | 34.4 | 34347.7 | -2955.3 | -7.9 |
| *S. iridescens* | 1 | 215831.3 | 20700.5 | 9.6 | 209680.7 | 97.2 | 6150.7 | 2.8 | 230381.2 | 14549.8 | 6.7 |
| *S. latebrosus* | 1 | 200361.3 | 56831.2 | 28.4 | 146445.5 | 73.1 | 53915.7 | 26.9 | 203276.8 | 2915.5 | 1.5 |
| *S. limitaris* | 1 | 120280.1 | 14749.2 | 12.3 | 115169.5 | 95.8 | 5110.6 | 4.2 | 129918.7 | 9638.6 | 8.0 |
| *S. marmoratus* | 1 | 1372098.2 | 43287.1 | 3.2 | 1174269.1 | 85.6 | 197829.1 | 14.4 | 1217556.1 | -154542.1 | -11.3 |
| *S. melanopygus* | 1 | 51707.8 | 14592.5 | 28.2 | 38847.0 | 75.1 | 12860.8 | 24.9 | 53439.5 | 1731.7 | 3.3 |
| *S. modestus* | 1 | 177363.2 | 2287.7 | 1.3 | 176091.9 | 99.3 | 1271.3 | 0.7 | 178379.6 | 1016.4 | 0.6 |
| *S. ochoai* | 1 | 576841.2 | 66388.8 | 11.5 | 567799.5 | 98.4 | 9041.7 | 1.6 | 634188.3 | 57347.1 | 9.9 |
| *S. ornatissimus* | 1 | 342026.6 | 75277.7 | 22.0 | 213373.0 | 62.4 | 128653.6 | 37.6 | 288650.6 | -53375.9 | -15.6 |
| *S. ornatus* | 1 | 190656.9 | 41683.3 | 21.9 | 177922.0 | 93.3 | 12734.9 | 6.7 | 219605.3 | 28948.4 | 15.2 |
| *S. pectinatus* | 1 | 1434841.3 | 77279.7 | 5.4 | 1223295.7 | 85.3 | 211545.6 | 14.7 | 1300575.3 | -134266.0 | -9.4 |
| *S. percultus* | 1 | 492878.3 | 47775.6 | 9.7 | 473731.1 | 96.1 | 19147.3 | 3.9 | 521506.6 | 28628.3 | 5.8 |
| *S. prionotus* | 1 | 5660188.9 | 1680500.0 | 29.7 | 4263331.8 | 75.3 | 1396857.1 | 24.7 | 5943831.8 | 283642.9 | 5.0 |
| *S. puyango* | 1 | 119294.6 | 13287.1 | 11.1 | 107236.0 | 89.9 | 12058.6 | 10.1 | 120523.1 | 1228.5 | 1.0 |
| *S. rhodomelas* | 1 | 29200.4 | 7122.2 | 24.4 | 25626.1 | 87.8 | 3574.3 | 12.2 | 32748.3 | 3547.8 | 12.1 |
| *S. roseiventris* | 1 | 9545380.6 | 1216437.2 | 12.7 | 9080528.6 | 95.1 | 464852.0 | 4.9 | 10296965.8 | 751585.2 | 7.9 |
| *S. sinesaccus* | 1 | 3911739.1 | 533729.0 | 13.6 | 3102561.5 | 79.3 | 809177.5 | 20.7 | 3636290.6 | -275448.5 | -7.0 |
| *S. torquatus* | 1 | 225261.1 | 13863.4 | 6.2 | 193984.3 | 86.1 | 31276.8 | 13.9 | 207847.7 | -17413.4 | -7.7 |
| *S. trachycephalus* | 1 | 165230.9 | 25245.1 | 15.3 | 130412.0 | 78.9 | 34819.0 | 21.1 | 155657.0 | -9573.9 | -5.8 |
| *S. variabilis* | 1 | 173830.2 | 31767.3 | 18.3 | 149231.8 | 85.8 | 24598.4 | 14.2 | 180999.1 | 7168.9 | 4.1 |
| *S. varius* | 1 | 72428.8 | 25350.7 | 35.0 | 43580.4 | 60.2 | 28848.4 | 39.8 | 68931.1 | -3497.7 | -4.8 |

**Table S6. Magnitude and directions of range shifts movement between current and future predicted distributions under different models (GISS-ER and MIROC5) and different scenarios of greenhouse gases emissions (RCP 2.6 and 8.5). Reproductive mode (R), where 1=Oviparous and 2=Viviparous species, Magnitude of the centroid shift (M), Direction of the change (D) and future differences in elevation (E).**

|  |  |  | **GISS-ER (RCP 26)** | | | **GISS-ER (RCP 85)** | | | **MIROC5 (RCP 26)** | | | **MIROC5 (RCP 85)** | | |
| --- | --- | --- | --- | --- | --- | --- | --- | --- | --- | --- | --- | --- | --- | --- |
| **Species** | **R** | **Range (km²)** | **M (%)** | **D** | **Δ E (%)** | **M (%)** | **D** | **Δ E (%)** | **M (%)** | **D** | **Δ E (%)** | **M (%)** | **D** | **Δ E (%)** |
| *L. abaucan* | 1 | 45947.4 | 12.1 | S | -47.8 | 5.9 | S | -15.3 | 7.9 | N | -39.3 | 1.7 | N | -11.1 |
| *L. anomalus* | 1 | 195692.7 | 2.8 | S | -2.6 | 8.0 | SW | 3.7 | 6.6 | S | -4.9 | 1.3 | SW | 3.5 |
| *L. atacamensis* | 1 | 96506.7 | 5.4 | S | -13.7 | 10.2 | S | -45.8 | 14.4 | S | -10.8 | 2.7 | N | -61.1 |
| *L. bibronii* | 1 | 899472.2 | 3.3 | NE | 16.6 | 5.4 | NE | 12.6 | 6.7 | E | 11.5 | 0.9 | E | 29.2 |
| *L. bitaeniatus* | 1 | 372327.3 | 2.7 | W | -20.8 | 2.0 | W | -22.8 | 8.5 | SW | -11.0 | 1.4 | E | -12.3 |
| *L. boulengeri* | 1 | 694776.7 | 3.9 | SE | -28.1 | 2.5 | NE | -41.1 | 7.9 | N | -42.4 | 3.9 | SE | -31.2 |
| *L. canqueli* | 1 | 35544.7 | 6.9 | S | -0.3 | 11.3 | SW | 13.1 | 7.5 | W | 33.2 | 1.0 | E | -19.9 |
| *L. chacoensis* | 1 | 988699.3 | 2.1 | N | 7.9 | 9.6 | S | 9.4 | 4.4 | W | 11.1 | 1.1 | NW | 15.5 |
| *L. chaltin* | 1 | 50253.4 | 1.0 | W | -2.0 | 5.4 | S | 7.1 | 0.7 | W | -0.2 | 0.1 | SW | 5.7 |
| *L. chiliensis* | 1 | 191895.1 | 3.4 | W | -17.6 | 0.9 | N | -5.5 | 3.9 | S | -17.5 | 3.9 | S | -4.4 |
| *L. constanzae* | 1 | 42955.6 | 1.7 | NE | 1.7 | 2.8 | SE | -0.6 | 1.0 | S | -2.3 | 0.1 | SE | -2.4 |
| *L. cuyanus* | 1 | 191609.9 | 5.3 | N | -7.8 | 7.8 | N | -2.9 | 2.5 | N | -11.5 | 0.3 | NW | -3.4 |
| *L. darwinii* | 1 | 1070913.0 | 1.2 | SW | -1.1 | 3.2 | NW | 1.9 | 1.9 | SW | -0.4 | 0.1 | N | 5.2 |
| *L. fitzingerii* | 1 | 313539.5 | 8.5 | NE | -61.5 | 7.8 | N | -27.7 | 2.7 | N | -42.2 | 0.6 | N | -27.6 |
| *L. fuscus* | 1 | 20615.8 | 1.1 | W | -2.3 | 4.1 | SE | 2.4 | 6.0 | SW | -3.0 | 2.0 | SW | 5.3 |
| *L. goetschi* | 1 | 286656.6 | 2.0 | S | 10.7 | 14.0 | SW | -0.6 | 2.7 | S | 2.1 | 0.4 | SE | -5.3 |
| *L. gracilis* | 1 | 746411.5 | 2.2 | SW | 18.3 | 2.7 | SW | 21.7 | 1.6 | SW | 19.6 | 0.1 | SW | 23.0 |
| *L. koslowskyi* | 1 | 223303.6 | 5.5 | SW | -27.0 | 8.3 | NW | -31.2 | 8.9 | N | -3.7 | 2.6 | NW | -13.8 |
| *L. lemniscatus* | 1 | 118184.1 | 0.6 | S | -3.6 | 2.7 | SW | -13.3 | 1.0 | SE | -17.6 | 0.1 | SW | -4.5 |
| *L. lutzae* | 1 | 20038.4 | 1.4 | SW | 9.1 | 6.0 | SW | 7.9 | 5.2 | SW | 6.2 | 1.6 | SW | 8.1 |
| *L. melanops* | 1 | 144438.3 | 6.1 | SW | -1.6 | 17.2 | W | -5.3 | 1.4 | NE | -12.6 | 1.2 | S | -19.8 |
| *L. monticola* | 1 | 26842.5 | 3.4 | SE | 0.0 | 13.7 | NE | 11.8 | 10.9 | W | 4.0 | 36.0 | NW | 15.3 |
| *L. multimaculatus* | 1 | 101529.1 | 1.5 | NE | 7.3 | 6.5 | N | 8.4 | 3.6 | SW | -1.7 | 0.5 | SE | -6.7 |
| *L. nigromaculatus* | 1 | 24404.6 | 1.3 | S | 14.0 | 1.5 | NE | 9.5 | 5.2 | N | 9.5 | 0.9 | N | 9.0 |
| *L. nitidus* | 1 | 113290.6 | 0.7 | N | -17.0 | 1.7 | N | -43.1 | 1.3 | N | -18.4 | 0.1 | S | -16.9 |
| *L. occipitalis* | 1 | 75410.0 | 2.3 | SW | -27.4 | 5.0 | SW | -37.7 | 5.1 | N | 32.3 | 0.8 | N | -17.2 |
| *L. olongasta* | 1 | 106035.5 | 3.9 | NE | 2.8 | 6.7 | E | -0.6 | 6.6 | NW | 0.9 | 1.5 | N | 2.3 |
| *L. platei* | 1 | 282562.7 | 3.2 | SE | 11.1 | 12.9 | NE | 7.6 | 2.6 | W | 5.0 | 0.9 | S | 1.9 |
| *L. pseudoanomalus* | 1 | 184010.5 | 4.0 | W | 0.0 | 9.8 | N | 9.1 | 10.8 | N | -26.4 | 4.8 | NE | -10.2 |
| *L. pseudolemniscatus* | 1 | 43347.7 | 3.8 | N | 10.2 | 0.8 | E | 24.3 | 1.3 | N | 10.6 | 0.1 | N | 11.4 |
| *L. quilmes* | 1 | 43780.8 | 3.4 | NW | 0.6 | 2.5 | N | -12.8 | 6.7 | NE | -5.1 | 2.5 | NE | -17.3 |
| *L. ramirezae* | 1 | 373653.5 | 3.9 | S | 8.7 | 20.7 | S | -1.2 | 14.4 | NW | -5.1 | 4.1 | NW | 1.6 |
| *L. riojanus* | 1 | 120245.7 | 4.9 | E | -4.6 | 2.5 | SE | -14.2 | 1.2 | NE | -5.2 | 0.1 | NE | -22.8 |
| *L. rothi* | 1 | 266193.5 | 2.3 | S | -3.9 | 4.5 | S | 5.3 | 2.0 | S | -6.3 | 0.1 | S | 1.9 |
| *L. scapularis* | 1 | 99321.4 | 3.2 | N | -11.0 | 3.2 | NE | -21.4 | 9.4 | N | -25.6 | 17.9 | S | -16.2 |
| *L. tenuis* | 1 | 198938.0 | 2.3 | SE | 2.6 | 12.1 | S | 11.9 | 11.8 | S | 2.4 | 3.9 | SE | 12.6 |
| *L. uspallatensis* | 1 | 21614.3 | 7.2 | NE | 12.1 | 12.0 | NE | 18.4 | 2.5 | N | 13.8 | 0.5 | N | 20.9 |
| *L. wiegmannii* | 1 | 1429476.0 | 1.5 | NW | 2.8 | 6.9 | NE | 5.6 | 3.0 | N | 0.0 | 1.8 | NE | 6.3 |
| *L. xanthoviridis* | 1 | 56951.1 | 0.8 | S | 20.2 | 5.8 | SW | 22.9 | 5.4 | SE | 13.1 | 1.3 | SE | 15.8 |
| *L. zapallarensis* | 1 | 56763.5 | 3.0 | S | 9.3 | 4.9 | NE | 11.7 | 4.3 | N | 6.3 | 0.8 | N | 17.0 |
| *S. aculeatus* | 1 | 135137.3 | 0.5 | S | 22.0 | 4.9 | S | 42.3 | 7.4 | S | 11.7 | 0.8 | S | 29.0 |
| *S. angel* | 1 | 24984.5 | 1.3 | SW | 6.4 | 16.3 | SW | 12.6 | 15.8 | S | 5.3 | 2.3 | SW | 7.2 |
| *S. angulifer* | 1 | 96413.9 | 5.7 | N | -4.1 | 17.2 | N | -9.7 | 5.9 | N | -21.3 | 1.6 | NE | -26.8 |
| *S. apurimacus* | 1 | 203933.0 | 2.3 | NW | -6.3 | 1.2 | NW | -13.3 | 0.4 | NW | -6.6 | 0.1 | NW | -10.9 |
| *S. azureus* | 1 | 1598474.4 | 6.9 | NE | 15.1 | 2.3 | NE | -34.6 | 6.4 | NE | -19.1 | 11.9 | NE | -27.9 |
| *S. boettgeri* | 1 | 490376.8 | 0.8 | NW | 3.2 | 0.7 | NW | 26.4 | 0.9 | NW | 9.1 | 0.1 | NW | 22.2 |
| *S. cadlei* | 1 | 86726.7 | 13.2 | SW | 7.1 | 10.6 | SW | 21.6 | 26.9 | SW | 8.3 | 4.1 | SW | 24.3 |
| *S. caducus* | 1 | 2917990.0 | 0.4 | SW | 0.0 | 0.9 | SW | -31.9 | 0.6 | W | 6.9 | 0.1 | NW | -19.2 |
| *S. chota* | 1 | 18412.1 | 19.7 | S | -3.0 | 31.3 | S | -16.7 | 0.7 | S | -15.3 | 0.2 | S | -28.9 |
| *S. chrysopygus* | 1 | 57390.3 | 3.1 | S | -12.3 | 8.5 | SE | -1.5 | 4.4 | SE | -7.1 | 0.7 | S | -15.3 |
| *S. doellojuradoi* | 1 | 430655.1 | 0.7 | SW | 5.8 | 3.6 | SW | 19.5 | 1.8 | SW | 3.9 | 0.4 | SW | 20.9 |
| *S. dumerilii* | 1 | 458419.6 | 2.6 | E | 10.0 | 2.3 | S | 12.1 | 9.2 | S | 7.9 | 1.9 | SE | 10.2 |
| *S. empetrus* | 1 | 62092.2 | 3.1 | SE | -4.9 | 2.6 | SE | -15.7 | 4.6 | SE | -7.3 | 0.7 | SE | -12.3 |
| *S. erythrogaster* | 1 | 308447.8 | 7.4 | S | 12.4 | 8.0 | S | 18.9 | 3.9 | S | 14.8 | 2.9 | SW | 17.5 |
| *S. festae* | 1 | 105818.5 | 3.8 | N | 11.1 | 5.1 | NW | 18.5 | 2.3 | NW | 4.0 | 0.5 | N | 20.8 |
| *S. fimbriatus* | 1 | 4322001.8 | 1.4 | W | 15.2 | 5.8 | W | 21.5 | 2.9 | NW | 17.0 | 2.4 | NW | 22.6 |
| *S. guentheri* | 1 | 32453.0 | 1.3 | NE | -11.1 | 2.8 | NE | -21.0 | 3.5 | NE | -15.1 | 0.5 | NE | -29.7 |
| *S. huancabambae* | 1 | 53239.7 | 2.4 | E | -16.7 | 5.4 | E | -28.4 | 3.9 | E | -7.0 | 0.5 | E | -29.9 |
| *S. humeralis* | 1 | 70709.2 | 3.9 | NE | 3.3 | 2.2 | NE | 6.3 | 1.5 | NE | 1.2 | 0.3 | NE | 6.7 |
| *S. imitator* | 1 | 37303.0 | 1.3 | NE | 14.2 | 3.0 | NE | 30.7 | 3.5 | NE | 19.0 | 1.2 | NE | 42.2 |
| *S. iridescens* | 1 | 214720.2 | 12.9 | NE | 1.9 | 5.7 | N | 6.2 | 3.4 | N | 1.3 | 0.6 | NE | 0.1 |
| *S. latebrosus* | 1 | 200361.3 | 2.5 | SE | 7.4 | 9.1 | SE | 16.3 | 12.8 | SE | 14.7 | 2.7 | SE | 20.7 |
| *S. limitaris* | 1 | 120280.1 | 2.6 | SE | -16.4 | 3.5 | SE | -22.8 | 1.4 | SE | -12.2 | 3.6 | SE | -20.9 |
| *S. marmoratus* | 1 | 1372098.2 | 5.5 | SW | 2.8 | 2.6 | SW | 3.7 | 11.4 | S | 1.3 | 1.5 | SW | 1.7 |
| *S. melanopygus* | 1 | 51707.8 | 1.1 | SE | 7.0 | 1.8 | SE | 13.5 | 3.9 | SE | 11.2 | 0.5 | SE | 15.6 |
| *S. modestus* | 1 | 177363.2 | 3.0 | N | 1.9 | 0.4 | N | 9.6 | 2.1 | N | 1.3 | 0.2 | N | 8.2 |
| *S. ochoai* | 1 | 576841.2 | 0.4 | NW | -1.3 | 7.8 | NW | -3.9 | 5.1 | NW | -1.2 | 0.8 | NW | 1.0 |
| *S. ornatissimus* | 1 | 342026.6 | 2.9 | NW | 0.9 | 1.6 | NW | 7.4 | 2.7 | NW | 1.4 | 0.5 | NW | 17.4 |
| *S. ornatus* | 1 | 190284.6 | 8.6 | SE | -24.8 | 15.6 | SE | -38.3 | 2.6 | SE | -42.3 | 0.5 | SE | -30.4 |
| *S. pectinatus* | 1 | 1434841.3 | 3.8 | N | -0.9 | 13.7 | NW | -6.8 | 8.9 | NW | -6.0 | 1.9 | N | -17.1 |
| *S. percultus* | 1 | 492878.3 | 2.2 | N | 2.4 | 5.1 | N | 4.3 | 4.9 | N | 2.0 | 0.5 | N | 4.7 |
| *S. prionotus* | 1 | 5657301.0 | 0.4 | N | -14.5 | 3.3 | NW | -28.6 | 1.7 | NW | -10.8 | 0.3 | N | -4.8 |
| *S. puyango* | 1 | 119294.6 | 3.1 | SE | 21.7 | 7.8 | SE | 26.1 | 13.9 | SE | 30.5 | 5.8 | SE | 35.0 |
| *S. rhodomelas* | 1 | 29175.7 | 0.3 | S | -6.8 | 1.7 | N | -19.4 | 1.0 | SE | -40.3 | 0.1 | S | -39.9 |
| *S. roseiventris* | 1 | 9527283.1 | 10.5 | SE | 2.6 | 29.0 | SW | -3.1 | 3.4 | N | -8.4 | 1.0 | N | -12.1 |
| *S. sinesaccus* | 1 | 3911739.1 | 2.3 | N | 4.1 | 1.4 | NE | 10.2 | 12.0 | N | 10.1 | 2.3 | NE | 16.5 |
| *S. torquatus* | 1 | 225261.1 | 2.0 | NW | 7.3 | 10.0 | NW | 30.6 | 4.6 | NW | 7.4 | 0.6 | NW | 35.4 |
| *S. trachycephalus* | 1 | 164579.2 | 2.8 | NE | -22.7 | 8.0 | NE | -31.7 | 5.5 | SW | -63.7 | 0.6 | SW | -58.3 |
| *S. variabilis* | 1 | 173830.2 | 0.7 | NW | 7.2 | 3.8 | SW | 6.0 | 10.7 | SW | 1.2 | 7.6 | NW | -3.0 |
| *S. varius* | 1 | 72428.8 | 1.6 | SW | 7.9 | 6.3 | SW | 33.7 | 6.1 | SW | 22.4 | 10.1 | SW | 31.2 |
| *L. albiceps* | 2 | 78919.6 | 5.3 | S | -5.6 | 5.3 | SW | -7.2 | 7.1 | S | -9.3 | 4.3 | S | -7.8 |
| *L. alticolor* | 2 | 208115.9 | 2.2 | S | 4.3 | 0.2 | SE | 5.2 | 0.0 | S | 0.8 | 0.0 | SW | 2.8 |
| *L. andinus* | 2 | 205979.7 | 7.0 | SW | 12.7 | 8.6 | SW | 11.5 | 5.5 | SW | 19.4 | 1.8 | SW | 12.6 |
| *L. archeforus* | 2 | 17391.1 | 0.2 | NW | 15.9 | 1.2 | E | 44.2 | 1.1 | S | 13.5 | 0.1 | SW | 35.7 |
| *L. audituvelatus* | 2 | 101780.9 | 1.3 | N | 2.2 | 2.4 | N | 23.9 | 4.2 | SE | 1.4 | 1.2 | SE | 11.2 |
| *L. austromendocinus* | 2 | 289216.6 | 7.6 | SW | -1.0 | 4.2 | S | 19.3 | 8.7 | SE | 5.7 | 4.7 | W | 18.9 |
| *L. bellii* | 2 | 4047.9 | 1.6 | S | 27.1 | 3.1 | E | 31.1 | 6.5 | NW | 14.0 | 2.1 | NE | 26.0 |
| *L. buergeri* | 2 | 107760.8 | 1.6 | SW | 12.0 | 2.5 | S | 15.6 | 6.9 | S | 25.5 | 18.5 | S | 46.3 |
| *L. ceii* | 2 | 234726.6 | 1.2 | W | 8.2 | 1.9 | E | 22.1 | 0.7 | SW | 31.4 | 0.0 | NW | 90.3 |
| *L. curicensis* | 2 | 9187.9 | 8.6 | SW | 9.9 | 22.2 | SW | 34.2 | 5.8 | NE | 6.2 | 1.3 | N | 19.1 |
| *L. curis* | 2 | 315.9 | 5.6 | NW | 5.4 | 3.4 | W | 6.0 | 8.9 | W | 8.0 | 1.5 | SW | 9.9 |
| *L. cyanogaster* | 2 | 180531.7 | 1.6 | N | 10.9 | 6.1 | N | 24.5 | 12.8 | NE | 6.3 | 10.5 | N | 25.5 |
| *L. elongatus* | 2 | 237275.1 | 5.3 | S | 11.7 | 38.8 | NE | 50.8 | 1.0 | SW | 45.9 | 0.4 | E | 42.5 |
| *L. escarchadosi* | 2 | 154055.8 | 15.3 | N | 18.6 | 13.1 | SE | 44.9 | 5.1 | SW | 23.7 | 2.8 | S | 50.2 |
| *L. fabiani* | 2 | 43848.5 | 4.6 | SW | 5.1 | 6.7 | N | 17.6 | 28.9 | SW | 8.5 | 9.0 | E | 11.3 |
| *L. fitzgeraldi* | 2 | 26571.9 | 16.1 | NE | 28.5 | 9.5 | NE | 28.5 | 6.1 | N | 25.1 | 4.7 | NE | 38.8 |
| *L. gallardoi* | 2 | 105484.2 | 10.5 | N | 12.2 | 9.9 | NE | 30.0 | 15.8 | N | 8.2 | 7.4 | NE | 25.5 |
| *L. gravenhorsti* | 2 | 4445.8 | 3.1 | SW | 0.0 | 3.3 | SW | 0.0 | 18.7 | SW | 0.6 | 6.9 | SW | 0.6 |
| *L. hatcheri* | 2 | 53391.5 | 20.2 | SE | 43.4 | 21.0 | NE | 44.5 | 11.3 | N | 90.8 | 6.6 | N | 94.8 |
| *L. irregularis* | 2 | 157989.8 | 6.3 | SE | 6.0 | 20.2 | S | 34.2 | 3.0 | S | 0.1 | 1.8 | E | 22.2 |
| *L. isabelae* | 2 | 15416.4 | 1.6 | NE | 0.1 | 17.6 | S | 6.1 | 10.7 | NE | 6.4 | 8.3 | N | 16.2 |
| *L. jamesi* | 2 | 52157.7 | 2.4 | SW | 5.5 | 9.2 | NW | 2.0 | 8.4 | NW | 2.0 | 3.8 | NW | 6.4 |
| *L. kingii* | 2 | 256448.4 | 12.7 | SW | 9.4 | 16.3 | N | 16.5 | 12.1 | N | 14.2 | 4.5 | N | 19.6 |
| *L. kriegi* | 2 | 240930.1 | 5.2 | W | 8.2 | 7.4 | NW | 64.6 | 4.1 | N | 33.6 | 0.2 | NW | 61.9 |
| *L. lavillai* | 2 | 83802.1 | 2.8 | NW | -16.4 | 5.1 | NW | -15.6 | 4.5 | N | -10.3 | 0.5 | N | -17.8 |
| *L. leopardinus* | 2 | 1802.8 | 4.1 | NW | 23.0 | 1.2 | SE | 26.9 | 20.0 | S | 21.1 | 2.9 | S | 16.0 |
| *L. lineomaculatus* | 2 | 541913.0 | 2.9 | N | -4.1 | 8.7 | E | 1.7 | 5.0 | SE | 7.2 | 0.4 | S | 4.6 |
| *L. magellanicus* | 2 | 168852.5 | 2.7 | SE | 0.0 | 5.2 | SE | -29.4 | 3.4 | S | 0.0 | 0.2 | NE | 0.0 |
| *L. manueli* | 2 | 19443.6 | 3.6 | S | -6.8 | 8.8 | SW | -2.6 | 4.6 | NE | 17.1 | 0.3 | SW | -2.6 |
| *L. multicolor* | 2 | 137461.2 | 4.6 | NW | -4.5 | 7.9 | NW | -0.5 | 5.5 | N | -3.2 | 0.4 | NW | -0.4 |
| *L. nigriceps* | 2 | 12261.7 | 2.4 | N | 19.2 | 2.5 | NE | 13.6 | 17.3 | S | 4.4 | 0.7 | S | 4.4 |
| *L. nigroviridis* | 2 | 7880.1 | 2.5 | W | 0.0 | 4.2 | NW | -39.5 | 4.0 | NW | 36.5 | 0.4 | NW | 0.0 |
| *L. orientalis* | 2 | 103463.3 | 4.2 | NW | 0.9 | 2.9 | SW | -0.2 | 6.1 | NW | 4.9 | 1.1 | W | 11.9 |
| *L. ornatus* | 2 | 254967.1 | 2.7 | SE | -1.2 | 2.3 | SE | 0.9 | 1.7 | S | -1.4 | 0.1 | S | -2.1 |
| *L. pagaburoi* | 2 | 82028.9 | 11.4 | SW | 21.3 | 10.4 | S | 49.3 | 13.9 | S | 32.0 | 0.6 | E | 50.7 |
| *L. paulinae* | 2 | 868.3 | 2.4 | W | 0.0 | 7.3 | W | 0.0 | 2.8 | NW | 0.0 | 0.2 | W | 0.0 |
| *L. petrophilus* | 2 | 366712.1 | 4.0 | SE | 26.5 | 7.1 | SE | 21.6 | 8.7 | SE | 34.6 | 0.5 | SE | 30.6 |
| *L. pictus* | 2 | 238410.5 | 2.3 | W | 0.6 | 11.4 | N | 65.9 | 3.3 | NE | 83.5 | 0.4 | NE | 87.8 |
| *L. puna* | 2 | 243110.3 | 3.8 | S | -7.4 | 7.7 | SE | -14.8 | 8.9 | NW | 1.4 | 0.2 | N | -5.9 |
| *L. rosenmannii* | 2 | 36956.0 | 5.2 | N | 3.8 | 6.6 | S | 6.7 | 3.0 | N | 14.6 | 0.2 | N | 22.1 |
| *L. ruibali* | 2 | 50172.9 | 4.4 | N | 18.7 | 5.6 | NE | 53.6 | 4.8 | S | 11.7 | 0.3 | NE | 36.6 |
| *L. sarmientoi* | 2 | 177667.9 | 5.5 | E | 0.0 | 13.6 | E | 7.9 | 11.1 | NE | -0.6 | 1.0 | S | 3.0 |
| *L. schroederi* | 2 | 267923.6 | 2.4 | S | 25.0 | 3.6 | N | 31.5 | 3.0 | NE | 15.1 | 0.2 | S | 41.9 |
| *L. scolaroi* | 2 | 12587.4 | 0.6 | S | 9.3 | 6.3 | E | 20.1 | 4.4 | SE | 13.8 | 0.4 | SE | 22.6 |
| *L. silvanae* | 2 | 3462.8 | 2.7 | N | -1.3 | 4.2 | NW | 3.0 | 3.4 | W | 1.6 | 0.2 | NW | 4.6 |
| *L. somuncurae* | 2 | 25980.0 | 2.4 | N | 1.9 | 3.7 | NE | 15.9 | 1.5 | N | 12.2 | 0.1 | W | 22.6 |
| *L. stolzmannii* | 2 | 21572.6 | 4.5 | N | 10.1 | 5.2 | S | 13.1 | 6.9 | N | 10.4 | 0.3 | N | 12.6 |
| *L. tari* | 2 | 53014.0 | 3.3 | SW | -1.4 | 13.3 | SW | -2.2 | 5.4 | N | 5.1 | 0.4 | NE | -5.9 |
| *L. torresi* | 2 | 89615.6 | 2.6 | E | 4.8 | 7.1 | NW | 14.7 | 2.4 | NW | 5.7 | 0.2 | N | 19.9 |
| *L. tristis* | 2 | 32362.3 | 1.3 | NE | 8.9 | 12.7 | N | 23.5 | 4.8 | NE | 5.6 | 0.4 | N | 17.1 |
| *L. valdesianus* | 2 | 2201.2 | 4.3 | SE | 0.0 | 7.5 | E | 5.3 | 6.2 | E | 0.0 | 0.4 | E | 8.0 |
| *L. zullyiae* | 2 | 6898.7 | 0.0 | NE | 26.5 | 0.5 | S | 29.0 | 1.8 | SE | 32.3 | 0.2 | SE | 57.1 |
| *P. adrianae* | 2 | 35989.1 | 1.1 | NW | 24.3 | 3.0 | SE | 39.7 | 2.6 | N | 5.2 | 0.1 | N | 18.0 |
| *P. agilis* | 2 | 14139.9 | 2.6 | N | 23.2 | 2.9 | NE | 26.8 | 3.3 | NE | 1.3 | 0.2 | NE | 11.4 |
| *P. ceii* | 2 | 69306.8 | 2.3 | SW | 8.2 | 3.6 | SE | 13.7 | 3.4 | S | 7.7 | 0.1 | SE | 14.9 |
| *P. excelsus* | 2 | 13996.4 | 3.4 | N | 11.1 | 5.0 | NW | 15.9 | 5.3 | N | 14.3 | 0.3 | N | 22.7 |
| *P. indistinctus* | 2 | 36252.0 | 2.2 | W | 30.7 | 9.0 | SW | 48.6 | 7.0 | SW | 54.1 | 1.2 | SW | 29.8 |
| *P. patagonicus* | 2 | 82028.6 | 2.1 | S | 3.0 | 2.1 | S | 23.0 | 4.9 | SW | 15.1 | 0.1 | NE | 31.3 |
| *P. somuncurensis* | 2 | 32973.1 | 1.3 | NW | -0.5 | 1.8 | SW | -2.7 | 1.4 | N | -0.6 | 0.1 | N | -1.1 |
| *P. spurcus* | 2 | 31644.2 | 5.9 | N | 9.6 | 11.1 | NW | 21.4 | 5.6 | NE | 5.4 | 0.4 | NW | 19.8 |
| *P. tenebrosus* | 2 | 20434.5 | 5.6 | NE | 10.2 | 5.3 | S | 11.7 | 2.6 | SE | 9.3 | 0.2 | SE | 6.0 |
| *P. vociferator* | 2 | 62428.5 | 1.4 | S | 18.2 | 1.8 | NE | 62.8 | 1.0 | S | 16.2 | 0.1 | NE | 40.5 |

**Table S7. Phylogenetic multiple pairwise comparison of impacts of climate change among the ‘parity-by-taxonomy’ groups** **(*Stenocercus*, *Liolaemus*-oviparous, *Liolaemus*-viviparous and *Phymaturus*)** for each model (GISS-ER and MIROC5) and climate change scenario (RCP 2.6 and RCP 8.5).

| **Parameter** | **Model** | **Group I** | **Group J** | ***t*** | ***p value*** |
| --- | --- | --- | --- | --- | --- |
| Range shifts | GISS-ER model (RCP 2.6) | *Stenocercus* | *Liolaemus*-oviparous | 0.13 | 0.975 |
|  |  |  | *Liolaemus-*viviparous | -1.78 | 0.725 |
|  |  |  | *Phymaturus* | 0.84 | 0.731 |
|  |  | *Liolaemus*-oviparous | *Stenocercus* | -0.13 | 0.975 |
|  |  |  | *Liolaemus-*viviparous | -1.91 | **0.027** |
|  |  |  | *Phymaturus* | 0.75 | 0.828 |
|  |  | *Liolaemus-*viviparous | *Stenocercus* | 1.78 | 0.725 |
|  |  |  | *Liolaemus*-oviparous | 1.91 | **0.027** |
|  |  |  | *Phymaturus* | 1.98 | 0.540 |
|  |  | *Phymaturus* | *Stenocercus* | -0.84 | 0.731 |
|  |  |  | *Liolaemus*-oviparous | -0.75 | 0.828 |
|  |  |  | *Liolaemus-*viviparous | -1.98 | 0.540 |
|  | GISS-ER model (RCP 8.5) | *Stenocercus* | *Liolaemus*-oviparous | -0.24 | 0.954 |
|  |  |  | *Liolaemus-*viviparous | -1.87 | 0.707 |
|  |  |  | *Phymaturus* | -2.57 | 0.292 |
|  |  | *Liolaemus*-oviparous | *Stenocercus* | 0.24 | 0.954 |
|  |  |  | *Liolaemus-*viviparous | -1.61 | 0.056 |
|  |  |  | *Phymaturus* | -2.41 | 0.392 |
|  |  | *Liolaemus-*viviparous | *Stenocercus* | 1.87 | 0.707 |
|  |  |  | *Liolaemus*-oviparous | 1.61 | 0.056 |
|  |  |  | *Phymaturus* | -1.45 | 0.649 |
|  |  | *Phymaturus* | *Stenocercus* | 2.57 | 0.292 |
|  |  |  | *Liolaemus*-oviparous | 2.41 | 0.392 |
|  |  |  | *Liolaemus-*viviparous | 1.45 | 0.649 |
|  | MIROC5 model (RCP 2.6) | *Stenocercus* | *Liolaemus*-oviparous | -0.34 | 0.951 |
|  |  |  | *Liolaemus-*viviparous | -1.38 | 0.796 |
|  |  |  | *Phymaturus* | 0.35 | 0.897 |
|  |  | *Liolaemus*-oviparous | *Stenocercus* | 0.34 | 0.951 |
|  |  |  | *Liolaemus-*viviparous | -1.03 | 0.197 |
|  |  |  | *Phymaturus* | 0.57 | 0.856 |
|  |  | *Liolaemus-*viviparous | *Stenocercus* | 1.38 | 0.796 |
|  |  |  | *Liolaemus*-oviparous | 1.02 | 0.197 |
|  |  |  | *Phymaturus* | 1.13 | 0.856 |
|  |  | *Phymaturus* | *Stenocercus* | -0.35 | 0.897 |
|  |  |  | *Liolaemus*-oviparous | -0.57 | 0.856 |
|  |  |  | *Liolaemus-*viviparous | -1.24 | 0.698 |
|  | MIROC5 model (RCP 8.5) | *Stenocercus* | *Liolaemus*-oviparous | -0.52 | 0.928 |
|  |  |  | *Liolaemus-*viviparous | -1.32 | 0.801 |
|  |  |  | *Phymaturus* | -0.42 | 0.851 |
|  |  | *Liolaemus*-oviparous | *Stenocercus* | 0.52 | 0.928 |
|  |  |  | *Liolaemus-*viviparous | -0.76 | 0.321 |
|  |  |  | *Phymaturus* | -0.08 | 0.984 |
|  |  | *Liolaemus-*viviparous | *Stenocercus* | 1.32 | 0.801 |
|  |  |  | *Liolaemus*-oviparous | 0.76 | 0.321 |
|  |  |  | *Phymaturus* | 0.40 | 0.896 |
|  |  | *Phymaturus* | *Stenocercus* | 0.42 | 0.851 |
|  |  |  | *Liolaemus*-oviparous | 0.08 | 0.894 |
|  |  |  | *Liolaemus-*viviparous | -0.40 | 0.896 |
| Range contractions | GISS-ER model (RCP 2.6) | *Stenocercus* | *Liolaemus*-oviparous | -0.52 | 0.936 |
|  |  |  | *Liolaemus-*viviparous | -0.23 | 0.972 |
|  |  |  | *Phymaturus* | 2.42 | 0.676 |
|  |  | *Liolaemus*-oviparous | *Stenocercus* | 0.52 | 0.936 |
|  |  |  | *Liolaemus-*viviparous | 0.29 | 0.888 |
|  |  |  | *Phymaturus* | 2.74 | 0.465 |
|  |  | *Liolaemus-*viviparous | *Stenocercus* | 0.23 | 0.972 |
|  |  |  | *Liolaemus*-oviparous | -0.29 | 0.888 |
|  |  |  | *Phymaturus* | 2.55 | 0.512 |
|  |  | *Phymaturus* | *Stenocercus* | -2.42 | 0.676 |
|  |  |  | *Liolaemus*-oviparous | -2.74 | 0.465 |
|  |  |  | *Liolaemus-*viviparous | -2.55 | 0.512 |
|  | GISS-ER model (RCP 8.5) | *Stenocercus* | *Liolaemus*-oviparous | 1.19 | 0.866 |
|  |  |  | *Liolaemus-*viviparous | 1.93 | 0.806 |
|  |  |  | *Phymaturus* | -0.33 | 0.940 |
|  |  | *Liolaemus*-oviparous | *Stenocercus* | -1.19 | 0.866 |
|  |  |  | *Liolaemus-*viviparous | 0.64 | 0.594 |
|  |  |  | *Phymaturus* | -1.04 | 0.752 |
|  |  | *Liolaemus-*viviparous | *Stenocercus* | -1.93 | 0.806 |
|  |  |  | *Liolaemus*-oviparous | -0.64 | 0.594 |
|  |  |  | *Phymaturus* | -1.44 | 0.681 |
|  |  | *Phymaturus* | *Stenocercus* | 0.33 | 0.940 |
|  |  |  | *Liolaemus*-oviparous | 1.04 | 0.752 |
|  |  |  | *Liolaemus-*viviparous | 1.44 | 0.681 |
|  | MIROC5 model (RCP 2.6) | *Stenocercus* | *Liolaemus*-oviparous | -0.29 | 0.972 |
|  |  |  | *Liolaemus-*viviparous | 2.40 | 0.732 |
|  |  |  | *Phymaturus* | 1.18 | 0.828 |
|  |  | *Liolaemus*-oviparous | *Stenocercus* | 0.29 | 0.972 |
|  |  |  | *Liolaemus-*viviparous | 2.61 | **0.038** |
|  |  |  | *Phymaturus* | 1.37 | 0.759 |
|  |  | *Liolaemus-*viviparous | *Stenocercus* | -2.40 | 0.732 |
|  |  |  | *Liolaemus*-oviparous | -2.61 | **0.038** |
|  |  |  | *Phymaturus* | -0.56 | 0.904 |
|  |  | *Phymaturus* | *Stenocercus* | -1.18 | 0.828 |
|  |  |  | *Liolaemus*-oviparous | -1.37 | 0.759 |
|  |  |  | *Liolaemus-*viviparous | 0.56 | 0.904 |
|  | MIROC5 model (RCP 8.5) | *Stenocercus* | *Liolaemus*-oviparous | 0.16 | 0.978 |
|  |  |  | *Liolaemus-*viviparous | 1.45 | 0.878 |
|  |  |  | *Phymaturus* | 0.66 | 0.909 |
|  |  | *Liolaemus*-oviparous | *Stenocercus* | -0.16 | 0.978 |
|  |  |  | *Liolaemus-*viviparous | 1.18 | 0.431 |
|  |  |  | *Phymaturus* | 0.52 | 0.909 |
|  |  | *Liolaemus-*viviparous | *Stenocercus* | -1.45 | 0.878 |
|  |  |  | *Liolaemus*-oviparous | -1.18 | 0.431 |
|  |  |  | *Phymaturus* | -0.27 | 0.947 |
|  |  | *Phymaturus* | *Stenocercus* | -0.66 | 0.909 |
|  |  |  | *Liolaemus*-oviparous | -0.52 | 0.921 |
|  |  |  | *Liolaemus-*viviparous | 0.27 | 0.947 |
| Range expansions | GISS-ER model (RCP 2.6) | *Stenocercus* | *Liolaemus*-oviparous | 0.57 | 0.913 |
|  |  |  | *Liolaemus-*viviparous | -1.16 | 0.838 |
|  |  |  | *Phymaturus* | -0.18 | 0.809 |
|  |  | *Liolaemus*-oviparous | *Stenocercus* | -0.57 | 0.913 |
|  |  |  | *Liolaemus-*viviparous | -2.05 | **0.016** |
|  |  |  | *Phymaturus* | -0.60 | 0.874 |
|  |  | *Liolaemus-*viviparous | *Stenocercus* | 1.16 | 0.838 |
|  |  |  | *Liolaemus*-oviparous | 2.05 | **0.016** |
|  |  |  | *Phymaturus* | 0.59 | 0.862 |
|  |  | *Phymaturus* | *Stenocercus* | 0.18 | 0.809 |
|  |  |  | *Liolaemus*-oviparous | 0.60 | 0.874 |
|  |  |  | *Liolaemus-*viviparous | -0.59 | 0.862 |
|  | GISS-ER model (RCP 8.5) | *Stenocercus* | *Liolaemus*-oviparous | -0.62 | 0.931 |
|  |  |  | *Liolaemus-*viviparous | -3.20 | 0.670 |
|  |  |  | *Phymaturus* | -3.18 | 0.549 |
|  |  | *Liolaemus*-oviparous | *Stenocercus* | 0.62 | 0.931 |
|  |  |  | *Liolaemus-*viviparous | -2.65 | 0.100 |
|  |  |  | *Phymaturus* | -2.79 | 0.520 |
|  |  | *Liolaemus-*viviparous | *Stenocercus* | 3.20 | 0.670 |
|  |  |  | *Liolaemus*-oviparous | 2.65 | 0.100 |
|  |  |  | *Phymaturus* | -1.14 | 0.781 |
|  |  | *Phymaturus* | *Stenocercus* | 3.18 | 0.549 |
|  |  |  | *Liolaemus*-oviparous | 2.79 | 0.520 |
|  |  |  | *Liolaemus-*viviparous | 1.14 | 0.781 |
|  | MIROC5 model (RCP 2.6) | *Stenocercus* | *Liolaemus*-oviparous | 0.31 | 0.965 |
|  |  |  | *Liolaemus-*viviparous | -1.58 | 0.876 |
|  |  |  | *Phymaturus* | -0.73 | 0.879 |
|  |  | *Liolaemus*-oviparous | *Stenocercus* | -0.31 | 0.965 |
|  |  |  | *Liolaemus-*viviparous | -1.99 | 0.268 |
|  |  |  | *Phymaturus* | -0.93 | 0.810 |
|  |  | *Liolaemus-*viviparous | *Stenocercus* | 1.58 | 0.876 |
|  |  |  | *Liolaemus*-oviparous | 1.99 | 0.268 |
|  |  |  | *Phymaturus* | 0.15 | 0.971 |
|  |  | *Phymaturus* | *Stenocercus* | 0.73 | 0.879 |
|  |  |  | *Liolaemus*-oviparous | 0.93 | 0.810 |
|  |  |  | *Liolaemus-*viviparous | -0.15 | 0.971 |
|  | MIROC5 model (RCP 8.5) | *Stenocercus* | *Liolaemus*-oviparous | -0.08 | 0.994 |
|  |  |  | *Liolaemus-*viviparous | -3.18 | 0.678 |
|  |  |  | *Phymaturus* | -1.58 | 0.775 |
|  |  | *Liolaemus*-oviparous | *Stenocercus* | 0.08 | 0.994 |
|  |  |  | *Liolaemus-*viviparous | -3.32 | 0.070 |
|  |  |  | *Phymaturus* | -1.57 | 0.684 |
|  |  | *Liolaemus-*viviparous | *Stenocercus* | 3.32 | 0.678 |
|  |  |  | *Liolaemus*-oviparous | 3.32 | 0.070 |
|  |  |  | *Phymaturus* | 0.35 | 0.933 |
|  |  | *Phymaturus* | *Stenocercus* | 1.58 | 0.775 |
|  |  |  | *Liolaemus*-oviparous | 1.57 | 0.684 |
|  |  |  | *Liolaemus-*viviparous | -0.35 | 0.933 |
| Centroid shifts | GISS-ER model (RCP 2.6) | *Stenocercus* | *Liolaemus*-oviparous | 0.47 | 0.924 |
|  |  |  | *Liolaemus-*viviparous | -1.18 | 0.820 |
|  |  |  | *Phymaturus* | 0.42 | 0.870 |
|  |  | *Liolaemus*-oviparous | *Stenocercus* | -0.47 | 0.924 |
|  |  |  | *Liolaemus-*viviparous | -1.68 | **0.049** |
|  |  |  | *Phymaturus* | 0.11 | 0.974 |
|  |  | *Liolaemus-*viviparous | *Stenocercus* | 1.18 | 0.820 |
|  |  |  | *Liolaemus*-oviparous | 1.68 | **0.049** |
|  |  |  | *Phymaturus* | 1.18 | 0.691 |
|  |  | *Phymaturus* | *Stenocercus* | -0.42 | 0.870 |
|  |  |  | *Liolaemus*-oviparous | -0.11 | 0.974 |
|  |  |  | *Liolaemus-*viviparous | -1.18 | 0.691 |
|  | GISS-ER model (RCP 8.5) | *Stenocercus* | *Liolaemus*-oviparous | 0.03 | 0.992 |
|  |  |  | *Liolaemus-*viviparous | -0.92 | 0.857 |
|  |  |  | *Phymaturus* | 0.85 | 0.729 |
|  |  | *Liolaemus*-oviparous | *Stenocercus* | -0.03 | 0.992 |
|  |  |  | *Liolaemus-*viviparous | -0.95 | 0.242 |
|  |  |  | *Phymaturus* | 0.84 | 0.816 |
|  |  | *Liolaemus-*viviparous | *Stenocercus* | 0.92 | 0.857 |
|  |  |  | *Liolaemus*-oviparous | 0.95 | 0.242 |
|  |  |  | *Phymaturus* | 1.46 | 0.678 |
|  |  | *Phymaturus* | *Stenocercus* | -0.85 | 0.729 |
|  |  |  | *Liolaemus*-oviparous | -0.84 | 0.816 |
|  |  |  | *Liolaemus-*viviparous | -1.46 | 0.678 |
|  | MIROC5 model (RCP 2.6) | *Stenocercus* | *Liolaemus*-oviparous | 0.30 | 0.957 |
|  |  |  | *Liolaemus-*viviparous | -1.32 | 0.804 |
|  |  |  | *Phymaturus* | 0.94 | 0.715 |
|  |  | *Liolaemus*-oviparous | *Stenocercus* | -0.30 | 0.957 |
|  |  |  | *Liolaemus-*viviparous | -1.64 | **0.039** |
|  |  |  | *Phymaturus* | 0.74 | 0.823 |
|  |  | *Liolaemus-*viviparous | *Stenocercus* | 1.32 | 0.804 |
|  |  |  | *Liolaemus*-oviparous | 1.64 | **0.039** |
|  |  |  | *Phymaturus* | 1.80 | 0.568 |
|  |  | *Phymaturus* | *Stenocercus* | -0.94 | 0.715 |
|  |  |  | *Liolaemus*-oviparous | -0.74 | 0.823 |
|  |  |  | *Liolaemus-*viviparous | -1.80 | 0.568 |
|  | MIROC5 model (RCP 8.5) | *Stenocercus* | *Liolaemus*-oviparous | -0.83 | 0.889 |
|  |  |  | *Liolaemus-*viviparous | -0.40 | 0.948 |
|  |  |  | *Phymaturus* | 1.13 | 0.637 |
|  |  | *Liolaemus*-oviparous | *Stenocercus* | 0.83 | 0.889 |
|  |  |  | *Liolaemus-*viviparous | 0.48 | 0.515 |
|  |  |  | *Phymaturus* | 1.68 | 0.607 |
|  |  | *Liolaemus-*viviparous | *Stenocercus* | 0.40 | 0.948 |
|  |  |  | *Liolaemus*-oviparous | -0.48 | 0.515 |
|  |  |  | *Phymaturus* | 1.41 | 0.684 |
|  |  | *Phymaturus* | *Stenocercus* | -1.13 | 0.637 |
|  |  |  | *Liolaemus*-oviparous | -1.68 | 0.607 |
|  |  |  | *Liolaemus-*viviparous | -1.41 | 0.684 |
| Elevational shifts | GISS-ER model (RCP 2.6) | *Stenocercus* | *Liolaemus*-oviparous | 1.64 | 0.709 |
|  |  |  | *Liolaemus-*viviparous | -2.37 | 0.631 |
|  |  |  | *Phymaturus* | -2.81 | 0.267 |
|  |  | *Liolaemus*-oviparous | *Stenocercus* | -1.64 | 0.709 |
|  |  |  | *Liolaemus-*viviparous | -4.11 | **0.001** |
|  |  |  | *Phymaturus* | -3.89 | 0.096 |
|  |  | *Liolaemus-*viviparous | *Stenocercus* | 2.37 | 0.631 |
|  |  |  | *Liolaemus*-oviparous | 4.11 | **0.001** |
|  |  |  | *Phymaturus* | -1.39 | 0.679 |
|  |  | *Phymaturus* | *Stenocercus* | 2.81 | 0.267 |
|  |  |  | *Liolaemus*-oviparous | 3.89 | 0.096 |
|  |  |  | *Liolaemus-*viviparous | 1.39 | 0.679 |
|  | GISS-ER model (RCP 8.5) | *Stenocercus* | *Liolaemus*-oviparous | 1.02 | 0.825 |
|  |  |  | *Liolaemus-*viviparous | -3.47 | 0.462 |
|  |  |  | *Phymaturus* | -3.52 | 0.158 |
|  |  | *Liolaemus*-oviparous | *Stenocercus* | -1.02 | 0.825 |
|  |  |  | *Liolaemus-*viviparous | -4.55 | **0.001** |
|  |  |  | *Phymaturus* | -4.19 | 0.073 |
|  |  | *Liolaemus-*viviparous | *Stenocercus* | 3.47 | 0.462 |
|  |  |  | *Liolaemus*-oviparous | 4.55 | **0.001** |
|  |  |  | *Phymaturus* | -1.42 | 0.675 |
|  |  | *Phymaturus* | *Stenocercus* | 3.52 | 0.158 |
|  |  |  | *Liolaemus*-oviparous | 4.19 | 0.073 |
|  |  |  | *Liolaemus-*viviparous | 1.42 | 0.675 |
|  | MIROC5 model (RCP 2.6) | *Stenocercus* | *Liolaemus*-oviparous | 0.38 | 0.929 |
|  |  |  | *Liolaemus-*viviparous | -4.22 | 0.367 |
|  |  |  | *Phymaturus* | -2.28 | 0.320 |
|  |  | *Liolaemus*-oviparous | *Stenocercus* | -0.38 | 0.929 |
|  |  |  | *Liolaemus-*viviparous | -4.63 | **0.001** |
|  |  |  | *Phymaturus* | -2.53 | 0.352 |
|  |  | *Liolaemus-*viviparous | *Stenocercus* | 4.22 | 0.367 |
|  |  |  | *Liolaemus*-oviparous | 4.63 | **0.001** |
|  |  |  | *Phymaturus* | 0.34 | 0.922 |
|  |  | *Phymaturus* | *Stenocercus* | 2.28 | 0.320 |
|  |  |  | *Liolaemus*-oviparous | 2.53 | 0.352 |
|  |  |  | *Liolaemus-*viviparous | -0.34 | 0.922 |
|  | MIROC5 model (RCP 8.5) | *Stenocercus* | *Liolaemus*-oviparous | 0.70 | 0.877 |
|  |  |  | *Liolaemus-*viviparous | -4.55 | 0.322 |
|  |  |  | *Phymaturus* | -2.53 | 0.321 |
|  |  | *Liolaemus*-oviparous | *Stenocercus* | -0.70 | 0.877 |
|  |  |  | *Liolaemus-*viviparous | -5.29 | **0.001** |
|  |  |  | *Phymaturus* | -2.99 | 0.265 |
|  |  | *Liolaemus-*viviparous | *Stenocercus* | 4.55 | 0.322 |
|  |  |  | *Liolaemus*-oviparous | 5.29 | **0.001** |
|  |  |  | *Phymaturus* | 0.28 | 0.947 |
|  |  | *Phymaturus* | *Stenocercus* | 2.53 | 0.322 |
|  |  |  | *Liolaemus*-oviparous | 2.99 | 0.265 |
|  |  |  | *Liolaemus-*viviparous | -0.28 | 0.947 |

**Table S8. Spatial autocorrelation results (Global Moran's I) for each climate change model (GISS-ER and MIROC5) and each greenhouse gases emission scenario.**

| **Range shifts** | **Scenarios** | **Moran's Index** | **z-score** | ***p*-value** |
| --- | --- | --- | --- | --- |
| Range expansion | GISS-ER (RCP 2.6) | 0.747 | 899.144 | 0.000 |
|  | GISS-ER (RCP 8.5) | 0.797 | 899.978 | 0.000 |
|  | MIROC5 (RCP 2.6) | 0.719 | 833.661 | 0.000 |
|  | MIROC5 (RCP 8.5) | 0.735 | 855.431 | 0.000 |
| Range contraction | GISS-ER (RCP 2.6) | 0.805 | 957.257 | 0.000 |
|  | GISS-ER (RCP 8.5) | 0.861 | 1021.501 | 0.000 |
|  | MIROC5 (RCP 2.6) | 0.898 | 1068.145 | 0.000 |
|  | MIROC5 (RCP 8.5) | 0.836 | 982.086 | 0.000 |

**Table S9**. Climate change effects on range overlap between oviparous and viviparous species under different climate change models (GISS-ER and MIROC5) and different scenarios of greenhouse gases emissions (RCP 2.6 and 8.5).

| **Scenarios** | **Oviparous range (km²)** | **Viviparous range (km²)** | **Overlap (km²)** | **Overlap (%)** |
| --- | --- | --- | --- | --- |
| Today | 18227814.92 | 3247413.15 | 2627191.52 | 15.3 |
| GISS-ER (RCP 2.6) | 18630882.50 | 3299276.81 | 2631073.40 | 15.2 |
| GISS-ER (RCP 8.5) | 18520150.52 | 3349902.87 | 3113052.66 | 18.5 |
| MIROC5 (RCP 2.6) | 19179183.59 | 3326925.22 | 2694949.88 | 14.9 |
| MIROC5 (RCP 8.5) | 18994243.17 | 3308836.44 | 3159065.56 | 18.1 |

**Table S10.** **Performance of model selection using the Akaike Information Criteria (AICc score)**. Numbers in bold represent the lowest AICc values.

|  | **Regularization multiplier** | | | | | |
| --- | --- | --- | --- | --- | --- | --- |
| **Species** | **0.5** | **1** | **5** | **10** | **15** | **20** |
| *Liolaemus abaucan* | **300.1** | 343.2 | 350.1 | 354.0 | 358.7 | 373.0 |
| *Liolaemus albiceps* | **322.6** | 329.6 | 336.4 | 334.9 | 333.8 | 335.0 |
| *Liolaemus alticolor* | **600.4** | 600.5 | 616.7 | 619.8 | 624.6 | 627.6 |
| *Liolaemus andinus* | 510.3 | 544.5 | 497.7 | **486.9** | 513.2 | 515.1 |
| *Liolaemus anomalus* | 643.5 | 672.7 | **638.2** | 642.0 | 644.0 | 658.3 |
| *Liolaemus archeforus* | 240.9 | **236.5** | 277.4 | 269.6 | 286.2 | 351.6 |
| *Liolaemus atacamensis* | 312.5 | 313.2 | **259.0** | 263.2 | 261.4 | 261.3 |
| *Liolaemus audituvelatus* | **344.6** | 352.4 | 372.3 | 374.2 | 377.0 | 377.3 |
| *Liolaemus austromendocinus* | 1690.0 | 1353.4 | **1342.5** | 1354.3 | 1360.6 | 1380.2 |
| *Liolaemus bellii* | 467.6 | **450.0** | 494.7 | 536.2 | 558.2 | 563.7 |
| *Liolaemus bibronii* | 7390.4 | 4243.9 | **4184.2** | 4198.1 | 4211.3 | 4232.7 |
| *Liolaemus bitaeniatus* | 399.7 | **396.3** | 413.9 | 408.4 | 414.0 | 414.0 |
| *Liolaemus boulengeri* | 1815.4 | 1500.2 | **1478.7** | 1489.1 | 1495.1 | 1500.3 |
| *Liolaemus buergeri* | 562.5 | **516.1** | 524.0 | 554.8 | 562.8 | 571.5 |
| *Liolaemus canqueli* | 459.5 | **452.0** | 462.9 | 468.0 | 468.0 | 468.0 |
| *Liolaemus ceii* | 1172.5 | 546.8 | **529.3** | 543.3 | 545.4 | 546.2 |
| *Liolaemus chacoensis* | 832.0 | 892.8 | **830.0** | 833.0 | 842.1 | 850.6 |
| *Liolaemus chaltin* | **301.9** | 302.5 | 315.1 | 318.7 | 328.6 | 344.8 |
| *Liolaemus chiliensis* | 2105.6 | 1775.5 | **1751.6** | 1756.4 | 1775.9 | 1791.0 |
| *Liolaemus constanzae* | 1382.9 | 1207.3 | **1203.9** | 1222.4 | 1226.1 | 1247.1 |
| *Liolaemus curicensis* | 395.6 | **385.3** | 409.2 | 444.1 | 466.9 | 485.4 |
| *Liolaemus curis* | 307.6 | 232.6 | **219.1** | 226.8 | 251.2 | 268.9 |
| *Liolaemus cuyanus* | 750.3 | 761.1 | **720.7** | 729.2 | 752.1 | 756.9 |
| *Liolaemus cyanogaster* | 1188.4 | 1208.8 | **1148.3** | 1162.2 | 1174.1 | 1189.3 |
| *Liolaemus darwinii* | 5909.0 | **5533.1** | 5553.2 | 5564.5 | 5570.3 | 5588.2 |
| *Liolaemus elongatus* | 1643.3 | 1564.1 | **1531.1** | 1543.7 | 1584.1 | 1585.9 |
| *Liolaemus escarchadosi* | 1070.9 | 1086.6 | **1038.0** | 1049.1 | 1061.2 | 1071.2 |
| *Liolaemus fabiani* | 331.8 | **316.3** | 378.3 | 405.3 | 407.3 | 420.2 |
| *Liolaemus fitzgeraldi* | 464.3 | 496.6 | **449.3** | 472.3 | 474.4 | 474.4 |
| *Liolaemus fitzingerii* | 312.5 | 313.2 | **259.0** | 263.2 | 261.4 | 261.3 |
| *Liolaemus fuscus* | 488.9 | 485.8 | **424.6** | 455.3 | 460.3 | 461.9 |
| *Liolaemus gallardoi* | 312.5 | 313.2 | **259.0** | 263.2 | 261.4 | 261.3 |
| *Liolaemus goetschi* | 1422.7 | 1344.9 | **1086.9** | 1089.6 | 1103.4 | 1110.3 |
| *Liolaemus gracilis* | 2948.3 | 1765.5 | **1738.2** | 1750.5 | 1765.7 | 1765.5 |
| *Liolaemus gravenhorsti* | 502.6 | **346.1** | 374.4 | 394.7 | 399.2 | 404.6 |
| *Liolaemus hatcheri* | 388.4 | **385.1** | 412.2 | 417.8 | 415.0 | 417.6 |
| *Liolaemus irregularis* | 379.5 | 415.2 | **354.5** | 360.4 | 362.7 | 363.0 |
| *Liolaemus isabelae* | 579.6 | 570.1 | **534.6** | 558.5 | 562.1 | 562.7 |
| *Liolaemus jamesi* | 1513.9 | 700.5 | **697.1** | 707.9 | 728.5 | 739.5 |
| *Liolaemus kingii* | 1872.5 | **1841.7** | 1856.7 | 1891.0 | 1909.4 | 1908.0 |
| *Liolaemus koslowskyi* | 2295.2 | 2233.8 | **2214.4** | 2246.3 | 2292.1 | 2307.6 |
| *Liolaemus kriegi* | 837.4 | 839.9 | **805.2** | 822.8 | 835.0 | 830.5 |
| *Liolaemus lavillai* | 333.5 | **329.8** | 334.4 | 331.1 | 334.9 | 330.9 |
| *Liolaemus lemniscatus* | 4863.4 | **3882.3** | 3912.0 | 3937.6 | 3931.6 | 3975.5 |
| *Liolaemus leopardinus* | 410.1 | **403.4** | 441.5 | 468.1 | 488.7 | 490.6 |
| *Liolaemus lineomaculatus* | 2821.7 | 2775.3 | **2773.6** | 2808.3 | 2810.0 | 2823.5 |
| *Liolaemus lutzae* | **235.8** | 239.2 | 255.9 | 260.2 | 295.0 | 298.2 |
| *Liolaemus magellanicus* | 1577.1 | 1385.1 | **1384.6** | 1402.8 | 1403.5 | 1424.5 |
| *Liolaemus manueli* | 307.0 | 309.3 | **294.1** | 295.3 | 298.3 | 298.7 |
| *Liolaemus melanops* | 1283.4 | 821.6 | **809.3** | 820.8 | 822.9 | 820.5 |
| *Liolaemus monticola* | 409.1 | **371.8** | 395.2 | 408.3 | 409.8 | 424.4 |
| *Liolaemus multicolor* | 494.1 | **443.2** | 458.0 | 454.2 | 488.8 | 491.0 |
| *Liolaemus multimaculatus* | **391.4** | 418.0 | 420.1 | 455.6 | 465.4 | 467.9 |
| *Liolaemus nigriceps* | 565.0 | 591.7 | **542.9** | 555.0 | 561.9 | 565.2 |
| *Liolaemus nigromaculatus* | 446.4 | **429.1** | 430.4 | 449.1 | 462.3 | 475.3 |
| *Liolaemus nigroviridis* | 861.5 | **773.6** | 790.1 | 830.4 | 870.9 | 900.9 |
| *Liolaemus nitidus* | 1015.2 | 1217.1 | **945.8** | 958.0 | 967.0 | 982.1 |
| *Liolaemus occipitalis* | 449.4 | 649.8 | **417.0** | 436.8 | 449.6 | 466.0 |
| *Liolaemus olongasta* | 443.4 | **440.0** | 486.4 | 527.5 | 539.7 | 545.4 |
| *Liolaemus orientalis* | 366.8 | **348.8** | 358.2 | 375.1 | 375.2 | 375.2 |
| *Liolaemus ornatus* | 1579.9 | 1487.1 | **1461.0** | 1462.8 | 1474.9 | 1487.1 |
| *Liolaemus pagaburoi* | 342.6 | **314.5** | 386.8 | 402.0 | 398.9 | 398.8 |
| *Liolaemus paulinae* | 342.5 | **318.4** | 319.1 | 337.1 | 383.2 | 410.5 |
| *Liolaemus petrophilus* | 1979.7 | **1948.7** | 1970.6 | 2025.0 | 2055.2 | 2057.0 |
| *Liolaemus pictus* | 2831.3 | 2906.9 | **2797.7** | 2820.7 | 2843.8 | 2852.2 |
| *Liolaemus platei* | 973.6 | 966.5 | **931.0** | 960.2 | 977.1 | 975.0 |
| *Liolaemus pseudoanomalus* | **381.9** | 392.3 | 414.6 | 435.9 | 432.5 | 439.8 |
| *Liolaemus pseudolemniscatus* | **325.8** | 333.1 | 335.1 | 343.7 | 351.0 | 351.1 |
| *Liolaemus puna* | 1709.6 | 695.2 | **616.2** | 653.2 | 661.1 | 671.9 |
| *Liolaemus quilmes* | **914.0** | 952.6 | 931.8 | 940.9 | 953.6 | 969.6 |
| *Liolaemus ramirezae* | 492.6 | **388.5** | 396.7 | 391.7 | 400.7 | 402.4 |
| *Liolaemus riojanus* | 452.3 | **364.4** | 369.3 | 367.7 | 371.8 | 372.1 |
| *Liolaemus rosenmannii* | 1094.8 | **1072.6** | 1097.1 | 1108.6 | 1122.8 | 1129.7 |
| *Liolaemus rothi* | 2383.2 | **2057.2** | 2093.2 | 2157.8 | 2183.0 | 2221.6 |
| *Liolaemus ruibali* | 377.5 | **334.3** | 381.2 | 396.1 | 400.4 | 405.7 |
| *Liolaemus sarmientoi* | 689.3 | 724.4 | **674.5** | 681.0 | 690.4 | 693.9 |
| *Liolaemus scapularis* | 605.1 | 324.7 | **304.7** | 314.4 | 325.6 | 342.5 |
| *Liolaemus schroederi* | 846.8 | 933.5 | **784.8** | 827.6 | 849.2 | 873.9 |
| *Liolaemus scolaroi* | 263.6 | **261.0** | 291.8 | 304.8 | 315.3 | 328.7 |
| *Liolaemus silvanae* | 437.2 | **424.0** | 432.7 | 449.6 | 467.5 | 479.7 |
| *Liolaemus somuncurae* | 313.2 | 270.5 | **262.1** | 278.4 | 302.0 | 315.6 |
| *Liolaemus stolzmannii* | 246.1 | 240.4 | **238.8** | 238.9 | 238.8 | 238.8 |
| *Liolaemus tari* | 502.8 | **417.8** | 421.2 | 422.7 | 422.4 | 422.8 |
| *Liolaemus tenuis* | 5915.5 | 5198.0 | 5193.3 | **5191.3** | 5207.8 | 5212.5 |
| *Liolaemus torresi* | 513.7 | 519.9 | 493.9 | **502.7** | 509.6 | 514.4 |
| *Liolaemus tristis* | **337.8** | 344.1 | 350.2 | 383.6 | 379.3 | 392.0 |
| *Liolaemus uspallatensis* | 310.1 | **285.6** | 314.7 | 326.1 | 343.6 | 366.9 |
| *Liolaemus valdesianus* | 484.8 | **369.5** | 422.9 | 432.6 | 432.0 | 432.0 |
| *Liolaemus wiegmannii* | 3877.1 | **3608.7** | 3620.7 | 3623.3 | 3628.2 | 3665.3 |
| *Liolaemus xanthoviridis* | 327.7 | **301.6** | 313.4 | 307.6 | 307.7 | 308.2 |
| *Liolaemus zapallarensis* | 437.9 | 392.4 | **389.6** | 416.4 | 439.5 | 447.4 |
| *Liolaemus zullyiae* | 274.4 | **264.9** | 288.0 | 326.2 | 352.6 | 359.5 |
| *Phymaturus adrianae* | **420.0** | 445.4 | 449.8 | 446.6 | 447.5 | 447.5 |
| *Phymaturus agilis* | **312.1** | 316.4 | 348.9 | 376.7 | 386.1 | 374.8 |
| *Phymaturus ceii* | 321.2 | **311.7** | 355.0 | 355.7 | 358.9 | 358.4 |
| *Phymaturus excelsus* | **554.6** | 573.8 | 650.6 | 681.7 | 702.0 | 709.4 |
| *Phymaturus indistinctus* | 302.7 | **298.6** | 337.4 | 345.9 | 372.5 | 379.9 |
| *Phymaturus patagonicus* | 416.6 | **405.3** | 407.6 | 407.0 | 412.3 | 419.3 |
| *Phymaturus somuncurensis* | **504.2** | 510.8 | 550.0 | 538.4 | 570.7 | 539.6 |
| *Phymaturus spurcus* | 1202.7 | **462.2** | 467.5 | 497.3 | 517.2 | 526.8 |
| *Phymaturus tenebrosus* | 478.8 | **469.3** | 493.4 | 497.5 | 521.4 | 547.2 |
| *Phymaturus vociferator* | 496.4 | **494.1** | 495.4 | 513.3 | 528.3 | 520.4 |
| *Phymaturus zapalensis* | **378.0** | 378.4 | 390.9 | 423.9 | 446.0 | 467.8 |
| *Stenocercus aculeatus* | 150.3 | 149.1 | **146.0** | 149.0 | 148.4 | 151.8 |
| *Stenocercus angel* | 309.7 | **281.4** | 292.0 | 330.7 | 333.6 | 329.7 |
| *Stenocercus angulifer* | 624.4 | 384.8 | **364.5** | 385.1 | 387.5 | 389.5 |
| *Stenocercus apurimacus* | 329.1 | 338.7 | **243.2** | 255.6 | 260.9 | 274.0 |
| *Stenocercus azureus* | 348.0 | 344.6 | **342.7** | 343.1 | 343.1 | 343.2 |
| *Stenocercus boettgeri* | 239.2 | 243.6 | 236.9 | **234.5** | 237.6 | 246.6 |
| *Stenocercus cadlei* | 1079.7 | 1065.2 | **853.9** | 922.0 | 934.4 | 962.0 |
| *Stenocercus caducus* | 689.2 | 860.4 | **668.9** | 684.6 | 689.4 | 687.2 |
| *Stenocercus chota* | 224.9 | 230.4 | 270.4 | **202.1** | 216.2 | 236.4 |
| *Stenocercus chrysopygus* | 351.6 | **317.3** | 345.2 | 364.7 | 345.3 | 366.3 |
| *Stenocercus doellojuradoi* | 396.2 | **390.9** | 398.1 | 405.2 | 404.5 | 404.8 |
| *Stenocercus dumerilii* | 456.4 | 450.9 | **437.1** | 468.5 | 472.1 | 474.5 |
| *Stenocercus empetrus* | **319.4** | 330.4 | 329.1 | 339.2 | 353.2 | 358.5 |
| *Stenocercus erythrogaster* | **411.5** | 438.4 | 427.9 | 416.5 | 416.5 | 413.6 |
| *Stenocercus festae* | 533.8 | 530.3 | **524.4** | 533.5 | 537.8 | 532.8 |
| *Stenocercus fimbriatus* | 608.2 | 591.5 | 591.3 | **589.5** | 590.8 | 591.8 |
| *Stenocercus guentheri* | 1182.1 | **876.3** | 895.1 | 939.4 | 942.3 | 981.9 |
| *Stenocercus huancabambae* | **280.5** | 296.5 | 307.6 | 309.1 | 323.7 | 323.2 |
| *Stenocercus humeralis* | 234.5 | 227.1 | **217.4** | 233.1 | 235.2 | 233.7 |
| *Stenocercus imitator* | **319.6** | 323.5 | 343.8 | 348.3 | 351.4 | 355.8 |
| *Stenocercus iridescens* | 911.5 | 865.4 | **859.8** | 913.6 | 862.8 | 947.0 |
| *Stenocercus latebrosus* | 171.6 | 173.0 | 202.3 | **162.3** | 175.6 | 175.2 |
| *Stenocercus limitaris* | 175.2 | 173.9 | **172.9** | 182.7 | 179.2 | 174.9 |
| *Stenocercus marmoratus* | 391.2 | 341.18 | **310.5** | 338.1 | 337.2 | 335.2 |
| *Stenocercus melanopygus* | **292.1** | 304.1 | 320.0 | 312.6 | 333.9 | 327.0 |
| *Stenocercus modestus* | 131.6 | 130.9 | 132.1 | 134.3 | **128.4** | 135.3 |
| *Stenocercus ochoai* | 247.3 | 247.5 | 237.2 | **233.0** | 234.0 | 241.9 |
| *Stenocercus ornatissimus* | 236.5 | 241.4 | 231.8 | **220.6** | 239.0 | 237.2 |
| *Stenocercus ornatus* | 455.7 | 406.2 | **350.2** | 356.7 | 354.5 | 384.4 |
| *Stenocercus pectinatus* | 325.7 | 321.4 | 314.8 | **310.7** | 313.2 | 323.4 |
| *Stenocercus percultus* | 346.1 | 290.4 | **269.6** | 274.1 | 275.1 | 274.6 |
| *Stenocercus prionotus* | 501.0 | **490.5** | 495.2 | 500.4 | 502.1 | 502.9 |
| *Stenocercus puyango* | 268.3 | **202.0** | 205.1 | 209.9 | 213.2 | 222.6 |
| *Stenocercus rhodomelas* | **287.1** | 290.4 | 318.4 | 353.2 | 350.2 | 349.5 |
| *Stenocercus roseiventris* | 899.6 | 899.6 | **895.9** | 899.6 | 899.6 | 899.6 |
| *Stenocercus sinesaccus* | 234.8 | 234.6 | 198.2 | 195.5 | **192.7** | 194.8 |
| *Stenocercus torquatus* | 271.8 | 273.2 | 216.9 | **210.3** | 213.9 | 212.9 |
| *Stenocercus trachycephalus* | 575.2 | 573.5 | **573.0** | 573.2 | 571.6 | 567.6 |
| *Stenocercus variabilis* | 229.8 | **226.2** | 239.6 | 232.2 | 231.1 | 238.2 |
| *Stenocercus varius* | 263.2 | **261.6** | 277.2 | 296.5 | 289.5 | 301.1 |

**SUPPLEMENTARY FIGURES**


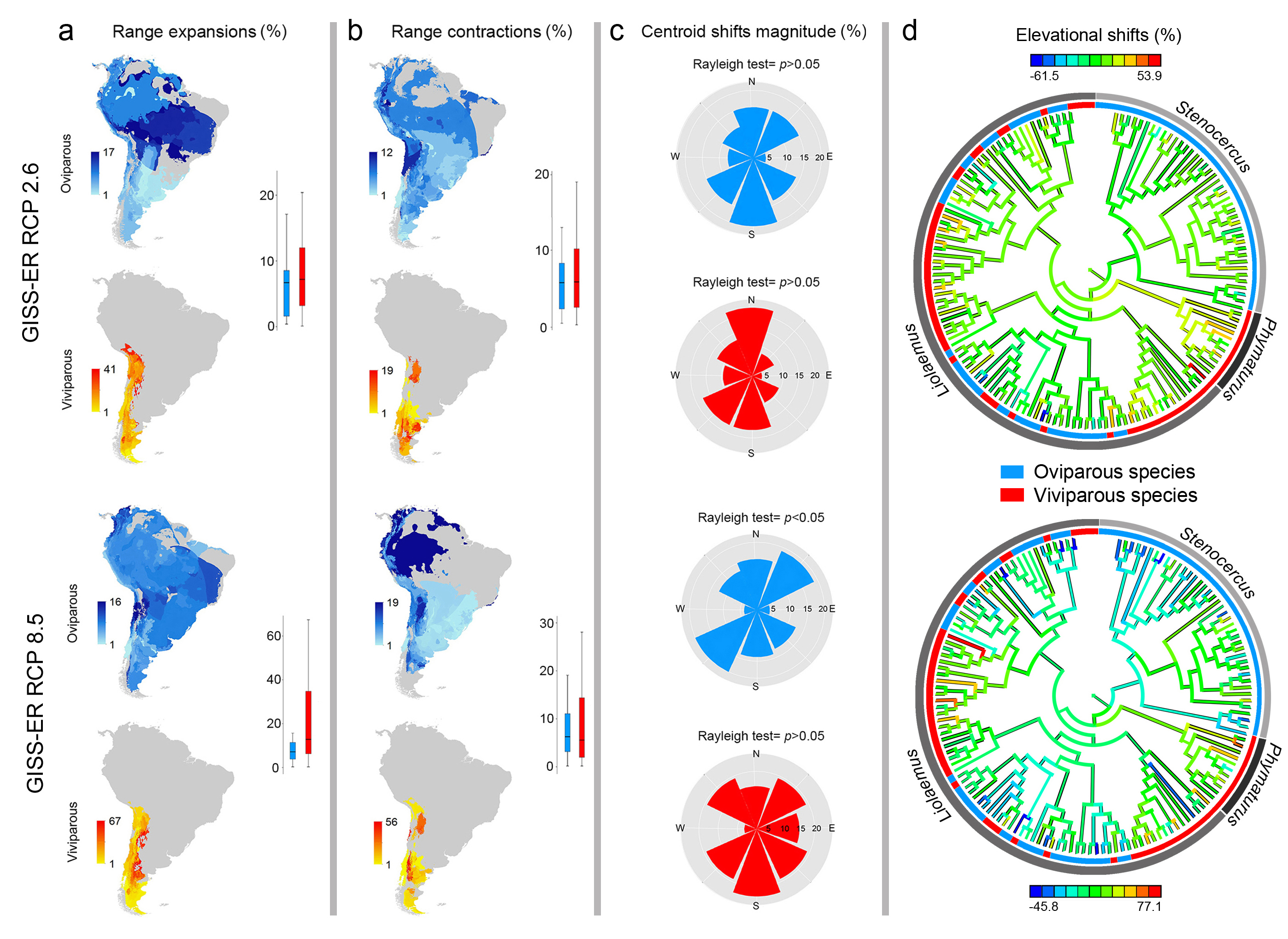


**Figure S1. Predicted impacts of climate change on the distribution of species in study under the GISS-ER model, considering the minimum (RCP 2.6) and maximum (RCP 8.5) scenario of greenhouse gases emissions.** Represented by a) expansions and b) contractions of ranges, c) variation in the direction of the geographic displacement and d) elevational shifts represented in the phylogeny of species in study, where the external ring shows oviparous (blue) and viviparous (red) species. The maps were created using ArcGIS 10.4.1 (Esri, Redlands, CA).


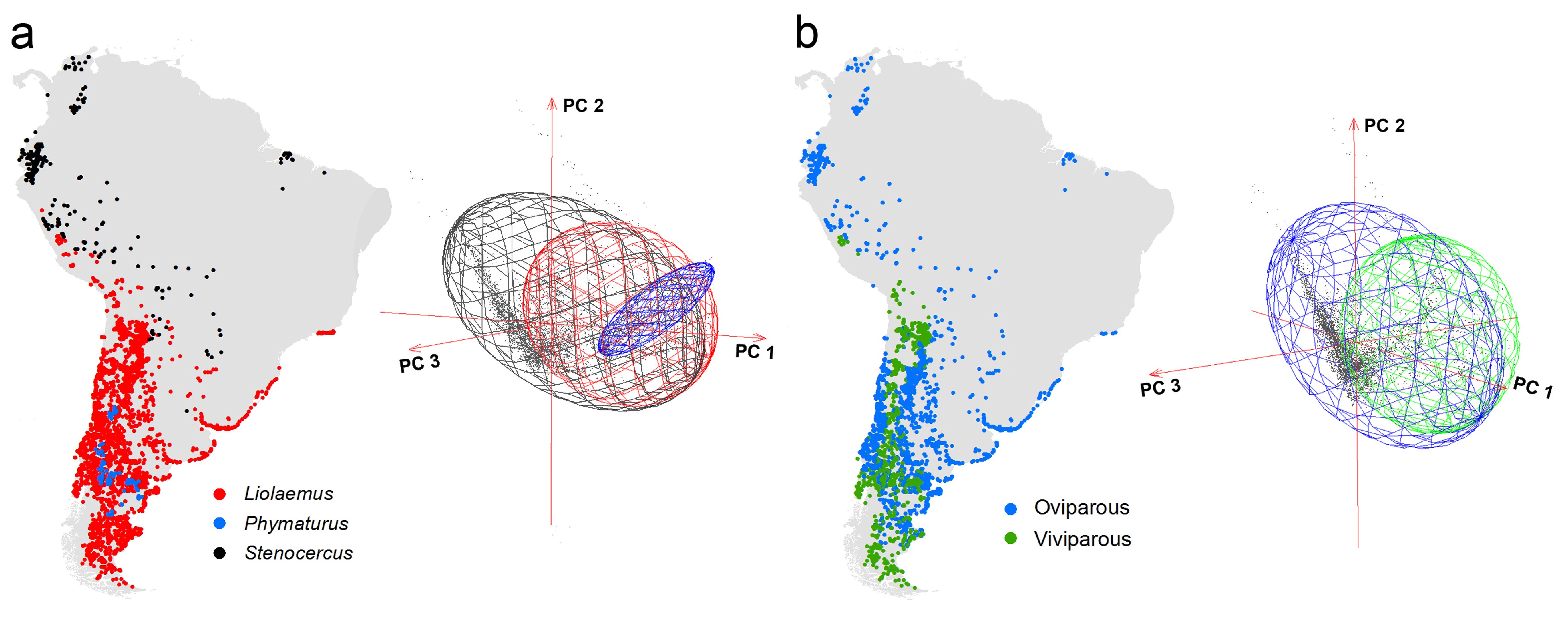


**Figure S2. Species distribution in the geographic and environmental space.** **a)** Distribution of *Liolaemus* (red points and ellipsoid), *Phymaturus* (blue points and ellipsoid), and *Stenocercus* lizards (gray points and ellipsoid) and **b)** its reproductive mode, oviparous (blue points and ellipsoid) and viviparous (green points and ellipsoid). The geographic space (gray map) is represented by latitude and longitude and the species’ occurrences in South America and the environmental space as the values (dark gray points) of the first three principal components (PC 1, 2, 3; red lines) for a three-dimensional environmental visualization of ecological niches. The maps were created using ArcGIS 10.4.1 (Esri, Redlands, CA). The 3d graphs were created using NicheA software [http://nichea.sourceforge.net](http://nichea.sourceforge.net/)
